# Association Between Organizational Quality and Out-of-Network Primary Care Among Accountable Care Organizations That Care for High vs Low Proportions of Patients of Racial and Ethnic Minority Groups

**DOI:** 10.1001/jamahealthforum.2022.0575

**Published:** 2022-04-15

**Authors:** Shivani Bakre, Nicholas Moloci, Edward C. Norton, Valerie A. Lewis, Yajuan Si, Sunny Lin, Emily J. Lawton, Lindsey A. Herrel, John M. Hollingsworth

**Affiliations:** 1 Department of Epidemiology, Johns Hopkins University, Baltimore, Maryland; 2 Department of Health Policy and Management, University of North Carolina, Chapel Hill; 3 Department of Health Management and Policy, University of Michigan, Ann Arbor; 4 Survey Research Center, Institute for Social Research, University of Michigan, Ann Arbor; 5 Department of Health Management and Policy, OHSU-PSU School of Public Health, Portland, Oregon; 6 Dow Division of Health Services Research, Department of Urology, University of Michigan, Ann Arbor

## Abstract

**Importance:**

Medicare accountable care organizations (ACOs) that disproportionately care for patients of racial and ethnic minority groups deliver lower quality care than those that do not, potentially owing to differences in out-of-network primary care among them.

**Objective:**

To examine how organizational quality is associated with out-of-network primary care among ACOs that care for high vs low proportions of patients of racial and ethnic minority groups.

**Design, Setting, and Participants:**

A retrospective cohort study was conducted between March 2019 and October 2021 using claims data (2013 to 2016) from a national sample of Medicare beneficiaries. Among beneficiaries who were assigned to 1 of 528 Medicare ACOs, a distinction was made between those treated by organizations that cared for high (vs low) proportions of patients of racial and ethnic minority groups. For each ACO, the amount of out-of-network primary care that it delivered annually was determined. Multivariable models were fit to evaluate how the quality of care that beneficiaries received varied by the proportion of care provided to patients of racial and ethnic minority groups by the ACO and its amount of out-of-network primary care.

**Exposures:**

The degree of care provided to patients of racial and ethnic minority groups by the ACO and its amount of out-of-network primary care.

**Main Outcomes and Measures:**

The ACO quality assessed with 5 preventive care services and 4 utilization metrics.

**Results:**

Among 3 955 951 beneficiary-years (2 320 429 [58.7%] women; 71 218 [1.8%] Asian, 267 684 [6.8%] Black, 44 059 [1.1%] Hispanic, 4922 [0.1%] North American Native, and 3 468 987 [87.7%] White individuals and 56 157 [1.4%] of Other race and ethnicity), those assigned to ACOs serving many patients of racial and ethnic minority groups at the mean level of out-of-network primary care were less likely than those assigned to ACOs serving fewer patients of racial and ethnic minority groups to receive diabetic retinal examinations (predicted probability, 49.4% [95%CI, 49.0%-49.7%] vs 51.6% [95% CI, 51.5%-51.8%]), glycated hemoglobin testing (predicted probability, 58.5% [95% CI, 58.2%-58.5%] vs 60.4% [95% CI, 60.3%-60.6%]), or low-density lipoprotein cholesterol testing (predicted probability, 85.2% [95% CI, 85.0%-85.5%] vs 86.0% [95% CI, 85.9%-86.1%]). They were also more likely to experience all-cause 30-day readmissions (predicted probability, 16.4% [95% CI, 16.1%-16.7%] vs 15.7% [95% CI, 15.6%-15.8%]). However, as the level of out-of-network primary care decreased, these gaps closed substantially, such that beneficiaries at ACOs that served many and fewer patients of racial and ethnic minority groups in the lowest percentile of out-of-network primary care received care of comparable quality.

**Conclusions and Relevance:**

This large cohort study found that quality performance among ACOs serving many patients of racial and ethnic minority groups was negatively associated with their level of out-of-network primary care.

## Key Points

**Question:**

How is the quality of care delivered by a Medicare accountable care organization (ACO) associated with the level of out-of-network primary care among organizations that care for high vs low proportions of patients of racial and ethnic minority groups?

**Findings:**

In this retrospective cohort study of 3 955 951 beneficiary-years within 528 Medicare ACOs, the ACOs that cared for more patients of racial and ethnic minority groups had significantly higher rates of out-of-network primary care than those that cared for fewer patients of racial and ethnic minority groups. The level of out-of-network primary care was negatively associated with performance among ACOs with many patients of racial and ethnic minority groups across most quality metrics examined.

**Meaning:**

The study findings suggest that organizational efforts to limit out-of-network primary care at ACOs caring for many patients of racial and ethnic minority groups could serve as a tangible, accessible corrective for reducing health care disparities among the populations that they serve.

## Teaser

This cohort study examines the association of organizational quality with out-of-network primary care among accountable care organizations that care for high vs low proportions of patients of racial and ethnic minority groups.

## Introduction

Inequities in health care quality affect patients of racial and ethnic minority groups over a range of diseases and services.^1,2,3,4,5,6,7,8,9,10,11^ While not centered on racial and ethnic minority communities, there are reasons to believe that the recent accountable care initiatives from the US Centers for Medicare & Medicaid Services (CMS), which aim to improve health care quality for all Medicare beneficiaries, may help reduce these inequities. Through potential shared savings payments, accountable care organizations (ACOs) incentivize clinicians to coordinate their care, preventing duplicative services and medical errors.^12^ Yet, while there have been quality gains among patients of racial and ethnic minority groups who receive care from Medicare ACOs, organizations that disproportionately care for these beneficiaries still perform poorer on quality metrics than those that do not.^13^

One potential explanation for this finding is associated with higher levels of out-of-network primary care among ACOs serving many patients of racial and ethnic minority groups. When this care is delivered outside an ACO’s contracting network, the organization’s care coordination capabilities, which are critical to its performance, could be impeded. Prior research demonstrates significant increases in quarterly per beneficiary spending for every percentage point increase in an organization’s out-of-network primary care.^14^ Further, ACOs with higher levels of out-of-network primary care also care for more patients of racial and ethnic minority groups.^14^ Insofar as out-of-network primary care is associated with poorer quality and treatment of more patients of racial and ethnic minority groups, mechanisms to reduce it could be used by CMS and ACO administrators to reduce health care inequities.

In this context, we analyzed medical claims data from a national sample of Medicare beneficiaries, assessing whether the level of out-of-network primary care accounted for lower-quality achievement among ACOs that care for high (vs low) proportions of patients of racial and ethnic minority groups. After distinguishing between ACOs based on the populations that they served, we characterized organizational differences between ACOs that served many vs fewer patients of racial and ethnic minority groups. We then quantified the quality gap between ACOs that served many vs fewer patients of racial and ethnic minority groups across several quality metrics at the mean level of out-of-network primary care. Lastly, we evaluated changes in this quality gap across percentiles of ACO out-of-network primary care. We hypothesized that ACOs that served many patients of racial and ethnic minority groups would, on average, perform worse on the quality metrics than ACOs that served fewer. Additionally, we posited that ACOs at the highest level of out-of-network primary care would have the largest quality gaps and that these gaps would be progressively smaller as the level of out-of-network primary care decreased.

## Methods

### Data Sources and Study Population

We conducted a retrospective cohort study between March 2019 and October 2021 that analyzed claims data from January 1, 2013, to December 31, 2016, that were contained in the Medicare Beneficiary Summary, Medicare Provider and Analysis Review, Outpatient, and Carrier Research Identifiable Files (RIFs) for a national random 20% sample of Medicare beneficiaries. For inclusion, we required beneficiaries to be at least age 66 years and have continuous Parts A and B enrollment during a given study year and the prior year (to allow for comorbidity adjustment). We excluded beneficiaries who were enrolled in a Medicare Advantage plan (3 625 858; 744 261 [20.5%] of racial and ethnic minority groups), lived outside the US, or had end-stage kidney disease. Additionally, we merged the claims data of beneficiaries with the Shared Savings Program (SSP) beneficiary-level RIF to ascertain which beneficiaries were assigned to a given ACO during a study year. To further characterize ACOs, we supplemented these data with the SSP Provider-level RIF, the Leavitt Partners ACO Database,^13^ and the SSP ACO public-use file to capture characteristics, such as leadership structure, contract start date, years of participation, risk-sharing arrangement with CMS, and the number of lives covered.

We obtained approval for this study from the institutional review board of University of Michigan, which deemed it exempt from oversight and informed consent because all patient data were deidentified. This article adhered to the Strengthening the Reporting of Observational Studies in Epidemiology (STROBE) reporting guideline for cohort studies.

### Determining the Proportion of Care Provided by an ACO to Patients of Racial and Ethnic Minority Groups

To determine the proportion of patients of racial and ethnic groups served by an ACO, we divided its number of assigned Asian, Black, Hispanic, and North American Native beneficiaries and beneficiaries of Other or unknown race and ethnicity by its total number of assigned beneficiaries. We distinguished race and ethnicity using the Beneficiary Race Code contained in the Medicare Beneficiary Summary File. This categorical variable differentiates between beneficiaries of Asian, Black, Hispanic, North American Native, White, Other, and unknown race and ethnicity. We calculated the proportion of patients of racial and ethnic minority groups served by an ACO once across all study years. Consistent with prior research, we defined a given ACO as having a high proportion if its proportion of patients of racial and ethnic minority groups was in the top quartile of the overall proportion distribution of patients of racial and ethnic minority groups. We defined those in the 3 bottom quartiles as ACOs with a low proportion of patients of racial and ethnic minority groups.^13^

### Calculating Out-of-Network Primary Care

We calculated out-of-network primary care using a 2-step process.^14^ In the first step, we identified preventive care visits, annual wellness visits, and other outpatient visits from beneficiaries’ claims using *Current Procedural Terminology* codes 99201-15, G0402, and G0438-9, respectively. Through relevant Medicare Provider Specialty codes (for general practice, family practice, internal medicine, or geriatric medicine) appearing on these claims, we distinguished primary care visits for these services. In the second step, we determined the level of out-of-network care for each ACO during every study year, dividing the number of primary care visits that its beneficiaries made to clinicians outside its contacting network by the total number of primary care visits made by its beneficiaries.

### Assessing ACO Quality Performance

We assessed ACO quality annually using 5 preventive care and 4 utilization metrics. The preventive care metrics included glycated hemoglobin testing, low-density lipoprotein (LDL) cholesterol testing, retinal examinations, a composite measure for receipt of all 3 tests, and screening mammography. We calculated glycated hemoglobin levels, LDL cholesterol testing, and retinal examination metric performance among those aged 66 to 75 years with a previous diagnosis of diabetes. We calculated screening mammography metric performance among women aged 66 to 69 years with no history of mastectomy.

The utilization metrics included the rates of preventable hospitalization for 2 ambulatory care-sensitive conditions (congestive heart failure [CHF] and chronic obstructive pulmonary disease [COPD]/asthma), all-cause 30-day readmission, and emergency department (ED) visit. Both CHF and COPD/asthma are recognized by the Agency for Healthcare Research and Quality as conditions for which “good outpatient care can potentially prevent the need for hospitalization.^15^” Moreover, SSP contracts required reporting hospitalizations for these conditions.^16^ We defined 30-day readmissions based on the SSP specifications.^16^

### Statistical Analysis

For all analyses, the beneficiary-year served as the unit of analysis. In the initial analytic step, we used *t* tests for continuous variables and χ^2^ tests for categorical variables to compare beneficiaries assigned to an ACO serving more or fewer patients of racial and ethnic minority groups. Specifically, we made comparisons with respect to age, sex, race and ethnicity (Asian, Black, Hispanic, North American Native, White, Other, and unknown), urban/rural residence, dual eligibility status, socioeconomic status (based on a ZIP code–level composite measure^17^), number of primary care visits, and level of comorbid illness (defined using hierarchical condition categories risk score^18^). Next, we evaluated for organizational differences between ACOs serving more or fewer patients of racial and ethnic minority groups, including differences in their leadership structure (ie, physician-led vs hospital-led vs physician-hospital partnership), years of program participation, contract start date, type of risk agreement with CMS (1-sided vs 2-sided risk), region, number of primary care clinicians (PCCs) per 1000 beneficiaries, the number of lives covered, and level of out-of-network primary care.

Following this, we fit a series of multilevel models to characterize the variation in out-of-network primary care across ACOs. In particular, we fit a multilevel model with random intercepts varying across ACOs. The principal model was a random intercept model with no explanatory variables included (empty model), which allowed us to understand the basic partitioning of the data’s variability between the beneficiary and ACO levels. We introduced an ACO identifier (the ACO identification number from the SSP Beneficiary-level and Provider-level RIFs) as a random effect. To try and explain the observed variation in out-of-network primary care, we added the beneficiary-level and ACO-level variables described in the preceding paragraph.

We then evaluated whether an ACO-assigned beneficiary’s attainment of 1 of the quality metrics was associated with their ACO’s proportion of patients of racial and ethnic minority groups and how that may have been modified by the ACO’s level of out-of-network primary care. We did this by fitting a series of multivariable logistic regression models, controlling for the potential beneficiary-level and organization-level confounders described previously. We also included study year and health care market (defined by hospital referral region boundaries^19^) fixed effects. Given the correlated data structure (beneficiaries nested within ACOs), we calculated robust standard errors.^20^ To express the strength of the association between quality metric attainment and an ACO’s proportion of patients of racial and ethnic minority groups, we estimated predicted probabilities at the mean level of out-of-network primary care and for each absolute percentile of out-of-network primary care, stratified by the organization’s proportion of patients of racial and ethnic minority groups.

### Sensitivity Analyses

To assess the robustness of our findings, we conducted 4 sensitivity analyses. First, given how we distinguished an ACO’s proportion of patients of racial and ethnic minority groups (ie, whether above or below the top quartile of patients of racial and ethnic minority groups), it was possible for ACOs serving more or fewer patients of racial and ethnic minority groups to be close in percentile rank. For example, an ACO serving more patients of racial and ethnic minority groups could be at the 80th percentile and an ACO serving fewer patients of racial and ethnic minority groups at the 70th percentile. Because the meaningfulness of this difference was unclear, we reestimated the models for each quality metric, comparing ACOs serving more patients of racial and ethnic minority groups (top quartile) with those serving fewer patients of racial and ethnic minority groups (bottom quartile), examining whether the findings from this analysis were consistent with those of the main analysis.

Second, we hypothesized that ACOs with high levels of out-of-network primary care may differ organizationally from those closer to the mean level of out-of-network primary care, potentially confounding the results. In a post hoc analysis comparing organizational characteristics at each percentile of out-of-network primary care, we noted differences between ACOs below and above the 60% and 90% percentiles of out-of-network primary care. Thus, we reestimated the models for each quality metric on 2 restricted samples (out-of-network primary care from 0% to 60% and 0% to 90%) and compared these results with those from the main analysis, which included out-of-network primary care that ranged from 0% to 100%.

Third, because of the potential for state-level differences in ACO implementation and in the distribution of selected outcomes, we reestimated the models, including state fixed effects. Finally, we analyzed the covariate of beneficiary race and ethnicity (patients of racial and ethnic minority groups vs White patients) within ACOs serving more or fewer patients of racial and ethnic minority groups to assess whether patients of racial and ethnic minority groups have worse outcomes than their White counterparts or if ACOs serving more patients of racial and ethnic minority groups may be uniquely disadvantaged, resulting in poor quality performance for all patients regardless of race and ethnicity.

We performed all analyses using SAS, version 9.4 (SAS Institute), and Stata, version 15/MP (StataCorp). All of the statistical tests were 2-tailed, and we set the probability of a type 1 error at .05.

## Results

In total, we identified 3 955 951 beneficiary-years that were assigned to 528 ACOs, 132 (25%) of which cared for a high proportion of patients who identified as belonging to a racial or ethnic minority group. Among this cohort, 474 229 had a diagnosis of CHF (59 112 [12.5%] of whom identified as belonging to a racial and ethnic minority group), 485 207 had COPD (49 112 [10.1%] identified as belonging to a racial and ethnic minority group), and 1 118 942 had diabetes (198 866 [17.8%] identified as belonging to a racial and ethnic minority group). Table 1 displays differences between beneficiaries assigned to ACOs serving more vs fewer patients of racial and ethnic minority groups. Unsurprisingly, beneficiaries assigned to ACOs serving more patients of racial and ethnic minority groups were more likely to identify as belonging to a racial and ethnic minority group than those assigned to ACOs serving fewer patients of racial and ethnic minority groups (30.0% vs 8.4%, respectively; *P* < .001). They were also more likely to have the lowest socioeconomic status (36.1% vs 32.7%, respectively; *P* < .001), have a higher hierarchical condition categories risk score (mean [SD] score, 1.2 [1.0] vs 1.1 [0.9], respectively; *P* < .001), have dual Medicare and Medicaid eligibility (20.3% vs 8.0%, respectively; *P* < .001), and live in an urban area with more than 1 million residents (76.2% vs 47.4%, respectively; *P* < .001). While there were also statistically significant differences between ACOs serving more or fewer patients of racial and ethnic minority groups regarding sex distribution and the mean number of PCC visits among the assigned beneficiaries, these differences were not clinically meaningful.

**Table 1.** Differences in Beneficiary Characteristics Between ACOs Serving More or Fewer Patients of Racial and Ethnic Minority Groups

Characteristic	No. (%)		*P* value
	ACO serving fewer patients of racial and ethnic minority groups	ACO serving more patients of racial and ethnic minority groups	
Beneficiary-years	3 249 573	706 378	NA
Unique beneficiaries	1 674 281	401 213	NA
Age, mean (SD), y	76.3 (7.6)	76.3 (7.8)	.35
Sex			
Female	1 896 988 (58.4)	423 441 (59.9)	<.001
Male	1 352 585 (41.6)	282 937 (40.1)	
Race and ethnicity			
Asian	27 235 (0.8)	43 983 (6.2)	<.001
Black	154 546 (4.8)	113 138 (16.0)	
Hispanic	20 797 (0.6)	23 262 (3.3)	
North American Native	4357 (0.1)	565 (0.1)	
White	2 974 308 (91.6)	494 679 (70.0)	
Other	33 846 (1.0)	22 311 (3.2)	
Unknown	34 484 (1.1)	8440 (1.2)	
Urban/rural place of residence			
Metropolitan	485 441 (14.9)	40 167 (5.7)	<.001
Urban			
>1 Million	154 048 (47.4)	538 433 (76.2)	
<1 Million	1 164 501 (35.9)	122 976 (17.4)	
Rural	59 410 (1.8)	4775 (0.7)	
Socioeconomic status^a^			
Low	1 032 833 (32.7)	249 631 (36.1)	<.001
Middle	1 098 896 (34.8)	183 216 (26.5)	
High	1 025 079 (32.5)	258 058 (37.4)	
Dual eligibility	261 286 (8.0)	143 153 (20.3)	<.001
HCC risk score, mean (SD)	1.1 (0.9)	1.2 (1.0)	<.001
No. of primary care visits^b^ (SD)	3.3 (3.0)	3.7 (3.7)	<.001

Abbreviations: ACO, accountable care organization; HCC, hierarchical condition categories; NA, not applicable.

^a^ A total of 46 551 and 8552 unique beneficiaries assigned to ACOs serving fewer and more patients of racial and ethnic minority groups, respectively, had missing socioeconomic status data.

^b^ No. of primary care visits per person per year.

Organizational differences between ACOs serving more or fewer patients of racial and ethnic minority groups are displayed in Table 2. Compared with ACOs serving fewer patients of racial and ethnic minority groups, those ACOs serving more were more likely to be physician led (57.5% vs 43.7%, respectively; *P* < .001) and cover fewer lives (mean [SD] number, 9058 [9888] vs 13 886 [17 207], respectively; *P* < .001). They were less likely to be in the Midwest (9.7% vs 32.3%, respectively; *P* < .001). Notably, ACOs serving more patients of racial and ethnic minority groups had significantly higher rates of out-of-network primary care than those serving fewer (12.7% vs 10.2%, respectively; *P* < .001).

**Table 2.** Organizational Differences Between ACOs Serving More or Fewer Patients of Racial and Ethnic Minority Groups

Characteristic	ACO by proportion of patients of racial and ethnic minority groups, No. (%)		*P* value
	Low	High	
No. of ACOs	396	132	
Leadership structure^a^			
Physician led	174 (43.7)	76 (57.5)	<.001
Hospital led	79 (18.7)	25 (18.9)	
Physician-hospital partnership	141 (37.3)	31 (23.6)	
Type of risk agreement with CMS^b^			
1-Sided risk	389 (97.5)	130 (96.7)	.38
2-Sided risk	17 (2.1)	9 (3.3)	.18
Contract start date			
2012/2013	164 (56.7)	56 (54.2)	.39
2014	78 (21.1)	41 (32.2)	<.001
2015	75 (14.4)	14 (7.8)	<.001
2016	79 (7.8)	21 (5.8)	.22
Region^c^			
Northeast	93 (27.9)	39 (31.2)	<.001
South	114 (23.1)	47 (30.1)	
Midwest	98 (32.3)	13 (9.7)	
West	88 (16.8)	32 (29.0)	
SSP participation, mean (SD), y	1.3 (1.2)	1.3 (1.2)	.79
No. of lives covered, mean (SD)	13 886 (17 207)	9058 (9888)	<.001
No. of PCCs per 1000 beneficiaries, mean (SD)	247. 6 (763.3)	311.1 (617.3)	.16
Out-of-network primary care, mean % (SD)	10.2 (14.5)	12.7 (15.8)	<.001

Abbreviations: ACO, accountable care organization; CMS, US Centers for Medicare & Medicaid Services; PCC, primary care clinician; SSP, Shared Savings Program.

^a^ Two ACOs are missing from the leadership structure category because of missing data from Torch Insight.

^b^ The percentages for types of risk agreement with CMS do not add up to 100% because ACOs shifted contracts between 1-sided and 2-sided risk during the study period.

^c^ Four ACOs were excluded from the region category because of having an administrative home in a US territory that does not map to a US Census region.

The overall level of out-of-network primary care was low (mean [SD], 10.7% [14.8%]); however, there was variability across ACOs (range, 1.2%-100%). eTable 1 in the Supplement displays the percentage of total variance in out-of-network primary care attributable to the beneficiary and ACO levels from the multilevel models. Based on the empty model, the beneficiary and ACO levels accounted for 29.4% and 70.6%, respectively, of the total variation. Between the empty and full models, the variation attributable to the beneficiary decreased from 29.4% to 27.4%, and the variation attributable to ACOs increased from 70.6% to 72.6%. eTable 2 in the Supplement displays the full multilevel model, demonstrating independent associations between beneficiary-level and ACO-level factors and out-of-network primary care.

On multivariable analysis, beneficiaries assigned to ACOs serving more patients of racial and ethnic minority groups at the mean level of out-of-network primary care were less likely than those assigned to ACOs serving fewer patients of racial and ethnic minority groups to receive preventive care services. Specifically, as shown in Table 3, they were less likely to receive any diabetes testing (predicted probability, 49.4% [95%CI, 49.0%-49.7%] vs 51.6% [95% CI, 51.5%-51.8%]), diabetic retinal examinations (predicted probability, 58.5% [95% CI, 58.2%-58.5%] vs 60.4% [95% CI, 60.3%-60.6%]), glycated hemoglobin testing (predicted probability, 58.5% [95% CI, 58.2%-58.5%] vs 60.4% [95% CI, 60.3%-60.6%]), and LDL cholesterol testing (predicted probability, 85.2% [95% CI, 85.0%-85.5%] vs 86.0% [95% CI, 85.9%-86.1%]) at the mean level of out-of-network primary care. There was also a marginally significant difference in the receipt of screening mammography (predicted probability, 73.7% [95% CI, 73.3%-74.0%] vs 75.3% [95% CI, 75.2%-75.4%]). With respect to the utilization metrics, beneficiaries in ACOs serving more patients of racial and ethnic minority groups were more likely to experience an all-cause 30-day readmission (predicted probability, 16.4% [95% CI, 16.1%-16.7%] vs 15.7% [95% CI, 15.6%-15.8%]) then those in ACOs serving fewer patients of racial and ethnic minority groups at the mean level of out-of-network primary care. They were also more likely to be hospitalized for CHF (predicted probability, 1.19% [95% CI, 1.16%-1.23%] vs 1.13% [95% CI, 1.12%-1.15%]), but the absolute difference between the 2 groups was small. Full model results are displayed in eTable 3 in the Supplement.

**Table 3.** Predicted Probabilities of Preventive Services Receipt and Hospital Utilization for ACOs Serving More or Fewer Patients of Racial and Ethnic Minority Groups at the Mean Level of Out-of-Network Primary Care

Quality metric	ACO by proportion of patients of racial and ethnic minority groups, % (95% CI)		*P* value
	Low	High	
Preventive services			
All diabetes tests	51.6 (51.5-51.8)	49.4 (49.0-49.7)	<.001
Diabetic retinal examinations	60.4 (60.3-60.6)	58.5 (58.2-58.5)	<.001
Glycated hemoglobin testing	90.2 (90.1-90.3)	89.2 (89.0-89.4)	<.001
LDL cholesterol testing	86.0 (85.9-86.1)	85.2 (85.0-85.5)	<.001
Mammography	75.3 (75.2-75.4)	73.7 (73.3-74.0)	<.001
Utilization			
CHF hospitalizations	1.13 (1.12-1.15)	1.19 (1.16-1.23)	<.01
COPD/asthma hospitalizations	0.81 (0.80-0.81)	0.80 (0.78-0.83)	.79
All-cause 30-d readmissions	15.7 (15.6-15.8)	16.4 (16.1-16.7)	<.001
ED visits	31.6 (31.5-31.6)	31.6 (31.5-31.8)	.68

Abbreviations: ACO, accountable care organization; CHF, congestive heart failure; COPD, chronic obstructive pulmonary disease; ED, emergency department; LDL, low-density lipoprotein.

**Figure 1.**
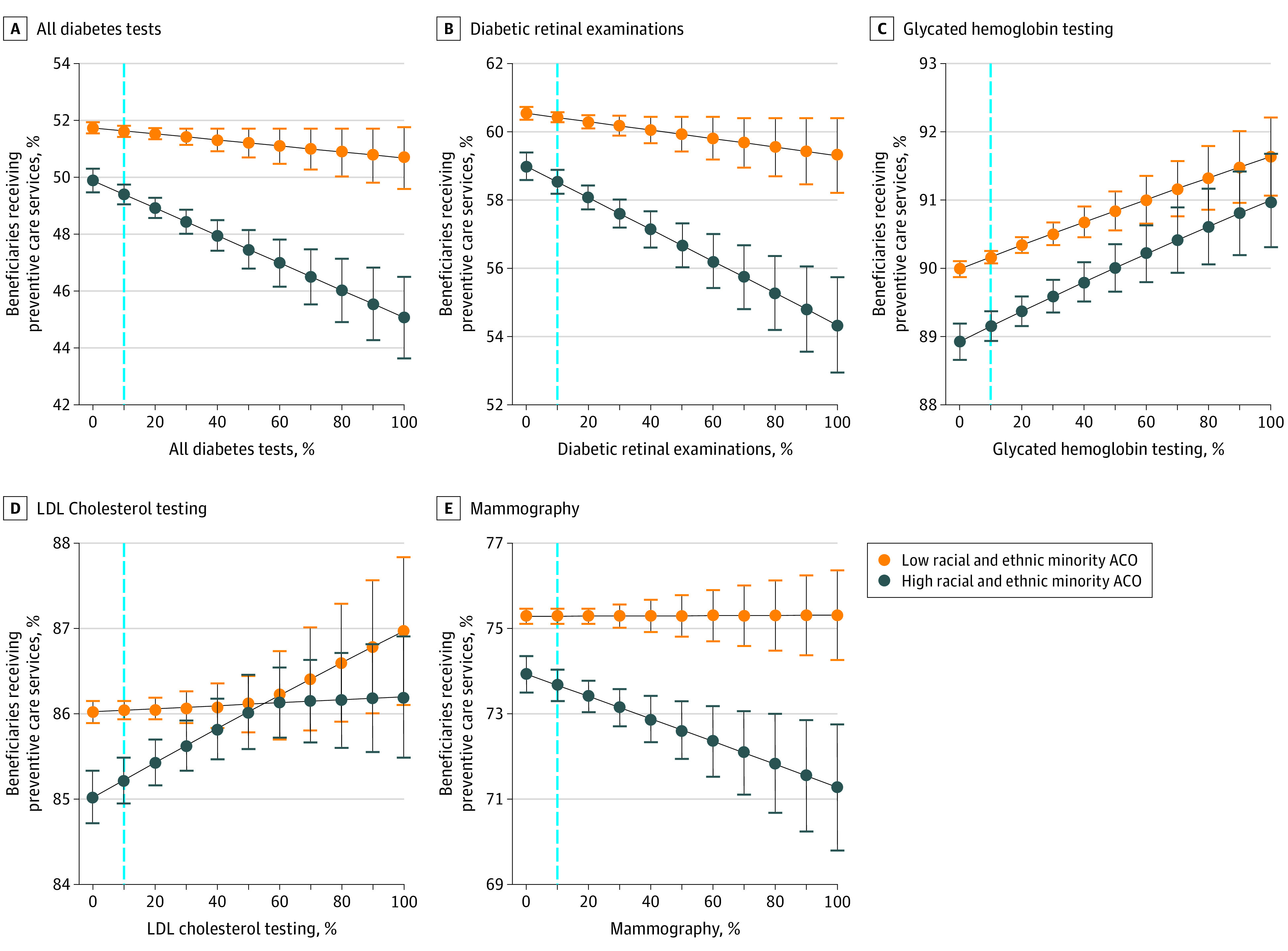
Predicted Probabilities for Different Preventive Care Services by Percentile of Out-of-Network Primary Care The x-axis represents the percentile of out-of-network primary care for each preventive care service. The dashed line is the mean-level of out-of-network primary care, which is approximately 10%. The error bars represent the 95% CIs. ACO indicates accountable care organization; LDL, low-density lipoprotein.

**Figure 2.**
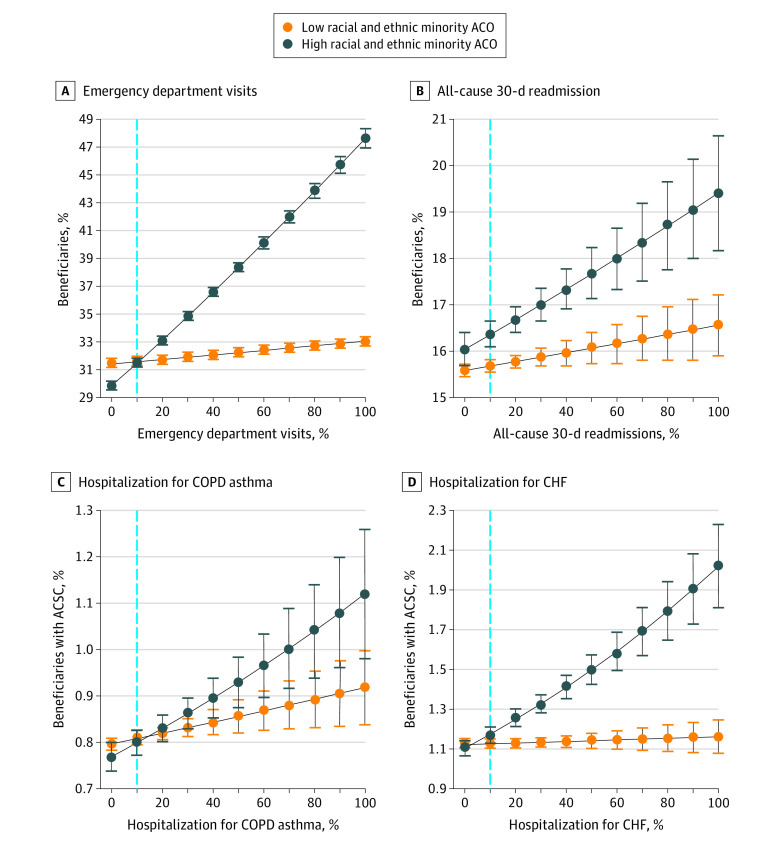
Predicted Probabilities for Different Hospital Utilization Metrics by Percentile of Out-of-Network Primary Care The x-axis represents the percentile of out-of-network primary care for each utilization metric. The dashed line is the mean-level of out-of-network primary care, which is approximately 10%. The error bars represent the 95% CIs. ACO indicates accountable care organization; ACSC, ambulatory care-sensitive condition; CHF, congestive heart failure; COPD, chronic obstructive pulmonary disease.

Figure 1 and Figure 2 display results from our multivariable models on quality attainment for the preventive and utilization metrics at increasing percentiles of out-of-network primary care as stratified by the proportion of patients of racial and ethnic minority groups at ACOs. The dashed line represents the mean level of out-of-network primary care across all ACOs. As the level of out-of-network primary care increases, the quality gap between ACOs serving more or fewer patients of racial and ethnic minority groups widens such that at the highest percentile of out-of-network primary care, ACOs serving more patients perform worse on 6 of 9 preventive and utilization metrics. As the level of out-of-network primary care decreases, the quality gap between ACOs serving more or fewer patients of racial and ethnic minority groups closes to the point that beneficiaries receive care, which is almost, if not entirely, comparable across the metrics examined.

Results from the sensitivity analyses are shown in eTable 4 and eFigures 1 through 9 in the Supplement. In eTable 4 in the Supplement, the findings were consistent with those of the main analysis when we reestimated predictive margin differences comparing the top and bottom quartiles of the overall proportion distribution of patients of racial and ethnic minority groups, restricted out-of-network primary care to ranges of 0% to 60% and 0% to 90%, and included state fixed effects. Additionally, eFigures 1 through 9 in the Supplement show that the differences in quality metric performance between ACOs serving more or fewer patients of racial and ethnic minority groups were not associated with within-ACO differences in the quality of care delivered to beneficiaries who identified as belonging to a racial or ethnic minority group and White beneficiaries.

## Discussion

This cohort study has 3 main findings. First, ACOs that care for high proportions of patients of racial and ethnic minority groups had, on average, greater levels of out-of-network primary care than those that care for lower proportions. Second, ACOs serving more patients of racial and ethnic minority groups performed poorer on quality (as measured by various preventive services and utilization metrics) than ACOs serving fewer patients. Third, as the level of out-of-network primary care increased for ACOs serving more patients of racial and ethnic minority groups, the quality gap between them and ACOs serving fewer patients widened. For instance, at the 90th percentile of out-of-network primary care, ACOs serving more patients of racial and ethnic minority groups performed worse than ACOs serving fewer patients on 6 of 9 quality metrics. This gap lessened as the level of out-of-network primary care decreased, such that the performance gaps of ACOs serving more or fewer patients of racial and ethnic minority groups were absent or much reduced at the 10th percentile of out-of-network primary care.

The finding that ACOs serving more patients of racial and ethnic minority groups have more out-of-network primary care than ACOs serving fewer patients is corroborated by a previous study that evaluated the association between out-of-network care among SSP ACOs and per beneficiary spending.^14^ Specifically, when compared with ACOs in the lower 3 quartiles of out-of-network primary and specialty care, those in the highest quartile cared for a larger proportion of Black and Hispanic beneficiaries and dually eligible beneficiaries.^14^ This study’s finding of poorer quality among ACOs serving more patients of racial and ethnic minority groups is also consistent with prior ACO-level analyses demonstrating that having a larger proportion of patients of racial and ethnic minority groups assigned with an organization was associated with worse performance, with 1 study showing differences across 25 of 33 publicly reported quality metrics.^13,21^ However, this study’s finding that the level of out-of-network primary care of an ACO serving more patients of racial and ethnic minority groups may moderate its quality of care is novel and potentially actionable.

One plausible explanation for why ACOs serving more patients of racial and ethnic minority groups with high levels of out-of-network primary care perform poorly on preventive services and hospital utilization metrics is associated with the central role that PCCs play as care coordinators. When beneficiaries assigned to an ACO receive care outside the organization, the care coordination capabilities of ACO-participating PCCs are hampered because they have few sight lines into and even less control over clinicians outside their contracting network. As a consequence, beneficiaries may fail to receive timely preventive services, potentially resulting in more nonurgent ED visits and potentially avoidable hospitalizations.

### Limitations

This study’s findings must be considered in the context of several limitations. First, we did not have information on the race and ethnicity of the clinicians with the contracting networks of the ACOs. A growing body of research shows that racially and ethnically concordant patient-clinician relationships are generally associated with improved clinical outcomes and enhanced satisfaction. To the extent that the contracting networks of ACOs serving more patients of racial and ethnic minority groups have low numbers of clinicians of racial and ethnic minority groups, then patients of racial and ethnic minority groups assigned to them may be compelled to seek their primary care out of network.

Second, we acknowledge the possibility of omitted variable bias. Clearly, the claims data that we used lack important patient characteristics like education level, income level and wealth, social support, and neighborhood resources. These social determinants of health, as well as other unmeasured factors, may help explain some of the findings. Third, our analysis included only SSP ACOs. As such, the findings may not be generalizable to other ACO models (eg, the Pioneer ACO Program, end-stage kidney disease seamless care organizations, and next-generation ACOs). Nonetheless, SSP ACOs account for more than 85% of Medicare ACOs. Finally, we cannot prove causality given the study’s observational design, and research is needed to determine if ACOs serving more patients of racial and ethnic minority groups that reduce out-of-network primary care also improve the quality of care that they deliver.

## Conclusions

The findings of this cohort study have potential policy implications. Namely, they suggest that organizational efforts to increase in-network primary care at ACOs serving more patients of racial and ethnic minority groups could serve as a tangible, accessible corrective for reducing health care disparities. There are numerous ways in which ACOs serving more patients of racial and ethnic minority groups could do this. For example, they could offer after-hours and weekend access to medical services for beneficiaries, which has been shown to improve care continuity and limit ED utilization and admissions for ambulatory care–sensitive conditions.^22,23^ Alternatively, ACOs serving more patients of racial and ethnic minority groups could use financial incentives, such as lower copayments, that encourage their assigned beneficiaries to see in-network primary care clinicians.^24^ Clearly, any of these efforts would need to be balanced with the preservation of patient choice, which is a defining characteristic of SSP ACOs.

## Notes

Supplement. **eTable 1**. Multilevel regression results, quantifying the percentage of residual variance in out-of-network primary care attributable to the beneficiary and ACO levels **eTable 2. **Full multilevel model, demonstrating independent associations between beneficiary- and ACO-level factors and out-of-network primary care **eTable 3. **Full multivariable logistic regression models, examining preventive services receipt and hospital utilization among beneficiaries assigned to high racial and ethnic minority ACOs at the mean level of out-of-network primary care **eTable 4. **Adjusted risk differences on sensitivity analyses **eFigure 1. **Predicted Percent of Beneficiaries Receiving All Diabetes Tests at Each Percentile of Out-of-Network Primary Care Stratified by Racial and Ethnic Minority and White Beneficiaries in Low and High Racial and Ethnic Minority ACOs **eFigure 2. **Predicted Percent of Beneficiaries Receiving Diabetic Retinal Examinations at Each Percentile of Out-of-Network Primary Care Stratified by Racial and Ethnic Minority and White Beneficiaries in Low and High Racial and Ethnic Minority ACOs **eFigure 3. **Predicted Percent of Beneficiaries Receiving Glycated Hemoglobin Testing at Each Percentile of Out-of-Network Primary Care Stratified by Racial and Ethnic Minority and White Beneficiaries in Low and High Racial and Ethnic Minority ACOs **eFigure 4. **Predicted Percent of Beneficiaries Receiving LDL Cholesterol Testing at Each Percentile of Out-of-Network Primary Care Stratified by Racial and Ethnic Minority and White Beneficiaries in Low and High Racial and Ethnic Minority ACOs **eFigure 5. **Predicted Percent of Beneficiaries Receiving Mammography Screening at Each Percentile of Out-of-Network Primary Care Stratified by Racial and Ethnic Minority and White Beneficiaries in Low and High Racial and Ethnic Minority ACOs **eFigure 6. **Predicted Percent of Beneficiaries with Hospitalization for CHF at Each Percentile of Out-of-Network Primary Care Stratified by Racial and Ethnic Minority and White Beneficiaries in Low and High Racial and Ethnic Minority ACOs **eFigure 7. **Predicted Percent of Beneficiaries with Hospitalization for COPD at Each Percentile of Out-of-Network Primary Care Stratified by Racial and Ethnic Minority and White Beneficiaries in Low and High Racial and Ethnic Minority ACOs **eFigure 8. **Predicted Percent of Beneficiaries with a Readmission for Any Reason at Each Percentile of Out-of-Network Primary Care Stratified by Racial and Ethnic Minority and White Beneficiaries in Low and High Racial and Ethnic Minority ACOs **eFigure 9. **Predicted Percent of Beneficiaries with an ED Visit for Any Reason at Each Percentile of Out-of-Network Primary Care Stratified by Racial and Ethnic Minority and White Beneficiaries in Low and High Racial and Ethnic Minority ACOs
