# Supplementary material for: Association Between Organizational Quality and Out-of-Network Primary Care Among Accountable Care Organizations That Care for High vs Low Proportions of Patients of Racial and Ethnic Minority Groups
Source: JAMA Health Forum. 2022 Apr 15;3(4):e220575. doi: 10.1001/jamahealthforum.2022.0575 (PMC9012967; doi:10.1001/jamahealthforum.2022.0575)
Supplement: Supplement. — eTable 1. Multilevel regression results, quantifying the percentage of residual variance in out-of-network primary care attributable to the beneficiary and ACO levels eTable 2. Full multilevel model, demonstrating independent associations between beneficiary- and ACO-level factors and out-of-network primary care eTable 3. Full multivariable logistic regression models, examining preventive services receipt and hospital utilization among beneficiaries assigned to high racial and ethnic minority ACOs at the mean level of out-of-network primary care eTable 4. Adjusted risk differences on sensitivity analyses eFigure 1. Predicted Percent of Beneficiaries Receiving All Diabetes Tests at Each Percentile of Out-of-Network Primary Care Stratified by Racial and Ethnic Minority and White Beneficiaries in Low and High Racial and Ethnic Minority ACOs eFigure 2. Predicted Percent of Beneficiaries Receiving Diabetic Retinal Examinations at Each Percentile of Out-of-Network Primary Care Stratified by Racial and Ethnic Minority and White Beneficiaries in Low and High Racial and Ethnic Minority ACOs eFigure 3. Predicted Percent of Beneficiaries Receiving Glycated Hemoglobin Testing at Each Percentile of Out-of-Network Primary Care Stratified by Racial and Ethnic Minority and White Beneficiaries in Low and High Racial and Ethnic Minority ACOs eFigure 4. Predicted Percent of Beneficiaries Receiving LDL Cholesterol Testing at Each Percentile of Out-of-Network Primary Care Stratified by Racial and Ethnic Minority and White Beneficiaries in Low and High Racial and Ethnic Minority ACOs eFigure 5. Predicted Percent of Beneficiaries Receiving Mammography Screening at Each Percentile of Out-of-Network Primary Care Stratified by Racial and Ethnic Minority and White Beneficiaries in Low and High Racial and Ethnic Minority ACOs eFigure 6. Predicted Percent of Beneficiaries with Hospitalization for CHF at Each Percentile of Out-of-Network Primary Care Stratified by Racial and Ethnic Minority and [file jamahealthforum-e220575-s001.pdf]

Supplemental Online Content

Bakre S, Moloci N, Norton EC, et al. Association between organizational quality and out-of-network primary care among accountable care organizations that care for high vs low proportions of patients of racial and ethnic minority groups. *JAMA Health Forum*. 2022;3(4):e220575. doi:10.1001/jamahealthforum.2022.0575

eTable 1. Multilevel regression results, quantifying the percentage of residual variance in out-of-network primary care attributable to the beneficiary and ACO levels
eTable 2. Full multilevel model, demonstrating independent associations between beneficiary- and ACO-level factors and out-of-network primary care
eTable 3. Full multivariable logistic regression models, examining preventive services receipt and hospital utilization among beneficiaries assigned to high racial and ethnic minority ACOs at the mean level of out-of-network primary care
eTable 4. Adjusted risk differences on sensitivity analyses
eFigure 1. Predicted Percent of Beneficiaries Receiving All Diabetes Tests at Each Percentile of Out-of-Network Primary Care Stratified by Racial and Ethnic Minority and White Beneficiaries in Low and High Racial and Ethnic Minority ACOs
eFigure 2. Predicted Percent of Beneficiaries Receiving Diabetic Retinal Examinations at Each Percentile of Out-of-Network Primary Care Stratified by Racial and Ethnic Minority and White Beneficiaries in Low and High Racial and Ethnic Minority ACOs
eFigure 3. Predicted Percent of Beneficiaries Receiving Glycated Hemoglobin Testing at Each Percentile of Out-of-Network Primary Care Stratified by Racial and Ethnic Minority and White Beneficiaries in Low and High Racial and Ethnic Minority ACOs
eFigure 4. Predicted Percent of Beneficiaries Receiving LDL Cholesterol Testing at Each Percentile of Out-of-Network Primary Care Stratified by Racial and Ethnic Minority and White Beneficiaries in Low and High Racial and Ethnic Minority ACOs
eFigure 5. Predicted Percent of Beneficiaries Receiving Mammography Screening at Each Percentile of Out-of-Network Primary Care Stratified by Racial and Ethnic Minority and White Beneficiaries in Low and High Racial and Ethnic Minority ACOs
eFigure 6. Predicted Percent of Beneficiaries with Hospitalization for CHF at Each Percentile of Out-of-Network Primary Care Stratified by Racial and Ethnic Minority and White Beneficiaries in Low and High Racial and Ethnic Minority ACOs
eFigure 7. Predicted Percent of Beneficiaries with Hospitalization for COPD at Each Percentile of Out-of-Network Primary Care Stratified by Racial and Ethnic Minority and White Beneficiaries in Low and High Racial and Ethnic Minority ACOs
eFigure 8. Predicted Percent of Beneficiaries with a Readmission for Any Reason at Each Percentile of Out-of-Network Primary Care Stratified by Racial and Ethnic Minority and White Beneficiaries in Low and High Racial and Ethnic Minority ACOs
eFigure 9. Predicted Percent of Beneficiaries with an ED Visit for Any Reason at Each Percentile of Out-of-Network Primary Care Stratified by Racial and Ethnic Minority and White Beneficiaries in Low and High Racial and Ethnic Minority ACOs

© 2022 Bakre S et al. *JAMA Health Forum.*

This supplemental material has been provided by the authors to give readers additional information about their work.

© 2022 Bakre S et al. *JAMA Health Forum.*

eTable 1. Multilevel regression results, quantifying the percentage of residual variance in out-of-network primary care attributable to the beneficiary and ACO levels

	Level (%)	
	Beneficiary	ACO
Empty model	29.4	70.6
After including beneficiary characteristics	27.1	72.9
After including beneficiary and ACO characteristics	27.4	72.6

© 2022 Bakre S et al. *JAMA Health Forum.*

eTable 2. Full multilevel model, demonstrating independent associations between beneficiary- and ACO-level factors and out-of-network primary care

Solutions for Fixed Effects																
Effect	provider_type	State Code	Sex	aco_start_year	aco_track	hrrnum	race_g2	year	Rank for Variable adr_ses_score	urban	dual_elig	Estimate	Standard Error	DF	t Value	Pr > |t|
Intercept												41.2588	38299	519	0	0.9991
provider_type	Both											-1.3804	1.5564	3.81E+06	-0.89	0.3751
provider_type	Hospital System											-1.6035	1.8651	3.81E+06	-0.86	0.3899
provider_type	Physician Group											0	.	.	.	.
aco_start_year				2012								-3.4573	2.1497	3.81E+06	-1.61	0.1078
aco_start_year				2013								-2.6844	2.1953	3.81E+06	-1.22	0.2214
aco_start_year				2014								-3.8414	2.1391	3.81E+06	-1.8	0.0725
aco_start_year				2015								-3.685	2.2877	3.81E+06	-1.61	0.1072
aco_start_year				2016								0	.	.	.	.
aco_track					1							-8.3769	0.05028	3.81E+06	-166.62	<.0001
aco_track					2							-7.2394	0.1106	3.81E+06	-65.46	<.0001
aco_track					3							0	.	.	.	.
lives_covered												6.48E-06	0	3.81E+06	Infty	<.0001

© 2022 Bakre S et al. *JAMA Health Forum.*

bene_tot_pcp_visit												-0.01238	0.001667	3.81E+06	-7.42	<.0001
STATE		1										-0.663	0.6945	3.81E+06	-0.95	0.3397
STATE		2										-1.2579	2.4091	3.81E+06	-0.52	0.6016
STATE		3										0.04314	0.6707	3.81E+06	0.06	0.9487
STATE		4										-0.528	0.6718	3.81E+06	-0.79	0.4319
STATE		5										0.1198	0.6543	3.81E+06	0.18	0.8547
STATE		6										-0.4039	0.6443	3.81E+06	-0.63	0.5307
STATE		7										1.3087	0.7241	3.81E+06	1.81	0.0707
STATE		8										0.04852	0.7127	3.81E+06	0.07	0.9457
STATE		9										-0.131	0.7162	3.81E+06	-0.18	0.8549
STATE		10										-1.1659	0.6936	3.81E+06	-1.68	0.0928
STATE		11										-0.7904	0.6785	3.81E+06	-1.16	0.244
STATE		12										-2.0324	6.8319	3.81E+06	-0.3	0.7661
STATE		13										0.1056	0.5295	3.81E+06	0.2	0.8419
STATE		14										0.5314	0.6515	3.81E+06	0.82	0.4147
STATE		15										-0.2693	0.66	3.81E+06	-0.41	0.6832
STATE		16										0.5901	0.6538	3.81E+06	0.9	0.3668

© 2022 Bakre S et al. *JAMA Health Forum.*

STATE		17										0.3987	0.6663	3.81E+06	0.6	0.5496
STATE		18										-0.2427	0.6641	3.81E+06	-0.37	0.7148
STATE		19										-0.4966	0.7661	3.81E+06	-0.65	0.5169
STATE		20										-7.0467	0.7476	3.81E+06	-9.43	<.0001
STATE		21										-0.09085	0.7026	3.81E+06	-0.13	0.8971
STATE		22										-3.5404	0.7447	3.81E+06	-4.75	<.0001
STATE		23										-0.1367	0.6696	3.81E+06	-0.2	0.8383
STATE		24										-0.1098	0.6745	3.81E+06	-0.16	0.8707
STATE		25										-0.5995	0.7013	3.81E+06	-0.85	0.3926
STATE		26										0.4598	0.6555	3.81E+06	0.7	0.483
STATE		27										-0.1645	0.2306	3.81E+06	-0.71	0.4756
STATE		28										0.797	0.6624	3.81E+06	1.2	0.2289
STATE		29										-0.1494	0.6448	3.81E+06	-0.23	0.8167
STATE		30										-0.566	0.7225	3.81E+06	-0.78	0.4334
STATE		31										-0.2162	0.6987	3.81E+06	-0.31	0.757

© 2022 Bakre S et al. *JAMA Health Forum.*

STATE		32										-0.9038	0.6966	3.81E+06	-1.3	0.1945
STATE		33										-0.1336	0.7	3.81E+06	-0.19	0.8486
STATE		34										0.2993	0.682	3.81E+06	0.44	0.6608
STATE		35										-0.6925	0.7024	3.81E+06	-0.99	0.3242
STATE		36										0.05558	0.6681	3.81E+06	0.08	0.9337
STATE		37										-0.3968	0.6864	3.81E+06	-0.58	0.5632
STATE		38										-0.2145	0.568	3.81E+06	-0.38	0.7057
STATE		39										-0.2386	0.6957	3.81E+06	-0.34	0.7316
STATE		41										4.825	0.7635	3.81E+06	6.32	<.0001
STATE		42										-0.5034	0.6914	3.81E+06	-0.73	0.4666
STATE		43										0.496	0.7198	3.81E+06	0.69	0.4908
STATE		44										-0.3232	0.6669	3.81E+06	-0.48	0.628
STATE		45										-0.492	0.687	3.81E+06	-0.72	0.4739
STATE		46										-0.1927	0.53	3.81E+06	-0.36	0.7161
STATE		47										-0.9936	0.72	3.81E+06	-1.38	0.1676
STATE		49										0.04689	0.6723	3.81E+06	0.07	0.9444

© 2022 Bakre S et al. *JAMA Health Forum.*

STATE		50										-0.2732	0.6021	3.81E+06	-0.45	0.65
STATE		51										-0.1368	0.6828	3.81E+06	-0.2	0.8412
STATE		52										-0.05326	0.6673	3.81E+06	-0.08	0.9364
STATE		53										0	.	.	.	.
hrrnum						1						-18.3331	38299	3.81E+06	0	0.9996
hrrnum						2						-18.3691	38299	3.81E+06	0	0.9996
hrrnum						5						-18.8699	38299	3.81E+06	0	0.9996
hrrnum						6						-18.6128	38299	3.81E+06	0	0.9996
hrrnum						7						-18.3562	38299	3.81E+06	0	0.9996
hrrnum						9						-18.9131	38299	3.81E+06	0	0.9996
hrrnum						10						-16.2903	38299	3.81E+06	0	0.9997
hrrnum						11						-17.651	38299	3.81E+06	0	0.9996
hrrnum						12						-17.9996	38299	3.81E+06	0	0.9996
hrrnum						14						-17.8499	38299	3.81E+06	0	0.9996

© 2022 Bakre S et al. *JAMA Health Forum.*

hrrnum						15						-20.9274	38299	3.81E+06	0	0.9996
hrrnum						16						-17.4128	38299	3.81E+06	0	0.9996
hrrnum						18						-17.6192	38299	3.81E+06	0	0.9996
hrrnum						19						-18.164	38299	3.81E+06	0	0.9996
hrrnum						21						-17.582	38299	3.81E+06	0	0.9996
hrrnum						22						-18.0998	38299	3.81E+06	0	0.9996
hrrnum						23						-18.9329	38299	3.81E+06	0	0.9996
hrrnum						25						-16.8386	38299	3.81E+06	0	0.9996
hrrnum						31						-19.5995	38299	3.81E+06	0	0.9996
hrrnum						33						-18.5147	38299	3.81E+06	0	0.9996
hrrnum						43						-17.1214	38299	3.81E+06	0	0.9996
hrrnum						56						-18.9569	38299	3.81E+06	0	0.9996
hrrnum						58						-18.2171	38299	3.81E+06	0	0.9996
hrrnum						62						-19.4544	38299	3.81E+06	0	0.9996

© 2022 Bakre S et al. *JAMA Health Forum.*

hrrnum						65						-18.9373	38299	3.81E+06	0	0.9996
hrrnum						69						-18.8447	38299	3.81E+06	0	0.9996
hrrnum						73						-18.7077	38299	3.81E+06	0	0.9996
hrrnum						77						-19.1329	38299	3.81E+06	0	0.9996
hrrnum						78						-17.3038	38299	3.81E+06	0	0.9996
hrrnum						79						-18.8774	38299	3.81E+06	0	0.9996
hrrnum						80						-18.6145	38299	3.81E+06	0	0.9996
hrrnum						81						-18.4054	38299	3.81E+06	0	0.9996
hrrnum						82						-18.6047	38299	3.81E+06	0	0.9996
hrrnum						83						-18.3969	38299	3.81E+06	0	0.9996
hrrnum						85						-18.8264	38299	3.81E+06	0	0.9996
hrrnum						86						-19.0208	38299	3.81E+06	0	0.9996
hrrnum						87						-18.192	38299	3.81E+06	0	0.9996
hrrnum						89						-18.6142	38299	3.81E+06	0	0.9996

© 2022 Bakre S et al. *JAMA Health Forum.*

hrrnum						91						-19.8084	38299	3.81E+06	0	0.9996
hrrnum						96						-18.9431	38299	3.81E+06	0	0.9996
hrrnum						101						-18.1719	38299	3.81E+06	0	0.9996
hrrnum						102						-18.2236	38299	3.81E+06	0	0.9996
hrrnum						103						-17.656	38299	3.81E+06	0	0.9996
hrrnum						104						-17.6666	38299	3.81E+06	0	0.9996
hrrnum						105						-17.5866	38299	3.81E+06	0	0.9996
hrrnum						106						-17.6273	38299	3.81E+06	0	0.9996
hrrnum						107						-17.0211	38299	3.81E+06	0	0.9996
hrrnum						109						-21.837	38299	3.81E+06	0	0.9995
hrrnum						110						-19.9035	38299	3.81E+06	0	0.9996
hrrnum						111						-19.6649	38299	3.81E+06	0	0.9996
hrrnum						112						-19.2702	38299	3.81E+06	0	0.9996
hrrnum						113						-18.6937	38299	3.81E+06	0	0.9996

© 2022 Bakre S et al. *JAMA Health Forum.*

hrrnum						115						-16.845	38299	3.81E+06	0	0.9996
hrrnum						116						-17.9207	38299	3.81E+06	0	0.9996
hrrnum						118						-17.0094	38299	3.81E+06	0	0.9996
hrrnum						119						-16.2356	38299	3.81E+06	0	0.9997
hrrnum						120						-17.7154	38299	3.81E+06	0	0.9996
hrrnum						122						-17.926	38299	3.81E+06	0	0.9996
hrrnum						123						-17.6982	38299	3.81E+06	0	0.9996
hrrnum						124						-17.9215	38299	3.81E+06	0	0.9996
hrrnum						127						-16.3175	38299	3.81E+06	0	0.9997
hrrnum						129						-18.4977	38299	3.81E+06	0	0.9996
hrrnum						130						-19.2401	38299	3.81E+06	0	0.9996
hrrnum						131						-18.0236	38299	3.81E+06	0	0.9996
hrrnum						133						-17.8784	38299	3.81E+06	0	0.9996
hrrnum						134						-17.6366	38299	3.81E+06	0	0.9996

© 2022 Bakre S et al. *JAMA Health Forum.*

hrrnum						137						-16.89	38299	3.81E+06	0	0.9996
hrrnum						139						-17.8573	38299	3.81E+06	0	0.9996
hrrnum						140						-18.5781	38299	3.81E+06	0	0.9996
hrrnum						141						-17.8024	38299	3.81E+06	0	0.9996
hrrnum						142						-18.0138	38299	3.81E+06	0	0.9996
hrrnum						144						-17.6788	38299	3.81E+06	0	0.9996
hrrnum						145						-18.2808	38299	3.81E+06	0	0.9996
hrrnum						146						-17.8333	38299	3.81E+06	0	0.9996
hrrnum						147						-17.8888	38299	3.81E+06	0	0.9996
hrrnum						148						-17.3366	38299	3.81E+06	0	0.9996
hrrnum						149						-18.3867	38299	3.81E+06	0	0.9996
hrrnum						150						-16.4115	38299	3.81E+06	0	0.9997
hrrnum						151						-18.5326	38299	3.81E+06	0	0.9996
hrrnum						152						-17.9452	38299	3.81E+06	0	0.9996

© 2022 Bakre S et al. *JAMA Health Forum.*

hrrnum						154						-18.2412	38299	3.81E+06	0	0.9996
hrrnum						155						-18.3169	38299	3.81E+06	0	0.9996
hrrnum						156						-18.2269	38299	3.81E+06	0	0.9996
hrrnum						158						-18.3143	38299	3.81E+06	0	0.9996
hrrnum						161						-18.2947	38299	3.81E+06	0	0.9996
hrrnum						163						-18.6237	38299	3.81E+06	0	0.9996
hrrnum						164						-18.7036	38299	3.81E+06	0	0.9996
hrrnum						166						-18.467	38299	3.81E+06	0	0.9996
hrrnum						170						-17.0391	38299	3.81E+06	0	0.9996
hrrnum						171						-18.2726	38299	3.81E+06	0	0.9996
hrrnum						172						-17.346	38299	3.81E+06	0	0.9996
hrrnum						173						-18.4324	38299	3.81E+06	0	0.9996
hrrnum						175						-18.2722	38299	3.81E+06	0	0.9996
hrrnum						179						-17.822	38299	3.81E+06	0	0.9996

© 2022 Bakre S et al. *JAMA Health Forum.*

hrrnum						180						-18.5724	38299	3.81E+06	0	0.9996
hrrnum						181						-18.3647	38299	3.81E+06	0	0.9996
hrrnum						183						-17.9593	38299	3.81E+06	0	0.9996
hrrnum						184						-18.4992	38299	3.81E+06	0	0.9996
hrrnum						185						-18.2607	38299	3.81E+06	0	0.9996
hrrnum						186						-18.3472	38299	3.81E+06	0	0.9996
hrrnum						187						-18.2323	38299	3.81E+06	0	0.9996
hrrnum						188						-17.7026	38299	3.81E+06	0	0.9996
hrrnum						190						-16.7768	38299	3.81E+06	0	0.9997
hrrnum						191						-16.9112	38299	3.81E+06	0	0.9996
hrrnum						192						-16.0744	38299	3.81E+06	0	0.9997
hrrnum						193						-15.7156	38299	3.81E+06	0	0.9997
hrrnum						194						-16.5999	38299	3.81E+06	0	0.9997
hrrnum						195						-17.0138	38299	3.81E+06	0	0.9996

© 2022 Bakre S et al. *JAMA Health Forum.*

hrrnum						196							-18.2393	38299	3.81E+06	0	0.9996
hrrnum						197							-16.9556	38299	3.81E+06	0	0.9996
hrrnum						200							-18.9455	38299	3.81E+06	0	0.9996
hrrnum						201							-18.6604	38299	3.81E+06	0	0.9996
hrrnum						203							-17.7057	38299	3.81E+06	0	0.9996
hrrnum						204							-18.7432	38299	3.81E+06	0	0.9996
hrrnum						205							-18.2107	38299	3.81E+06	0	0.9996
hrrnum						207							-17.8647	38299	3.81E+06	0	0.9996
hrrnum						208							-18.6552	38299	3.81E+06	0	0.9996
hrrnum						209							-18.2271	38299	3.81E+06	0	0.9996
hrrnum						210							-18.7595	38299	3.81E+06	0	0.9996
hrrnum						212							-19.0249	38299	3.81E+06	0	0.9996
hrrnum						213							-18.6334	38299	3.81E+06	0	0.9996
hrrnum						214							-18.048	38299	3.81E+06	0	0.9996

© 2022 Bakre S et al. *JAMA Health Forum.*

hrrnum						216						-18.539	38299	3.81E+06	0	0.9996
hrrnum						217						-18.9054	38299	3.81E+06	0	0.9996
hrrnum						218						-19.0811	38299	3.81E+06	0	0.9996
hrrnum						219						-17.5893	38299	3.81E+06	0	0.9996
hrrnum						220						-18.4292	38299	3.81E+06	0	0.9996
hrrnum						221						-13.2439	38299	3.81E+06	0	0.9997
hrrnum						222						-12.4743	38299	3.81E+06	0	0.9997
hrrnum						223						-18.782	38299	3.81E+06	0	0.9996
hrrnum						225						-19.0933	38299	3.81E+06	0	0.9996
hrrnum						226						-18.7441	38299	3.81E+06	0	0.9996
hrrnum						227						-14.4431	38299	3.81E+06	0	0.9997
hrrnum						230						-18.2845	38299	3.81E+06	0	0.9996
hrrnum						231						-12.0428	38299	3.81E+06	0	0.9997
hrrnum						232						-18.3358	38299	3.81E+06	0	0.9996

© 2022 Bakre S et al. *JAMA Health Forum.*

hrrnum						233						-18.1666	38299	3.81E+06	0	0.9996
hrrnum						234						-18.2598	38299	3.81E+06	0	0.9996
hrrnum						235						-18.38	38299	3.81E+06	0	0.9996
hrrnum						236						-18.1717	38299	3.81E+06	0	0.9996
hrrnum						238						-18.1279	38299	3.81E+06	0	0.9996
hrrnum						239						-18.4727	38299	3.81E+06	0	0.9996
hrrnum						240						-18.3873	38299	3.81E+06	0	0.9996
hrrnum						242						-18.5555	38299	3.81E+06	0	0.9996
hrrnum						243						-18.3952	38299	3.81E+06	0	0.9996
hrrnum						244						-18.2555	38299	3.81E+06	0	0.9996
hrrnum						245						-18.2618	38299	3.81E+06	0	0.9996
hrrnum						246						-18.5136	38299	3.81E+06	0	0.9996
hrrnum						248						-18.3055	38299	3.81E+06	0	0.9996
hrrnum						249						-18.2469	38299	3.81E+06	0	0.9996

© 2022 Bakre S et al. *JAMA Health Forum.*

hrrnum						250						-17.9138	38299	3.81E+06	0	0.9996
hrrnum						251						-17.9993	38299	3.81E+06	0	0.9996
hrrnum						253						-17.0769	38299	3.81E+06	0	0.9996
hrrnum						254						-18.2336	38299	3.81E+06	0	0.9996
hrrnum						256						-18.1966	38299	3.81E+06	0	0.9996
hrrnum						257						-18.8449	38299	3.81E+06	0	0.9996
hrrnum						258						-18.7354	38299	3.81E+06	0	0.9996
hrrnum						259						-18.702	38299	3.81E+06	0	0.9996
hrrnum						260						-15.9541	38299	3.81E+06	0	0.9997
hrrnum						261						-19.185	38299	3.81E+06	0	0.9996
hrrnum						262						-19.5648	38299	3.81E+06	0	0.9996
hrrnum						263						-19.8223	38299	3.81E+06	0	0.9996
hrrnum						264						-18.354	38299	3.81E+06	0	0.9996
hrrnum						267						-18.1933	38299	3.81E+06	0	0.9996

© 2022 Bakre S et al. *JAMA Health Forum.*

hrrnum						268						-18.3279	38299	3.81E+06	0	0.9996
hrrnum						270						-18.8743	38299	3.81E+06	0	0.9996
hrrnum						273						-18.4275	38299	3.81E+06	0	0.9996
hrrnum						274						-18.7075	38299	3.81E+06	0	0.9996
hrrnum						275						-18.6062	38299	3.81E+06	0	0.9996
hrrnum						276						-18.7894	38299	3.81E+06	0	0.9996
hrrnum						277						-17.725	38299	3.81E+06	0	0.9996
hrrnum						278						-17.0439	38299	3.81E+06	0	0.9996
hrrnum						279						-18.6187	38299	3.81E+06	0	0.9996
hrrnum						280						-18.3859	38299	3.81E+06	0	0.9996
hrrnum						281						-18.7605	38299	3.81E+06	0	0.9996
hrrnum						282						-17.3278	38299	3.81E+06	0	0.9996
hrrnum						283						-18.7224	38299	3.81E+06	0	0.9996
hrrnum						284						-18.787	38299	3.81E+06	0	0.9996

© 2022 Bakre S et al. *JAMA Health Forum.*

hrrnum						285						-18.8272	38299	3.81E+06	0	0.9996
hrrnum						288						-18.8521	38299	3.81E+06	0	0.9996
hrrnum						289						-19.0009	38299	3.81E+06	0	0.9996
hrrnum						291						-18.9693	38299	3.81E+06	0	0.9996
hrrnum						292						-18.517	38299	3.81E+06	0	0.9996
hrrnum						293						-17.0902	38299	3.81E+06	0	0.9996
hrrnum						295						-18.9776	38299	3.81E+06	0	0.9996
hrrnum						296						-18.9701	38299	3.81E+06	0	0.9996
hrrnum						297						-18.8525	38299	3.81E+06	0	0.9996
hrrnum						299						-18.8259	38299	3.81E+06	0	0.9996
hrrnum						300						-19.2583	38299	3.81E+06	0	0.9996
hrrnum						301						-18.6659	38299	3.81E+06	0	0.9996
hrrnum						303						-18.7819	38299	3.81E+06	0	0.9996
hrrnum						304						-18.6904	38299	3.81E+06	0	0.9996

© 2022 Bakre S et al. *JAMA Health Forum.*

hrrnum						307						-19.0152	38299	3.81E+06	0	0.9996
hrrnum						308						-18.5073	38299	3.81E+06	0	0.9996
hrrnum						309						-18.2512	38299	3.81E+06	0	0.9996
hrrnum						311						-17.5181	38299	3.81E+06	0	0.9996
hrrnum						312						-18.6509	38299	3.81E+06	0	0.9996
hrrnum						313						-19.093	38299	3.81E+06	0	0.9996
hrrnum						314						-19.8673	38299	3.81E+06	0	0.9996
hrrnum						315						-18.7817	38299	3.81E+06	0	0.9996
hrrnum						318						-19.1836	38299	3.81E+06	0	0.9996
hrrnum						319						-20.1961	38299	3.81E+06	0	0.9996
hrrnum						320						-19.2833	38299	3.81E+06	0	0.9996
hrrnum						321						-18.3821	38299	3.81E+06	0	0.9996
hrrnum						322						-17.3205	38299	3.81E+06	0	0.9996
hrrnum						323						-17.9442	38299	3.81E+06	0	0.9996

© 2022 Bakre S et al. *JAMA Health Forum.*

hrrnum						324						-19.1062	38299	3.81E+06	0	0.9996
hrrnum						325						-18.2202	38299	3.81E+06	0	0.9996
hrrnum						326						-18.6764	38299	3.81E+06	0	0.9996
hrrnum						327						-17.5684	38299	3.81E+06	0	0.9996
hrrnum						328						-18.6541	38299	3.81E+06	0	0.9996
hrrnum						329						-18.4241	38299	3.81E+06	0	0.9996
hrrnum						330						-18.5053	38299	3.81E+06	0	0.9996
hrrnum						331						-18.9935	38299	3.81E+06	0	0.9996
hrrnum						332						-18.2183	38299	3.81E+06	0	0.9996
hrrnum						334						-18.803	38299	3.81E+06	0	0.9996
hrrnum						335						-19.4355	38299	3.81E+06	0	0.9996
hrrnum						336						-17.9113	38299	3.81E+06	0	0.9996
hrrnum						339						-17.7957	38299	3.81E+06	0	0.9996
hrrnum						340						-17.7944	38299	3.81E+06	0	0.9996

© 2022 Bakre S et al. *JAMA Health Forum.*

hrrnum						341						-18.0954	38299	3.81E+06	0	0.9996
hrrnum						342						-18.0525	38299	3.81E+06	0	0.9996
hrrnum						343						-18.1904	38299	3.81E+06	0	0.9996
hrrnum						344						-18.2025	38299	3.81E+06	0	0.9996
hrrnum						345						-18.1771	38299	3.81E+06	0	0.9996
hrrnum						346						-18.818	38299	3.81E+06	0	0.9996
hrrnum						347						-18.7268	38299	3.81E+06	0	0.9996
hrrnum						350						-18.3863	38299	3.81E+06	0	0.9996
hrrnum						351						-19.0065	38299	3.81E+06	0	0.9996
hrrnum						352						-18.5162	38299	3.81E+06	0	0.9996
hrrnum						354						-18.4698	38299	3.81E+06	0	0.9996
hrrnum						355						-18.2623	38299	3.81E+06	0	0.9996
hrrnum						356						-18.942	38299	3.81E+06	0	0.9996
hrrnum						357						-18.779	38299	3.81E+06	0	0.9996

© 2022 Bakre S et al. *JAMA Health Forum.*

hrrnum						358						-18.3475	38299	3.81E+06	0	0.9996
hrrnum						359						-18.9221	38299	3.81E+06	0	0.9996
hrrnum						360						-18.3298	38299	3.81E+06	0	0.9996
hrrnum						362						-18.8872	38299	3.81E+06	0	0.9996
hrrnum						363						-19.418	38299	3.81E+06	0	0.9996
hrrnum						364						-25.8535	38299	3.81E+06	0	0.9995
hrrnum						365						-18.3513	38299	3.81E+06	0	0.9996
hrrnum						366						-18.2527	38299	3.81E+06	0	0.9996
hrrnum						367						-19.2035	38299	3.81E+06	0	0.9996
hrrnum						368						-18.3604	38299	3.81E+06	0	0.9996
hrrnum						369						-18.2258	38299	3.81E+06	0	0.9996
hrrnum						370						-18.7439	38299	3.81E+06	0	0.9996
hrrnum						371						-17.898	38299	3.81E+06	0	0.9996
hrrnum						373						-18.2688	38299	3.81E+06	0	0.9996

© 2022 Bakre S et al. *JAMA Health Forum.*

hrrnum						374						-18.6988	38299	3.81E+06	0	0.9996
hrrnum						375						-17.4485	38299	3.81E+06	0	0.9996
hrrnum						376						-17.7761	38299	3.81E+06	0	0.9996
hrrnum						377						-18.3738	38299	3.81E+06	0	0.9996
hrrnum						379						-18.6514	38299	3.81E+06	0	0.9996
hrrnum						380						-18.5122	38299	3.81E+06	0	0.9996
hrrnum						382						-18.1977	38299	3.81E+06	0	0.9996
hrrnum						383						-18.235	38299	3.81E+06	0	0.9996
hrrnum						385						-17.468	38299	3.81E+06	0	0.9996
hrrnum						386						-18.3759	38299	3.81E+06	0	0.9996
hrrnum						388						-17.8809	38299	3.81E+06	0	0.9996
hrrnum						390						-17.7227	38299	3.81E+06	0	0.9996
hrrnum						391						-18.0748	38299	3.81E+06	0	0.9996
hrrnum						393						-17.1085	38299	3.81E+06	0	0.9996

© 2022 Bakre S et al. *JAMA Health Forum.*

hrrnum						394						-18.0492	38299	3.81E+06	0	0.9996
hrrnum						396						-17.1306	38299	3.81E+06	0	0.9996
hrrnum						397						-17.5739	38299	3.81E+06	0	0.9996
hrrnum						399						-21.4178	38299	3.81E+06	0	0.9996
hrrnum						400						-16.2704	38299	3.81E+06	0	0.9997
hrrnum						402						-17.6466	38299	3.81E+06	0	0.9996
hrrnum						406						-15.9319	38299	3.81E+06	0	0.9997
hrrnum						411						-16.3896	38299	3.81E+06	0	0.9997
hrrnum						412						-18.1373	38299	3.81E+06	0	0.9996
hrrnum						413						-17.6456	38299	3.81E+06	0	0.9996
hrrnum						416						-18.7163	38299	3.81E+06	0	0.9996
hrrnum						417						-18.1528	38299	3.81E+06	0	0.9996
hrrnum						418						-18.262	38299	3.81E+06	0	0.9996
hrrnum						420						-17.2759	38299	3.81E+06	0	0.9996

© 2022 Bakre S et al. *JAMA Health Forum.*

hrrnum						421						-18.7681	38299	3.81E+06	0	0.9996
hrrnum						422						-18.6431	38299	3.81E+06	0	0.9996
hrrnum						423						-18.6628	38299	3.81E+06	0	0.9996
hrrnum						424						-18.1736	38299	3.81E+06	0	0.9996
hrrnum						426						-18.8534	38299	3.81E+06	0	0.9996
hrrnum						427						-18.7317	38299	3.81E+06	0	0.9996
hrrnum						428						-18.7287	38299	3.81E+06	0	0.9996
hrrnum						429						-18.9907	38299	3.81E+06	0	0.9996
hrrnum						430						-18.9567	38299	3.81E+06	0	0.9996
hrrnum						431						-18.9901	38299	3.81E+06	0	0.9996
hrrnum						432						-18.6595	38299	3.81E+06	0	0.9996
hrrnum						435						-19.2067	38299	3.81E+06	0	0.9996
hrrnum						437						-17.6256	38299	3.81E+06	0	0.9996
hrrnum						438						-17.5816	38299	3.81E+06	0	0.9996

© 2022 Bakre S et al. *JAMA Health Forum.*

hrrnum						439						-17.8372	38299	3.81E+06	0	0.9996
hrrnum						440						-18.8477	38299	3.81E+06	0	0.9996
hrrnum						441						-18.0387	38299	3.81E+06	0	0.9996
hrrnum						442						-17.8518	38299	3.81E+06	0	0.9996
hrrnum						443						-18.1161	38299	3.81E+06	0	0.9996
hrrnum						444						-18.7345	38299	3.81E+06	0	0.9996
hrrnum						445						-19.0451	38299	3.81E+06	0	0.9996
hrrnum						446						-17.9269	38299	3.81E+06	0	0.9996
hrrnum						447						-18.4783	38299	3.81E+06	0	0.9996
hrrnum						448						-17.4363	38299	3.81E+06	0	0.9996
hrrnum						449						-18.084	38299	3.81E+06	0	0.9996
hrrnum						450						-17.9972	38299	3.81E+06	0	0.9996
hrrnum						451						-18.1183	38299	3.81E+06	0	0.9996
hrrnum						452						-17.728	38299	3.81E+06	0	0.9996

© 2022 Bakre S et al. *JAMA Health Forum.*

hrrnum						456						-17.9475	38299	3.81E+06	0	0.9996
hrrnum						457						-18.6059	38299	3.81E+06	0	0.9996
hrrnum						999						-17.6383	38299	3.81E+06	0	0.9996
AGE												-0.00097	0.000684	3.81E+06	-1.42	0.1546
SEX			1									-0.00964	0.01029	3.81E+06	-0.94	0.3488
SEX			2									0	.	.	.	.
race_g2							0					0.03263	0.01676	3.81E+06	1.95	0.0516
race_g2							1					0	.	.	.	.
year								2013				-0.9221	0.01841	3.81E+06	-50.08	<.0001
year								2014				-0.4148	0.01566	3.81E+06	-26.48	<.0001
year								2015				7.5172	0.01917	3.81E+06	392.18	<.0001
year								2016				0	.	.	.	.
ses_rank_t3									0			0.004591	0.01495	3.81E+06	0.31	0.7587
ses_rank_t3									1			-0.00003	0.01347	3.81E+06	0	0.9983
ses_rank_t3									2			0	.	.	.	.
urban										0		-0.2348	0.04137	3.81E+06	-5.68	<.0001
urban										1		0	.	.	.	.

© 2022 Bakre S et al. *JAMA Health Forum.*

dual_elig											0	-0.115	0.01801	3.81E+06	-6.38	<.0001
dual_elig											1	0	.	.	.	.
HCC1												-0.1234	0.1628	3.81E+06	-0.76	0.4483
HCC2												0.09376	0.04049	3.81E+06	2.32	0.0206
HCC6												0.03289	0.09664	3.81E+06	0.34	0.7336
HCC8												-0.00656	0.04699	3.81E+06	-0.14	0.889
HCC9												0.02063	0.04182	3.81E+06	0.49	0.6218
HCC10												-0.03998	0.03895	3.81E+06	-1.03	0.3047
HCC11												-0.03367	0.03181	3.81E+06	-1.06	0.2898
HCC12												-0.00789	0.01874	3.81E+06	-0.42	0.6739
HCC17												0.07167	0.0936	3.81E+06	0.77	0.4438
HCC18												0.02992	0.01726	3.81E+06	1.73	0.0831
HCC19												-0.01702	0.0134	3.81E+06	-1.27	0.2039
HCC21												-0.05032	0.05507	3.81E+06	-0.91	0.3609
HCC22												0.01774	0.03268	3.81E+06	0.54	0.5872
HCC23												0.02427	0.02791	3.81E+06	0.87	0.3846
HCC27												0.1362	0.09754	3.81E+06	1.4	0.1625

© 2022 Bakre S et al. *JAMA Health Forum.*

HCC28												-0.0668	0.07977	3.81E+06	-0.84	0.4024
HCC29												0.08153	0.08868	3.81E+06	0.92	0.3579
HCC33												0.05644	0.04372	3.81E+06	1.29	0.1967
HCC34												0.04777	0.1241	3.81E+06	0.38	0.7003
HCC35												0.0299	0.0523	3.81E+06	0.57	0.5675
HCC39												-0.01619	0.05862	3.81E+06	-0.28	0.7825
HCC40												-0.0198	0.02176	3.81E+06	-0.91	0.363
HCC46												0.07792	0.06971	3.81E+06	1.12	0.2637
HCC47												-0.03975	0.04562	3.81E+06	-0.87	0.3836
HCC48												0.01292	0.02552	3.81E+06	0.51	0.6126
HCC54												-0.3607	0.1488	3.81E+06	-2.42	0.0154
HCC55												0.22	0.09358	3.81E+06	2.35	0.0187
HCC57												0.1147	0.08676	3.81E+06	1.32	0.1863
HCC58												-0.06484	0.02647	3.81E+06	-2.45	0.0143
HCC70												-0.3044	0.1828	3.81E+06	-1.67	0.0959
HCC71												0.1277	0.1679	3.81E+06	0.76	0.4468
HCC72												0.02785	0.06651	3.81E+06	0.42	0.6754

© 2022 Bakre S et al. *JAMA Health Forum.*

HCC73												0.3039	0.2475	3.81E+06	1.23	0.2196
HCC74												-0.06	0.2058	3.81E+06	-0.29	0.7706
HCC75												-0.1333	0.04764	3.81E+06	-2.8	0.0051
HCC76												-0.1378	0.2919	3.81E+06	-0.47	0.6368
HCC77												0.1155	0.09033	3.81E+06	1.28	0.201
HCC78												0.02454	0.04153	3.81E+06	0.59	0.5547
HCC79												0.0176	0.03779	3.81E+06	0.47	0.6413
HCC80												0.1045	0.1267	3.81E+06	0.82	0.4095
HCC82												-0.1528	0.1337	3.81E+06	-1.14	0.2532
HCC83												-0.3604	0.3444	3.81E+06	-1.05	0.2953
HCC84												0.06262	0.03549	3.81E+06	1.76	0.0777
HCC85												0.01451	0.0176	3.81E+06	0.82	0.4098
HCC86												-0.0249	0.05015	3.81E+06	-0.5	0.6195
HCC87												0.006795	0.039	3.81E+06	0.17	0.8617
HCC88												-0.0168	0.03483	3.81E+06	-0.48	0.6296
HCC96												0.01152	0.01494	3.81E+06	0.77	0.4404
HCC99												-0.07061	0.07569	3.81E+06	-0.93	0.3509

© 2022 Bakre S et al. *JAMA Health Forum.*

HCC100												0.01231	0.02847	3.81E+06	0.43	0.6654
HCC103												-0.07004	0.05467	3.81E+06	-1.28	0.2002
HCC104												-0.3352	0.1609	3.81E+06	-2.08	0.0372
HCC106												-0.009	0.0812	3.81E+06	-0.11	0.9117
HCC107												0.0681	0.03462	3.81E+06	1.97	0.0492
HCC108												0.02309	0.01481	3.81E+06	1.56	0.119
HCC110												0.01363	0.4159	3.81E+06	0.03	0.9739
HCC111												0.01746	0.01605	3.81E+06	1.09	0.2766
HCC112												0.01477	0.04387	3.81E+06	0.34	0.7363
HCC114												-0.01473	0.06408	3.81E+06	-0.23	0.8182
HCC115												0.1346	0.1019	3.81E+06	1.32	0.1866
HCC122												-0.01331	0.06478	3.81E+06	-0.21	0.8372
HCC124												-0.03125	0.03565	3.81E+06	-0.88	0.3807
HCC134												-0.04702	0.1803	3.81E+06	-0.26	0.7943
HCC135												-0.00284	0.02993	3.81E+06	-0.1	0.9243
HCC136												0.047	0.07943	3.81E+06	0.59	0.554
HCC137												0.05448	0.05632	3.81E+06	0.97	0.3334

© 2022 Bakre S et al. *JAMA Health Forum.*

HCC157												0.1614	0.2008	3.81E+06	0.8	0.4214
HCC158												0.01575	0.1192	3.81E+06	0.13	0.8949
HCC161												-0.02828	0.03594	3.81E+06	-0.79	0.4314
HCC162												0.2517	0.4833	3.81E+06	0.52	0.6025
HCC166												-0.06963	0.4777	3.81E+06	-0.15	0.8841
HCC167												0.09501	0.07566	3.81E+06	1.26	0.2092
HCC169												-0.0235	0.04198	3.81E+06	-0.56	0.5757
HCC170												-0.00019	0.0448	3.81E+06	0	0.9966
HCC173												0.03125	0.09755	3.81E+06	0.32	0.7487
HCC176												0.04973	0.04336	3.81E+06	1.15	0.2515
HCC186												0.08047	0.1184	3.81E+06	0.68	0.4966
HCC188												0.02662	0.06581	3.81E+06	0.4	0.6858
HCC189												-0.1191	0.1187	3.81E+06	-1	0.3158

© 2022 Bakre S et al. *JAMA Health Forum.*

eTable 3. Full multivariable logistic regression models, examining preventive services receipt and hospital utilization among beneficiaries assigned to high racial and ethnic minority ACOs at the mean level of out-of-network primary care

	All Diabetes Tests	Eye Exams	HBA1c	LDL Screening	CHF	COPD/Asthma	Mammography	All-Cause Readmissions	ED Visit
	b/se	b/se	b/se	b/se	b/se	b/se	b/se	b/se	b/se
main									
Percentage of visits with a primary care physician provided by a TIN not in the	-0.000	-0.001*	0.002***	0.000	0.000	0.002**	0.000	0.001*	0.001***
	0.0003	0.0003	0.0005	0.0004	0.0004	0.0005	0.0003	0.0003	0.0001
high_minority_aco=0	0.000	0.000	0.000	0.000	0.000	0.000	0.000	0.000	0.000
	.	.	.	.	.	.	.	.	.
high_minority_aco=1	-0.080***	-0.069***	-0.123***	-0.086***	-0.017	-0.038	-0.077***	0.036*	-0.082***
	0.0105	0.0106	0.0173	0.0153	0.0213	0.0246	0.0140	0.0153	0.0051
high_minority_aco=0 # Percentage of visits with a primary care physician provided by a TIN not in the	0.000	0.000	0.000	0.000	0.000	0.000	0.000	0.000	0.000
	.	.	.	.	.	.	.	.	.
high_minority_aco=1 # Percentage of visits with a primary care physician provided by a TIN not in the	-0.002***	-0.001***	0.000	0.002**	0.006***	0.003**	-0.001**	0.002**	0.008***
	0.0004	0.0004	0.0007	0.0006	0.0008	0.0009	0.0006	0.0006	0.0002
Age at End of Reference Year	0.034***	0.046***	-0.010***	0.006***	0.058***	0.004***	0.047***	0.014***	0.039***
	0.0010	0.0010	0.0016	0.0014	0.0007	0.0008	0.0031	0.0005	0.0002
Sex=1	0.000	0.000	0.000	0.000	0.000	0.000	0.000	0.000	0.000
	.	.	.	.	.	.	.	.	.
Sex=2	0.216***	0.261***	0.134***	0.105***	-0.095***	0.103***	0.000	-0.071***	0.054***
	0.0054	0.0055	0.0091	0.0077	0.0106	0.0125	.	0.0075	0.0025
race_g2=0	0.000	0.000	0.000	0.000	0.000	0.000	0.000	0.000	0.000
	.	.	.	.	.	.	.	.	.
race_g2=1	0.018*	-0.005	0.096***	0.157***	-0.147***	0.094***	-0.094***	-0.027*	-0.016***
	0.0073	0.0074	0.0121	0.0103	0.0168	0.0207	0.0101	0.0122	0.0040
year=2013	0.000	0.000	0.000	0.000	0.000	0.000	0.000	0.000	0.000
	.	.	.	.	.	.	.	.	.
year=2014	-0.036***	-0.032***	0.024	-0.050***	0.010	-0.111***	0.621***	-0.014	0.015***

© 2022 Bakre S et al. *JAMA Health Forum.*

	0.0093	0.0094	0.0152	0.0139	0.0172	0.0202	0.0113	0.0125	0.0041
year=2015	-0.006	0.023*	0.083***	-0.158***	0.044*	-0.098***	1.015***	0.029*	0.063***
	0.0100	0.0102	0.0166	0.0147	0.0189	0.0221	0.0126	0.0135	0.0045
year=2016	-0.049***	-0.025*	0.197***	-0.177***	-0.237***	-0.197***	0.609***	0.035**	0.036***
	0.0095	0.0097	0.0159	0.0140	0.0183	0.0211	0.0116	0.0130	0.0043
Both	0.000	0.000	0.000	0.000	0.000	0.000	0.000	0.000	0.000
	.	.	.	.	.	.	.	.	.
Hospital System	0.069***	0.069***	0.142***	0.023	0.039*	0.006	0.149***	-0.006	-0.004
	0.0089	0.0090	0.0149	0.0124	0.0170	0.0201	0.0112	0.0122	0.0039
Physician Group	0.025**	-0.010	0.105***	0.124***	-0.087***	-0.052**	0.024*	-0.009	-0.090***
	0.0077	0.0078	0.0129	0.0112	0.0150	0.0174	0.0100	0.0106	0.0035
aco_start_year=2012	0.000	0.000	0.000	0.000	0.000	0.000	0.000	0.000	0.000
	.	.	.	.	.	.	.	.	.
aco_start_year=2013	0.007	0.040***	-0.073***	-0.120***	0.014	0.029	-0.034**	0.017	0.047***
	0.0089	0.0091	0.0150	0.0131	0.0172	0.0199	0.0115	0.0121	0.0041
aco_start_year=2014	-0.004	-0.007	0.029	-0.073***	0.028	0.084***	-0.001	-0.013	0.037***
	0.0098	0.0099	0.0164	0.0142	0.0194	0.0227	0.0128	0.0137	0.0045
aco_start_year=2015	-0.056***	-0.035**	-0.012	-0.108***	-0.006	0.005	-0.037**	-0.008	0.083***
	0.0109	0.0111	0.0184	0.0152	0.0210	0.0246	0.0143	0.0149	0.0049
aco_start_year=2016	-0.005	-0.000	-0.031	-0.032	0.098***	0.175***	0.031	0.125***	0.105***
	0.0150	0.0152	0.0256	0.0209	0.0293	0.0328	0.0192	0.0200	0.0067
aco_track=1	0.000	0.000	0.000	0.000	0.000	0.000	0.000	0.000	0.000
	.	.	.	.	.	.	.	.	.
aco_track=2	0.080**	0.025	0.052	0.188***	-0.010	-0.015	0.082*	0.002	-0.091***
	0.0301	0.0307	0.0531	0.0481	0.0612	0.0742	0.0389	0.0463	0.0143
aco_track=3	-0.087***	-0.020	-0.412***	-0.060	-0.011	-0.094	0.301***	0.029	-0.009
	0.0234	0.0240	0.0368	0.0324	0.0433	0.0504	0.0342	0.0308	0.0099
Rank for Variable adr_ses_score=0	0.000	0.000	0.000	0.000	0.000	0.000	0.000	0.000	0.000
	.	.	.	.	.	.	.	.	.
Rank for Variable adr_ses_score=1	0.134***	0.139***	0.079***	0.073***	-0.101***	-0.142***	0.106***	-0.078***	-0.102***
	0.0067	0.0068	0.0112	0.0094	0.0128	0.0148	0.0087	0.0092	0.0031
Rank for Variable adr_ses_score=2	0.268***	0.298***	0.169***	0.145***	-0.183***	-0.268***	0.276***	-0.132***	-0.169***
	0.0078	0.0079	0.0131	0.0112	0.0147	0.0178	0.0098	0.0105	0.0034
lives_covered	0.000***	0.000***	0.000***	0.000***	0.000	0.000	0.000***	-0.000	0.000**
	0.0000	0.0000	0.0000	0.0000	0.0000	0.0000	0.0000	0.0000	0.0000
bene_tot_pcp_visit	0.061***	0.051***	0.130***	0.103***	0.056***	0.070***	0.071***	0.007***	0.103***
	0.0009	0.0010	0.0022	0.0018	0.0011	0.0012	0.0016	0.0009	0.0004
urban=0	0.000	0.000	0.000	0.000	0.000	0.000	0.000	0.000	0.000

© 2022 Bakre S et al. *JAMA Health Forum.*

	.	.	.	.	.	.	.	.	.
urban=1	0.045*	0.036	-0.088*	0.057*	-0.058	0.026	0.011	-0.153***	-0.024*
	0.0220	0.0221	0.0371	0.0285	0.0392	0.0454	0.0280	0.0270	0.0094
dual_elig=0	0.000	0.000	0.000	0.000	0.000	0.000	0.000	0.000	0.000
	.	.	.	.	.	.	.	.	.
dual_elig=1	-0.323***	-0.283***	-0.182***	-0.249***	0.204***	0.457***	-0.647***	0.195***	0.382***
	0.0085	0.0086	0.0135	0.0113	0.0152	0.0164	0.0111	0.0105	0.0041
hrrnum=1	0.000	0.000	0.000	0.000	0.000	0.000	0.000	0.000	0.000
	.	.	.	.	.	.	.	.	.
hrrnum=2	0.059	-0.031	1.109*	-0.014	-0.361	0.677	-0.208	-0.224	0.219*
	0.2318	0.2302	0.4500	0.2906	0.5276	0.3760	0.3105	0.3025	0.1077
hrrnum=5	-0.424**	-0.409**	0.202	-0.371*	0.108	-0.145	-0.223	-0.250	0.127*
	0.1374	0.1371	0.2136	0.1793	0.2386	0.3306	0.1737	0.1882	0.0610
hrrnum=6	-0.136	-0.142	-0.056	-0.063	-0.070	-0.118	-0.008	-0.154	-0.112**
	0.0806	0.0814	0.1275	0.1133	0.1623	0.2042	0.1053	0.1161	0.0383
hrrnum=7	-0.237	-0.613	-0.464	-0.281	0.128	-0.124	0.083	0.409	-0.273
	0.4081	0.4027	0.4818	0.5086	0.8382	1.0795	0.5546	0.5589	0.2341
hrrnum=9	0.079	-0.193	0.000	0.598	0.707	0.000	-0.368	-0.138	0.445*
	0.4338	0.4366	.	0.7709	0.8053	.	0.6618	0.5235	0.2253
hrrnum=10	-0.790***	-0.756***	-0.037	-0.381*	-0.112	0.361	-0.254	-0.054	0.306***
	0.1578	0.1519	0.2348	0.1849	0.3133	0.3369	0.1843	0.2187	0.0653
hrrnum=11	-0.115	-0.177**	0.234*	0.102	-0.398**	-0.305	0.002	-0.154	-0.003
	0.0647	0.0653	0.1059	0.0945	0.1417	0.1648	0.0815	0.0922	0.0302
hrrnum=12	-0.246***	-0.204***	0.072	-0.147	-0.203	-0.168	-0.084	-0.085	0.066*
	0.0566	0.0569	0.0883	0.0790	0.1113	0.1304	0.0705	0.0756	0.0258
hrrnum=14	-0.278*	-0.222*	-0.110	-0.044	-0.541*	-0.577	-0.237	-0.275	0.014
	0.1082	0.1094	0.1641	0.1571	0.2659	0.3130	0.1218	0.1618	0.0492
hrrnum=15	0.121	0.149*	0.309**	-0.061	-0.431**	-0.200	0.050	-0.121	-0.097**
	0.0701	0.0711	0.1152	0.0984	0.1448	0.1646	0.0822	0.0929	0.0304
hrrnum=16	-0.100	0.113	-0.032	-0.553***	-0.220	0.138	-0.052	-0.028	-0.011
	0.0866	0.0875	0.1327	0.1086	0.1768	0.1868	0.1049	0.1127	0.0383
hrrnum=18	-0.070	0.118	-0.080	-0.146	0.074	0.102	-0.899***	0.457**	-0.229***
	0.1528	0.1520	0.2277	0.1967	0.2625	0.2771	0.1629	0.1645	0.0638
hrrnum=19	-0.111	0.032	0.029	-0.217*	-0.064	0.030	-0.414***	0.041	-0.088**
	0.0629	0.0631	0.0977	0.0861	0.1192	0.1357	0.0772	0.0805	0.0285
hrrnum=21	-0.053	0.058	0.187	-0.273**	-0.297	-0.219	-0.169	-0.268*	0.008
	0.0746	0.0753	0.1212	0.1011	0.1570	0.1773	0.0887	0.1058	0.0333
hrrnum=22	-0.540*	-0.445	-0.574*	-0.555*	0.072	-0.035	-0.770**	-0.151	0.328**
	0.2529	0.2339	0.2743	0.2610	0.3922	0.4491	0.2915	0.2751	0.1095
hrrnum=23	0.003	-0.034	0.313**	0.357***	-0.678***	-0.529**	-0.251**	-0.124	-0.504***
	0.0704	0.0707	0.1109	0.1057	0.1523	0.1777	0.0926	0.0950	0.0346

© 2022 Bakre S et al. *JAMA Health Forum.*

hrrnum=25	-0.305	0.035	-0.261	-0.595**	-0.228	-0.337	-0.467*	0.130	-0.066
	0.1823	0.1847	0.2522	0.2100	0.2784	0.3579	0.2062	0.1688	0.0771
hrrnum=31	-0.497***	-0.202	-0.280	-0.719***	-0.247	-0.988***	-0.058	0.088	-0.045
	0.1227	0.1199	0.1885	0.1558	0.2277	0.2974	0.1417	0.1292	0.0524
hrrnum=33	-0.407***	-0.303***	-0.244	-0.330**	-0.418**	-0.326	-0.150	-0.266*	-0.010
	0.0841	0.0839	0.1265	0.1169	0.1544	0.2086	0.0938	0.1063	0.0332
hrrnum=43	-0.421**	-0.083	-0.480**	-0.592***	0.100	0.116	-0.639***	0.363**	-0.049
	0.1511	0.1462	0.1785	0.1643	0.1919	0.2452	0.1825	0.1270	0.0615
hrrnum=56	-0.047	-0.029	0.223**	0.151*	-0.481***	-0.283*	-0.170*	-0.121	-0.436***
	0.0534	0.0538	0.0832	0.0751	0.1042	0.1221	0.0687	0.0712	0.0252
hrrnum=58	0.127	0.232	0.552	0.668	0.357	0.763*	-0.202	-0.149	0.433***
	0.2552	0.2597	0.4434	0.3770	0.3552	0.3324	0.2502	0.2745	0.0981
hrrnum=62	-0.080	0.057	0.322	-0.971***	-0.567	-0.726	-0.171	-0.330	0.071
	0.1488	0.1513	0.2657	0.1733	0.3420	0.5261	0.1661	0.2266	0.0615
hrrnum=65	-0.369**	-0.330*	-0.351	0.115	-0.892*	0.183	-0.144	0.361*	-0.030
	0.1323	0.1321	0.1870	0.1979	0.3717	0.3282	0.1593	0.1695	0.0610
hrrnum=69	0.065	-0.027	0.347	0.531*	-0.462	-0.621	0.351*	-0.342	-0.274***
	0.1341	0.1359	0.2339	0.2404	0.2638	0.3801	0.1781	0.1992	0.0548
hrrnum=73	-0.235	0.061	-0.239	-0.541*	-0.494	-0.283	-0.890***	-0.270	-0.234**
	0.1952	0.1922	0.2756	0.2367	0.3769	0.4051	0.1964	0.2451	0.0776
hrrnum=77	-0.554***	-0.363***	-0.338**	-0.588***	-0.443**	-0.319*	-0.013	-0.052	-0.094**
	0.0738	0.0725	0.1066	0.0943	0.1392	0.1596	0.0885	0.0909	0.0321
hrrnum=78	-0.489	-0.214	-0.574	-0.422	0.000	1.004	0.008	-0.179	0.233
	0.4402	0.4077	0.4626	0.4445	.	0.6812	0.4005	0.4772	0.1729
hrrnum=79	-0.223**	-0.239***	-0.042	-0.027	-0.333*	-0.424*	-0.206*	-0.161	-0.122***
	0.0708	0.0707	0.1086	0.0994	0.1452	0.1808	0.0908	0.0988	0.0343
hrrnum=80	-0.236**	-0.287***	0.146	-0.230*	-0.485**	-0.225	-0.458***	-0.038	-0.103**
	0.0827	0.0821	0.1234	0.1097	0.1599	0.1863	0.1015	0.1019	0.0359
hrrnum=81	-0.419***	-0.276***	-0.084	-0.448***	-0.192	-0.618**	-0.378***	-0.110	0.019
	0.0830	0.0828	0.1231	0.1106	0.1514	0.2350	0.0876	0.1049	0.0325
hrrnum=82	-0.238*	-0.313***	0.129	0.051	0.004	0.144	-0.384**	0.048	-0.107**
	0.0927	0.0921	0.1435	0.1336	0.1731	0.2193	0.1197	0.1155	0.0412
hrrnum=83	0.029	0.298	-0.163	0.458	-0.851	-0.310	-0.489	-0.388	-0.380*
	0.2777	0.2883	0.4039	0.4865	1.0328	1.0601	0.3650	0.6364	0.1516
hrrnum=85	-0.148	-0.305	-0.116	-0.261	-0.971	-0.312	-0.127	-0.216	-0.209*
	0.2379	0.2430	0.3819	0.3169	0.5286	0.6001	0.2360	0.3182	0.0917
hrrnum=86	0.593	0.690	-0.029	0.907	0.000	0.000	-1.175**	-0.028	0.307*
	0.4065	0.4545	0.5449	0.7172	.	.	0.3824	0.4550	0.1548
hrrnum=87	-0.410	-0.392	-0.309	-0.408	0.000	0.000	0.520	0.305	0.123
	0.6537	0.6001	0.8766	0.7751	.	.	0.8198	0.6954	0.2503
hrrnum=89	-0.392***	-0.335***	0.052	-0.266*	-0.722**	-0.326	-0.149	-0.270	0.063

© 2022 Bakre S et al. *JAMA Health Forum.*

	0.0966	0.0931	0.1383	0.1216	0.2232	0.2367	0.1018	0.1435	0.0400
hrrnum=91	-0.147	-0.132	0.364	-0.144	0.128	0.052	0.038	-0.046	-0.023
	0.1638	0.1633	0.2674	0.2162	0.3140	0.3785	0.2029	0.2291	0.0817
hrrnum=96	-0.238**	-0.197*	0.069	-0.297*	-0.417*	0.037	-0.251*	-0.044	-0.066
	0.0916	0.0910	0.1410	0.1213	0.1854	0.2074	0.1034	0.1176	0.0387
hrrnum=101	-0.160	0.062	0.526	-0.452*	0.331	0.099	-0.603***	0.348	-0.011
	0.1665	0.1684	0.2934	0.2112	0.3179	0.4026	0.1693	0.2220	0.0734
hrrnum=102	-0.182*	-0.064	0.092	-0.358**	-0.485*	-0.897***	-0.350***	-0.277*	0.120***
	0.0880	0.0890	0.1377	0.1145	0.1896	0.2608	0.0940	0.1249	0.0363
hrrnum=103	-0.324***	-0.258***	0.444***	-0.327***	-0.448***	-0.841***	-0.270***	-0.317***	-0.039
	0.0660	0.0665	0.1127	0.0911	0.1294	0.1679	0.0766	0.0891	0.0285
hrrnum=104	-0.518**	-0.316	0.153	-0.692**	-0.679	-0.443	-0.315	-0.090	-0.024
	0.1856	0.1850	0.3063	0.2261	0.3903	0.4397	0.1682	0.2195	0.0658
hrrnum=105	-0.733***	-0.231	-0.122	-1.133***	-0.586	-0.329	-0.201	0.142	0.184***
	0.1651	0.1537	0.2355	0.1768	0.3475	0.3568	0.1399	0.1783	0.0559
hrrnum=106	0.067	0.093	1.168*	-0.031	0.154	0.153	-0.428	-0.630	0.061
	0.2176	0.2171	0.5134	0.3069	0.4077	0.4700	0.2203	0.3624	0.0940
hrrnum=107	-0.486**	-0.274	-0.043	-0.584***	-0.643	0.120	0.127	-0.275	0.238***
	0.1494	0.1451	0.2159	0.1768	0.3789	0.3261	0.1751	0.2224	0.0678
hrrnum=109	0.003	-0.001	0.340**	-0.009	-0.164	0.204	0.590***	-0.037	0.003
	0.0752	0.0758	0.1238	0.1054	0.1397	0.1638	0.1067	0.0945	0.0326
hrrnum=110	0.306***	0.378***	0.310***	0.012	0.054	-0.012	0.243***	-0.078	-0.016
	0.0573	0.0584	0.0909	0.0806	0.1056	0.1259	0.0738	0.0742	0.0258
hrrnum=111	0.157**	0.163**	0.387***	0.141	-0.060	0.106	0.392***	-0.137	0.040
	0.0585	0.0592	0.0946	0.0836	0.1097	0.1291	0.0770	0.0768	0.0264
hrrnum=112	0.006	0.099	0.043	-0.148	0.076	0.164	0.137	-0.293**	-0.112***
	0.0661	0.0667	0.1042	0.0917	0.1341	0.1547	0.0853	0.0980	0.0317
hrrnum=113	-0.040	-0.037	0.393***	-0.022	-0.128	0.245*	-0.161*	-0.072	0.036
	0.0540	0.0543	0.0858	0.0760	0.1065	0.1230	0.0697	0.0912	0.0254
hrrnum=115	-0.060	-0.072	0.096	0.295*	-0.114	0.139	0.314**	-0.194	-0.158***
	0.0882	0.0890	0.1404	0.1395	0.1727	0.1876	0.1181	0.1152	0.0395
hrrnum=116	0.262***	0.139	0.655***	0.672***	-0.206	0.022	0.388***	-0.189*	-0.173***
	0.0740	0.0750	0.1306	0.1223	0.1307	0.1546	0.0947	0.0911	0.0315
hrrnum=118	0.212***	0.141*	0.442***	0.624***	-0.320**	0.094	0.224**	-0.073	-0.124***
	0.0556	0.0561	0.0889	0.0841	0.1063	0.1237	0.0728	0.0729	0.0255
hrrnum=119	0.280***	0.164**	0.599***	0.696***	-0.465***	-0.129	0.522***	-0.269***	-0.286***
	0.0603	0.0611	0.0996	0.0938	0.1198	0.1349	0.0792	0.0815	0.0275
hrrnum=120	-0.140	-0.104	0.062	-0.232*	0.302*	0.328*	-0.027	0.224*	0.018
	0.0785	0.0783	0.1233	0.1098	0.1430	0.1614	0.1013	0.0985	0.0375
hrrnum=122	0.301***	0.149*	0.668***	0.709***	-0.248	0.194	0.350***	-0.164	-0.264***
	0.0680	0.0687	0.1168	0.1109	0.1305	0.1410	0.0941	0.0897	0.0317

© 2022 Bakre S et al. *JAMA Health Forum.*

hrrnum=123	-0.104	-0.097	-0.073	-0.006	-0.065	0.207	-0.155*	-0.078	-0.083**
	0.0607	0.0610	0.0938	0.0864	0.1198	0.1333	0.0788	0.0830	0.0290
hrrnum=124	0.019	0.014	0.001	0.254	-0.119	0.250	0.218	-0.077	-0.044
	0.1550	0.1553	0.2309	0.2361	0.3267	0.3205	0.2119	0.2124	0.0795
hrrnum=127	0.156**	-0.048	0.769***	1.019***	-0.375***	0.106	-0.007	-0.002	-0.124***
	0.0594	0.0596	0.0962	0.0926	0.1135	0.1272	0.0797	0.0773	0.0284
hrrnum=129	0.387***	0.343***	0.368***	0.723***	-0.335**	-0.261	0.679***	-0.362***	-0.347***
	0.0594	0.0601	0.0944	0.0929	0.1183	0.1361	0.0815	0.0833	0.0283
hrrnum=130	0.053	-0.026	0.260**	0.446***	-0.210*	0.010	0.122	-0.144*	-0.248***
	0.0533	0.0536	0.0840	0.0775	0.1035	0.1195	0.0696	0.0717	0.0252
hrrnum=131	0.040	-0.035	0.630***	0.376*	-0.589*	0.329	0.075	-0.027	0.107*
	0.1036	0.1051	0.1863	0.1628	0.2614	0.2214	0.1353	0.1427	0.0485
hrrnum=133	-0.315	0.233	-0.969***	-0.638**	0.075	0.117	-0.223	-0.279	0.043
	0.1841	0.1799	0.2186	0.2227	0.3036	0.3184	0.1983	0.2382	0.0785
hrrnum=134	-0.058	0.009	-0.257**	-0.130	0.033	0.138	-0.253**	-0.072	0.116***
	0.0642	0.0650	0.0989	0.0905	0.1251	0.1433	0.0829	0.0883	0.0301
hrrnum=137	0.204*	0.205*	0.213	0.372**	-0.318*	-0.194	0.446***	-0.193	-0.201***
	0.0817	0.0838	0.1301	0.1308	0.1550	0.1796	0.1053	0.1079	0.0345
hrrnum=139	0.216*	0.190*	0.415**	0.481***	-0.342*	0.278	0.465***	0.183	-0.140***
	0.0869	0.0885	0.1416	0.1386	0.1643	0.1641	0.1136	0.0996	0.0372
hrrnum=140	0.009	0.015	0.314*	0.080	-0.033	-0.163	0.214*	-0.011	-0.172***
	0.0763	0.0771	0.1268	0.1109	0.1502	0.1786	0.1029	0.1046	0.0367
hrrnum=141	0.077	0.007	0.275*	0.296**	-0.155	-0.012	0.236**	-0.245**	-0.101**
	0.0654	0.0662	0.1071	0.0989	0.1283	0.1468	0.0860	0.0916	0.0309
hrrnum=142	0.238*	0.297**	0.698***	-0.090	-0.218	0.242	0.478***	-0.165	-0.198***
	0.0945	0.0957	0.1670	0.1320	0.1985	0.2017	0.1244	0.1373	0.0452
hrrnum=144	-0.177***	-0.225***	0.372***	-0.019	0.168	0.145	-0.055	-0.043	0.042
	0.0536	0.0539	0.0845	0.0753	0.1037	0.1212	0.0688	0.0726	0.0252
hrrnum=145	-0.034	0.109	0.535***	-0.399***	0.003	0.328*	0.400***	-0.010	-0.038
	0.0699	0.0709	0.1190	0.0942	0.1360	0.1505	0.0926	0.0929	0.0324
hrrnum=146	-0.267**	-0.109	0.002	-0.478***	-0.399*	-0.409	-0.004	-0.244	-0.250***
	0.0926	0.0914	0.1431	0.1224	0.1898	0.2341	0.1265	0.1390	0.0463
hrrnum=147	-0.206***	-0.105	0.153	-0.331***	-0.036	0.132	0.018	-0.054	0.048
	0.0599	0.0600	0.0946	0.0827	0.1163	0.1315	0.0784	0.0809	0.0285
hrrnum=148	-0.216*	-0.309**	0.148	0.100	-0.479	0.060	0.202	0.023	0.062
	0.1082	0.1090	0.1752	0.1573	0.2578	0.2292	0.1443	0.1467	0.0521
hrrnum=149	0.166*	0.153*	0.250*	0.219*	-0.329*	-0.045	0.181*	-0.191*	-0.124***
	0.0654	0.0662	0.1068	0.0988	0.1406	0.1514	0.0890	0.0954	0.0326
hrrnum=150	-0.270	-0.196	2.116	0.143	-0.811	1.028	-0.668	-0.851	0.222
	0.3268	0.3293	1.0828	0.5237	1.0005	0.5326	0.3707	0.5778	0.1332
hrrnum=151	-0.170**	-0.006	0.301**	-0.489***	-0.368**	-0.669***	-0.138	-0.350***	0.163***

© 2022 Bakre S et al. *JAMA Health Forum.*

	0.0639	0.0643	0.1006	0.0846	0.1250	0.1592	0.0779	0.0857	0.0280
hrrnum=152	-0.583	-0.338	-0.634	-1.375*	0.093	0.000	-0.503	0.331	0.386
	0.5743	0.5257	0.6205	0.5619	1.0135	.	0.6125	0.5612	0.2207
hrrnum=154	0.154	0.077	0.417**	0.340**	-0.115	0.388*	-0.223*	0.001	0.084*
	0.0790	0.0806	0.1396	0.1267	0.1606	0.1749	0.0947	0.1102	0.0361
hrrnum=155	-0.204***	-0.122*	0.118	-0.121	0.097	0.275*	-0.355***	0.131	-0.005
	0.0600	0.0604	0.0944	0.0846	0.1129	0.1325	0.0767	0.0784	0.0280
hrrnum=156	-0.061	-0.045	0.041	-0.051	0.167	0.375**	-0.034	0.142	-0.041
	0.0571	0.0575	0.0883	0.0799	0.1067	0.1254	0.0750	0.0742	0.0267
hrrnum=158	-0.195**	-0.194**	0.278**	-0.104	0.090	0.300*	-0.244**	0.002	0.019
	0.0614	0.0618	0.0992	0.0874	0.1166	0.1352	0.0774	0.0812	0.0282
hrrnum=161	0.064	0.125*	0.265**	-0.025	-0.276*	0.277*	-0.001	-0.058	-0.011
	0.0618	0.0629	0.0997	0.0884	0.1176	0.1350	0.0785	0.0795	0.0274
hrrnum=163	0.115	0.091	0.443***	0.109	-0.054	0.135	-0.078	0.037	-0.012
	0.0651	0.0664	0.1099	0.0954	0.1278	0.1533	0.0815	0.0866	0.0295
hrrnum=164	-0.168**	-0.144*	0.096	-0.242**	0.132	0.489***	-0.075	0.214**	0.116***
	0.0643	0.0647	0.1009	0.0888	0.1220	0.1377	0.0829	0.0826	0.0296
hrrnum=166	-0.094	-0.055	0.201*	-0.152	0.093	0.379**	-0.157*	-0.056	0.005
	0.0572	0.0578	0.0907	0.0801	0.1081	0.1258	0.0725	0.0756	0.0261
hrrnum=170	0.040	0.096	0.523***	-0.038	0.206	0.205	-0.355***	0.066	0.221***
	0.0745	0.0752	0.1278	0.1065	0.1318	0.1573	0.0909	0.0938	0.0331
hrrnum=171	-0.229**	-0.186*	0.347**	-0.221*	0.297*	0.321	-0.152	-0.017	0.090**
	0.0774	0.0774	0.1288	0.1038	0.1406	0.1640	0.0973	0.1021	0.0350
hrrnum=172	0.124	0.260***	0.273*	-0.158	0.018	0.206	-0.062	0.130	0.263***
	0.0687	0.0695	0.1063	0.0917	0.1258	0.1444	0.0869	0.0849	0.0303
hrrnum=173	-0.312***	-0.270***	0.532***	-0.209	0.331*	0.201	-0.251*	0.332**	0.162***
	0.0815	0.0811	0.1390	0.1108	0.1502	0.1776	0.0985	0.1023	0.0367
hrrnum=175	-0.085	-0.047	0.440*	-0.029	0.089	0.566**	0.177	-0.074	-0.079
	0.0992	0.1003	0.1752	0.1478	0.1752	0.1851	0.1267	0.1311	0.0431
hrrnum=179	0.144*	0.091	0.728***	0.378***	0.241*	0.339*	0.200*	0.117	0.112***
	0.0660	0.0668	0.1154	0.1006	0.1193	0.1382	0.0833	0.0826	0.0297
hrrnum=180	-0.162	-0.193*	0.363*	-0.162	0.191	0.428*	0.396**	0.151	0.195***
	0.0887	0.0889	0.1502	0.1231	0.1594	0.1733	0.1282	0.1136	0.0403
hrrnum=181	-0.213*	-0.160	0.135	-0.343**	0.072	0.061	0.070	0.263*	0.025
	0.0923	0.0914	0.1472	0.1199	0.1791	0.1979	0.1155	0.1192	0.0424
hrrnum=183	-0.335***	-0.257***	0.143	-0.377***	-0.020	0.109	-0.061	-0.178*	0.093***
	0.0534	0.0536	0.0836	0.0739	0.1038	0.1202	0.0685	0.0723	0.0249
hrrnum=184	-0.246***	-0.291***	0.393**	-0.055	0.025	0.038	0.977***	-0.126	0.074*
	0.0725	0.0727	0.1249	0.1033	0.1439	0.1737	0.1151	0.1014	0.0331
hrrnum=185	-0.401***	-0.248**	0.116	-0.785***	-0.114	-0.149	-0.055	-0.222	-0.178***
	0.0866	0.0864	0.1401	0.1090	0.1696	0.1972	0.1129	0.1221	0.0415

© 2022 Bakre S et al. *JAMA Health Forum.*

hrrnum=186	-0.056	-0.036	0.075	0.060	-0.268	0.151	-0.166	0.005	-0.040
	0.0878	0.0885	0.1402	0.1250	0.1822	0.1988	0.1138	0.1231	0.0423
hrrnum=187	-0.202***	-0.059	0.173	-0.398***	-0.053	-0.021	-0.273***	-0.241**	-0.104***
	0.0614	0.0617	0.0986	0.0835	0.1153	0.1361	0.0768	0.0831	0.0279
hrrnum=188	-0.296***	-0.268***	0.164	-0.263**	0.002	0.293	-0.393***	-0.210*	-0.060
	0.0731	0.0731	0.1147	0.0976	0.1366	0.1517	0.0911	0.1011	0.0339
hrrnum=190	0.343***	0.662***	0.510***	-0.369***	0.066	0.051	0.181*	0.011	0.187***
	0.0719	0.0765	0.1253	0.0978	0.1273	0.1536	0.0870	0.0899	0.0307
hrrnum=191	0.167**	0.226***	0.857***	-0.077	-0.021	-0.143	0.161*	0.012	0.041
	0.0626	0.0634	0.1114	0.0886	0.1174	0.1393	0.0790	0.0808	0.0281
hrrnum=192	0.193***	0.353***	0.464***	-0.269***	-0.185	-0.038	-0.153*	-0.035	-0.035
	0.0583	0.0595	0.0956	0.0810	0.1105	0.1291	0.0727	0.0761	0.0264
hrrnum=193	0.063	0.080	0.298	-0.001	0.549*	-0.340	0.536**	-0.012	0.344***
	0.1358	0.1386	0.2359	0.1953	0.2304	0.3980	0.1839	0.1811	0.0563
hrrnum=194	-0.281***	0.471***	0.682***	-1.247***	0.016	-0.414*	-0.061	0.036	0.210***
	0.0814	0.0839	0.1444	0.0971	0.1447	0.1948	0.0979	0.1012	0.0344
hrrnum=195	0.031	0.435***	0.293*	-0.844***	-0.234	-0.027	0.130	0.128	0.158***
	0.0769	0.0797	0.1269	0.0968	0.1409	0.1718	0.0992	0.0936	0.0325
hrrnum=196	0.324*	0.398**	0.713**	0.084	0.095	-0.212	-0.133	0.378**	0.089
	0.1284	0.1311	0.2406	0.1810	0.1991	0.2738	0.1402	0.1286	0.0503
hrrnum=197	0.217*	0.523***	0.225	-0.331**	0.084	-0.476*	0.517***	0.083	0.234***
	0.0871	0.0913	0.1358	0.1153	0.1431	0.2198	0.1133	0.1046	0.0364
hrrnum=200	0.119	0.316***	0.255*	-0.396***	0.127	-0.033	0.124	-0.025	0.078*
	0.0707	0.0726	0.1167	0.0941	0.1319	0.1611	0.0900	0.0913	0.0313
hrrnum=201	-0.028	0.370***	0.028	-0.833***	-0.355**	-0.696***	-0.255**	-0.174	-0.075*
	0.0656	0.0674	0.1042	0.0860	0.1310	0.1704	0.0804	0.0896	0.0294
hrrnum=203	-0.889**	-0.580	0.267	-0.676*	0.268	1.030**	0.907*	0.055	0.180
	0.3042	0.2969	0.4591	0.3387	0.4212	0.3653	0.4046	0.3002	0.1108
hrrnum=204	-0.259***	-0.295***	0.356***	0.024	0.020	0.086	-0.232**	-0.039	0.082**
	0.0642	0.0641	0.1037	0.0899	0.1219	0.1353	0.0822	0.0853	0.0300
hrrnum=205	-0.104	-0.110	0.214*	0.027	-0.007	0.026	-0.183*	-0.121	-0.002
	0.0577	0.0580	0.0916	0.0819	0.1091	0.1257	0.0736	0.0765	0.0267
hrrnum=207	0.184*	0.202*	0.445**	0.192	0.097	-0.073	0.466***	-0.233*	0.034
	0.0849	0.0857	0.1406	0.1256	0.1539	0.1855	0.1163	0.1147	0.0387
hrrnum=208	0.010	0.015	0.460***	-0.027	0.026	0.215	0.767***	-0.138	-0.001
	0.0751	0.0756	0.1276	0.1066	0.1399	0.1511	0.1130	0.0969	0.0341
hrrnum=209	-0.236*	-0.118	-0.080	0.008	0.267	0.385	-0.402**	0.103	0.128**
	0.1053	0.1047	0.1534	0.1451	0.1908	0.2067	0.1406	0.1326	0.0495
hrrnum=210	0.423***	0.553***	0.491***	0.064	-0.201	-0.032	0.939***	-0.310**	-0.060
	0.0696	0.0718	0.1134	0.0970	0.1447	0.1689	0.0987	0.1032	0.0332
hrrnum=212	0.285	0.148	1.199***	0.856***	-0.149	0.173	0.420*	0.088	0.251***

© 2022 Bakre S et al. *JAMA Health Forum.*

	0.1483	0.1482	0.2976	0.2520	0.2791	0.2938	0.1965	0.1788	0.0641
hrrnum=213	0.383***	0.417***	0.499*	-0.030	-0.704*	0.133	0.684***	-0.162	0.002
	0.1153	0.1180	0.2027	0.1566	0.2955	0.2391	0.1657	0.1606	0.0531
hrrnum=214	-0.241	0.045	-0.024	-0.352	0.029	-0.583	-0.408*	0.117	-0.134
	0.1495	0.1491	0.2266	0.1903	0.2945	0.4118	0.1736	0.2055	0.0734
hrrnum=216	0.288***	0.373***	0.395**	0.001	0.164	0.265	0.387***	-0.177	0.262***
	0.0831	0.0863	0.1311	0.1137	0.1556	0.1934	0.1064	0.1130	0.0377
hrrnum=217	-0.272	0.044	-0.208	-0.977	0.000	0.736	0.987	1.113	-0.434
	0.7596	0.6764	0.8347	0.8584	.	1.1412	0.9841	0.8895	0.3179
hrrnum=218	0.039	0.224**	0.124	-0.179	-0.052	-0.165	0.317***	-0.125	0.199***
	0.0719	0.0730	0.1079	0.0943	0.1445	0.1922	0.0928	0.1026	0.0347
hrrnum=219	0.044	-0.101	0.072	0.398*	0.138	-0.096	-0.048	0.121	0.076
	0.1316	0.1327	0.1952	0.1988	0.2473	0.2874	0.1793	0.1812	0.0671
hrrnum=220	0.337**	0.320**	0.717***	0.189	-0.160	-0.128	0.444**	0.116	0.229***
	0.1054	0.1076	0.1860	0.1512	0.2372	0.2712	0.1360	0.1495	0.0506
hrrnum=221	0.504***	0.625***	0.758***	0.103	-0.006	-0.089	0.658***	0.109	0.479***
	0.0864	0.0895	0.1510	0.1175	0.1560	0.1790	0.1150	0.1039	0.0369
hrrnum=222	0.155**	0.389***	0.212*	-0.411***	-0.065	-0.170	0.385***	-0.207**	0.170***
	0.0582	0.0593	0.0921	0.0797	0.1077	0.1279	0.0741	0.0765	0.0260
hrrnum=223	-0.001	0.090	0.279**	-0.211**	0.021	0.297*	0.080	0.125	-0.005
	0.0550	0.0554	0.0869	0.0764	0.1067	0.1244	0.0711	0.1523	0.0258
hrrnum=225	0.228***	0.279***	0.343***	0.099	-0.123	-0.187	0.262***	-0.230*	0.002
	0.0589	0.0597	0.0945	0.0844	0.1169	0.1384	0.0770	0.0979	0.0276
hrrnum=226	-0.037	-0.117	0.459***	0.216*	-0.063	0.171	-0.189*	-0.021	-0.039
	0.0618	0.0623	0.1024	0.0908	0.1269	0.1530	0.0821	0.1700	0.0298
hrrnum=227	0.371***	0.399***	0.504***	0.145	0.062	0.280*	0.474***	-0.048	0.091***
	0.0542	0.0550	0.0867	0.0767	0.1024	0.1191	0.0699	0.0711	0.0248
hrrnum=230	0.420***	0.485***	0.374***	0.141	0.101	0.125	0.584***	-0.195*	-0.092**
	0.0641	0.0660	0.1040	0.0920	0.1144	0.1350	0.0815	0.0838	0.0283
hrrnum=231	0.262***	0.353***	0.264*	-0.094	0.036	0.306*	0.439***	0.022	-0.044
	0.0662	0.0679	0.1078	0.0911	0.1225	0.1410	0.0872	0.0865	0.0299
hrrnum=232	-0.115*	0.019	0.257**	-0.331***	-0.016	0.078	-0.030	0.049	0.117***
	0.0569	0.0575	0.0905	0.0787	0.1089	0.1274	0.0732	0.0751	0.0263
hrrnum=233	-0.319***	-0.255***	0.041	-0.155	-0.090	0.152	-0.246**	0.107	-0.080**
	0.0630	0.0632	0.0979	0.0894	0.1197	0.1346	0.0855	0.0815	0.0305
hrrnum=234	-0.213***	-0.181**	0.173	-0.038	-0.249*	0.086	0.007	-0.022	-0.023
	0.0601	0.0603	0.0937	0.0842	0.1185	0.1326	0.0807	0.0803	0.0288
hrrnum=235	-0.146*	-0.035	0.082	-0.141	-0.237	-0.049	0.187*	-0.146	-0.183***
	0.0653	0.0654	0.1016	0.0900	0.1313	0.1472	0.0871	0.0903	0.0317
hrrnum=236	-0.019	-0.012	0.369**	-0.059	-0.160	-0.306	0.482***	-0.355***	0.160***
	0.0700	0.0707	0.1128	0.0984	0.1282	0.1591	0.0940	0.0933	0.0307

© 2022 Bakre S et al. *JAMA Health Forum.*

hrrnum=238	-0.269*	-0.180	0.228	-0.127	-0.086	0.271	-0.079	-0.123	0.211***
	0.1106	0.1079	0.1669	0.1410	0.1832	0.1912	0.1286	0.1288	0.0459
hrrnum=239	0.058	0.147	0.355	-0.107	-0.341	-0.297	0.438*	0.155	0.214***
	0.1471	0.1496	0.2315	0.1882	0.2825	0.3414	0.2056	0.1732	0.0643
hrrnum=240	-0.223	-0.120	-0.243	0.069	-0.400	0.257	-0.042	0.164	0.181*
	0.1892	0.1887	0.2404	0.2509	0.4464	0.3718	0.2431	0.2495	0.0840
hrrnum=242	0.155	0.159*	0.457***	0.024	-0.481**	-0.881***	0.312**	-0.483***	0.206***
	0.0791	0.0809	0.1324	0.1132	0.1641	0.2287	0.1060	0.1205	0.0359
hrrnum=243	-0.063	0.124	0.363	-0.306	0.476*	0.278	0.557**	-0.152	0.078
	0.1350	0.1371	0.2278	0.1766	0.1897	0.2658	0.1769	0.1600	0.0516
hrrnum=244	-0.324***	-0.166*	0.198	-0.551***	-0.045	0.358*	-0.106	-0.012	-0.060
	0.0683	0.0684	0.1094	0.0910	0.1341	0.1470	0.0883	0.0928	0.0322
hrrnum=245	-0.124	-0.126	0.183	0.103	-0.166	0.207	0.075	0.039	-0.098**
	0.0640	0.0644	0.1005	0.0922	0.1249	0.1435	0.0848	0.0850	0.0300
hrrnum=246	-0.044	0.027	0.492***	-0.223*	-0.077	0.105	0.381***	-0.002	0.197***
	0.0843	0.0840	0.1402	0.1105	0.1557	0.1708	0.1140	0.1045	0.0383
hrrnum=248	-0.221	-0.063	0.481	-0.144	0.055	0.204	-0.111	0.028	0.280*
	0.2706	0.2692	0.4852	0.3732	0.5451	0.5955	0.2817	0.3206	0.1165
hrrnum=249	0.185*	0.262***	0.557***	-0.034	-0.352*	0.035	0.377***	-0.176	0.128***
	0.0737	0.0751	0.1280	0.1033	0.1456	0.1590	0.0985	0.0990	0.0323
hrrnum=250	-0.171*	-0.085	0.766***	-0.357***	-0.208	-0.296	0.180	0.076	0.203***
	0.0800	0.0802	0.1357	0.1051	0.1419	0.1838	0.0972	0.0953	0.0331
hrrnum=251	-0.040	0.042	0.604***	-0.395***	0.052	0.094	0.165	0.081	0.091**
	0.0704	0.0712	0.1229	0.0944	0.1229	0.1503	0.0887	0.0862	0.0301
hrrnum=253	0.034	0.283	0.419	-0.524**	0.059	0.310	0.537**	0.185	0.411***
	0.1573	0.1596	0.2400	0.1850	0.2517	0.3111	0.2037	0.1850	0.0599
hrrnum=254	0.063	0.131	0.552*	-0.200	0.329	-0.316	0.288	0.192	-0.000
	0.1446	0.1491	0.2806	0.1935	0.2032	0.3444	0.1838	0.1523	0.0544
hrrnum=256	-0.125	-0.098	0.510**	-0.194	0.123	0.022	-0.298**	-0.136	-0.060
	0.0903	0.0913	0.1551	0.1242	0.1430	0.1930	0.1095	0.1036	0.0355
hrrnum=257	-0.500***	-0.252*	-0.478**	-0.547***	0.483*	0.248	-0.030	0.158	0.087
	0.1301	0.1255	0.1637	0.1595	0.1938	0.2222	0.1650	0.1522	0.0583
hrrnum=258	0.382***	0.324***	0.104	0.338*	0.116	0.122	0.245	-0.120	-0.022
	0.0964	0.0983	0.1533	0.1510	0.1975	0.2062	0.1271	0.1379	0.0465
hrrnum=259	-0.232**	-0.030	0.210	-0.564***	0.065	0.424**	-0.016	0.155	0.180***
	0.0753	0.0743	0.1156	0.0950	0.1385	0.1558	0.0914	0.0938	0.0337
hrrnum=260	-0.003	0.297*	-0.123	-0.534***	0.437*	0.154	-0.101	0.207	0.231***
	0.1177	0.1178	0.1608	0.1397	0.2108	0.2594	0.1495	0.1479	0.0555
hrrnum=261	-0.152	-0.076	-0.157	-0.245	-0.125	-0.012	-0.557**	0.044	-0.008
	0.1528	0.1526	0.2406	0.2076	0.3045	0.3613	0.1913	0.2036	0.0705
hrrnum=262	0.208*	0.186	0.680***	-0.032	0.011	0.330	0.113	0.298*	0.105*

© 2022 Bakre S et al. *JAMA Health Forum.*

	0.0944	0.0954	0.1750	0.1307	0.1987	0.1880	0.1224	0.1190	0.0444
hrrnum=263	-0.163	-0.056	-0.217	0.110	-0.260	-0.452	-0.085	-0.032	0.245**
	0.2207	0.2125	0.2763	0.2795	0.3989	0.4369	0.2689	0.2473	0.0903
hrrnum=264	0.079	0.347***	0.303*	-0.303*	-0.219	-0.176	0.542***	0.124	0.071
	0.0903	0.0936	0.1444	0.1201	0.1796	0.2162	0.1181	0.1128	0.0389
hrrnum=267	-0.293*	-0.026	-0.101	-0.341*	-0.047	-0.380	0.118	0.110	0.129*
	0.1231	0.1235	0.1719	0.1496	0.2146	0.2534	0.1558	0.1423	0.0530
hrrnum=268	0.090	0.079	0.610***	0.065	0.009	-0.114	0.080	-0.064	0.043
	0.0600	0.0606	0.1008	0.0871	0.1161	0.1378	0.0760	0.0797	0.0274
hrrnum=270	0.342***	0.384***	0.421***	0.067	0.055	-0.128	0.499***	-0.142	0.063*
	0.0628	0.0640	0.1002	0.0877	0.1161	0.1376	0.0809	0.0821	0.0280
hrrnum=273	0.075	0.083	0.462***	0.036	0.037	-0.088	0.545***	0.004	0.188***
	0.0545	0.0550	0.0867	0.0766	0.1043	0.1237	0.0714	0.0723	0.0252
hrrnum=274	-0.171*	0.013	0.213	-0.457***	-0.330*	-0.118	0.123	-0.181	0.098**
	0.0724	0.0728	0.1152	0.0945	0.1417	0.1620	0.0875	0.0926	0.0311
hrrnum=275	-1.159*	-1.181*	-1.502**	-1.222*	0.000	0.161	-0.119	0.179	-0.057
	0.5313	0.5103	0.5578	0.4826	.	1.0740	0.5677	0.5726	0.2120
hrrnum=276	-0.363***	-0.050	-0.008	-0.904***	0.213	0.003	0.173	0.110	0.345***
	0.1082	0.1068	0.1610	0.1225	0.1800	0.2300	0.1276	0.1217	0.0405
hrrnum=277	-0.223**	0.072	0.165	-0.667***	-0.158	-0.253	0.075	0.108	-0.254***
	0.0821	0.0827	0.1347	0.1060	0.1451	0.1789	0.0966	0.0943	0.0350
hrrnum=278	-0.316***	0.228***	-0.887***	-0.383***	0.042	0.285*	-0.125	0.003	-0.040
	0.0608	0.0615	0.0879	0.0826	0.1153	0.1322	0.0754	0.0792	0.0276
hrrnum=279	-0.227***	-0.219***	0.017	-0.100	-0.182	-0.291*	-0.199**	-0.127	-0.131***
	0.0587	0.0587	0.0908	0.0820	0.1204	0.1408	0.0748	0.0834	0.0281
hrrnum=280	-0.050	-0.029	0.356**	-0.127	-0.211	-0.646**	1.026***	-0.097	0.273***
	0.0747	0.0750	0.1224	0.1028	0.1577	0.1974	0.1052	0.0994	0.0325
hrrnum=281	0.020	0.266***	0.791***	-0.423***	0.198	-0.029	0.109	0.170	0.455***
	0.0704	0.0712	0.1171	0.0908	0.1276	0.1546	0.0859	0.0892	0.0304
hrrnum=282	0.302***	0.391***	0.523***	-0.062	0.207	0.145	0.155	0.039	0.225***
	0.0645	0.0663	0.1105	0.0909	0.1171	0.1413	0.0812	0.0827	0.0283
hrrnum=283	-0.045	-0.105*	0.149	0.366***	-0.138	0.246*	-0.157*	-0.092	-0.034
	0.0521	0.0525	0.0815	0.0742	0.1013	0.1173	0.0677	0.0700	0.0246
hrrnum=284	0.114	0.080	0.151	0.389***	-0.329**	0.257	-0.191*	-0.004	-0.148***
	0.0585	0.0591	0.0917	0.0860	0.1180	0.1344	0.0770	0.0794	0.0278
hrrnum=285	-0.111	-0.167**	0.186*	0.169*	-0.011	0.048	-0.436***	-0.003	-0.073**
	0.0567	0.0570	0.0899	0.0821	0.1094	0.1318	0.0722	0.0764	0.0264
hrrnum=288	-0.113	-0.179**	0.085	0.222*	0.005	0.133	-0.449***	0.027	-0.236***
	0.0594	0.0598	0.0937	0.0866	0.1152	0.1405	0.0780	0.0829	0.0287
hrrnum=289	0.063	-0.046	0.311***	0.361***	-0.094	0.325*	-0.342***	-0.030	-0.150***
	0.0591	0.0594	0.0936	0.0863	0.1147	0.1343	0.0773	0.0811	0.0284

© 2022 Bakre S et al. *JAMA Health Forum.*

hrrnum=291	-0.059	-0.106	0.205	0.030	0.028	0.581***	-0.393***	0.033	0.056
	0.0760	0.0771	0.1248	0.1103	0.1458	0.1620	0.0975	0.1031	0.0357
hrrnum=292	0.209**	0.162*	0.490***	0.232*	-0.303*	0.087	-0.170	-0.033	-0.070*
	0.0715	0.0731	0.1189	0.1064	0.1455	0.1684	0.0912	0.0978	0.0324
hrrnum=293	-0.523***	-0.337***	-0.161	-0.774***	-0.012	-0.518	-0.544***	-0.134	0.195***
	0.1051	0.1014	0.1456	0.1213	0.2070	0.2991	0.1124	0.1433	0.0446
hrrnum=295	0.113	0.159**	0.460***	-0.057	0.107	0.214	0.262***	0.001	0.061*
	0.0604	0.0610	0.0987	0.0840	0.1120	0.1303	0.0783	0.0782	0.0272
hrrnum=296	-0.040	0.223	0.194	-0.539***	0.260	0.545*	0.373*	0.281	0.314***
	0.1239	0.1270	0.1948	0.1463	0.2188	0.2474	0.1675	0.1476	0.0541
hrrnum=297	-0.114	0.034	-0.070	-0.537***	-0.163	0.625***	-0.060	0.009	0.045
	0.0753	0.0753	0.1102	0.0952	0.1561	0.1640	0.1031	0.1023	0.0375
hrrnum=299	0.148*	0.139*	0.180	0.035	-0.046	0.081	0.372***	-0.140	-0.056
	0.0651	0.0658	0.1011	0.0925	0.1211	0.1413	0.0876	0.0873	0.0295
hrrnum=300	0.112	0.373	0.892*	-0.480*	-0.483	0.617*	0.264	0.376*	0.134
	0.1952	0.2006	0.3643	0.2248	0.3780	0.2866	0.2306	0.1909	0.0764
hrrnum=301	0.258***	0.151**	0.654***	0.588***	-0.367***	-0.056	-0.284***	-0.069	-0.358***
	0.0527	0.0531	0.0840	0.0765	0.1044	0.1224	0.0686	0.0714	0.0251
hrrnum=303	0.101	0.079	0.263**	0.073	-0.578***	-0.128	-0.332***	-0.149*	-0.432***
	0.0543	0.0547	0.0850	0.0762	0.1098	0.1275	0.0707	0.0744	0.0264
hrrnum=304	-0.017	-0.083	0.294*	0.181	0.231	0.159	0.199	0.076	0.005
	0.0847	0.0851	0.1381	0.1237	0.1479	0.1904	0.1116	0.1112	0.0388
hrrnum=307	0.284***	0.322***	0.610***	0.161	0.007	0.037	0.784***	-0.068	-0.150***
	0.0657	0.0670	0.1173	0.0975	0.1230	0.1431	0.0913	0.0872	0.0294
hrrnum=308	0.326***	0.256***	0.578***	0.466***	-0.018	0.238	0.179*	-0.090	-0.021
	0.0640	0.0650	0.1068	0.0982	0.1188	0.1402	0.0803	0.0831	0.0284
hrrnum=309	0.008	0.130	0.299**	-0.184*	-0.467***	-0.546**	0.000	-0.306**	-0.168***
	0.0683	0.0691	0.1128	0.0928	0.1385	0.1668	0.0830	0.0944	0.0305
hrrnum=311	-0.101	-0.109	0.273*	0.040	0.063	0.013	-0.038	-0.088	0.083*
	0.0713	0.0714	0.1166	0.1020	0.1405	0.1623	0.0914	0.0996	0.0340
hrrnum=312	0.098	0.148*	0.606***	0.007	0.007	0.094	0.580***	-0.163*	-0.056*
	0.0593	0.0600	0.0979	0.0829	0.1137	0.1324	0.0798	0.0808	0.0275
hrrnum=313	0.062	0.121	0.479***	-0.237*	0.107	-0.403*	0.041	-0.310***	-0.101***
	0.0665	0.0674	0.1110	0.0923	0.1206	0.1570	0.0823	0.0903	0.0300
hrrnum=314	0.177**	0.282***	0.655***	-0.193*	-0.127	-0.284	1.175***	-0.261**	-0.015
	0.0622	0.0629	0.1052	0.0855	0.1231	0.1487	0.0913	0.0879	0.0293
hrrnum=315	0.175*	0.153	0.766***	0.080	-0.617**	-0.043	0.191	-0.418**	-0.173***
	0.0835	0.0838	0.1566	0.1258	0.2051	0.1924	0.1070	0.1360	0.0393
hrrnum=318	0.106	0.147*	0.627***	0.067	0.080	0.101	0.366***	0.029	0.036
	0.0601	0.0606	0.0994	0.0850	0.1178	0.1388	0.0782	0.0825	0.0281
hrrnum=319	0.342***	0.289***	0.829***	0.435***	-0.111	-0.418*	0.575***	-0.162	-0.169***

© 2022 Bakre S et al. *JAMA Health Forum.*

	0.0682	0.0695	0.1214	0.1021	0.1402	0.1795	0.0890	0.0975	0.0319
hrrnum=320	-0.053	0.005	0.398***	-0.248**	0.050	-0.168	0.492***	-0.121	-0.005
	0.0680	0.0683	0.1128	0.0938	0.1268	0.1526	0.0916	0.0896	0.0309
hrrnum=321	-0.059	0.186	0.097	-0.273	0.301	-0.057	0.155	0.113	0.202***
	0.1225	0.1267	0.1883	0.1552	0.2066	0.2843	0.1397	0.1425	0.0494
hrrnum=322	0.527***	0.619***	0.966***	0.073	-0.054	0.361*	0.631***	0.167	0.188***
	0.0947	0.0995	0.1788	0.1359	0.1549	0.1802	0.1274	0.1026	0.0367
hrrnum=323	0.144	0.239	-0.011	0.036	0.000	-0.852	0.048	0.466	-0.027
	0.3606	0.3737	0.4043	0.4853	.	0.9932	0.4884	0.4044	0.1487
hrrnum=324	0.104	0.251	0.057	-0.445	-0.412	-0.302	0.102	0.272	0.142
	0.2023	0.2098	0.3145	0.2382	0.4861	0.5206	0.2353	0.2593	0.0859
hrrnum=325	-0.209**	-0.110	0.127	-0.407***	-0.006	-0.031	-0.111	0.021	0.203***
	0.0665	0.0668	0.1029	0.0894	0.1221	0.1468	0.0818	0.0847	0.0297
hrrnum=326	-0.043	0.009	0.294*	-0.126	0.113	0.150	-0.267**	-0.018	0.272***
	0.0716	0.0718	0.1171	0.1001	0.1300	0.1532	0.0880	0.0935	0.0319
hrrnum=327	-0.083	-0.156*	0.753***	0.002	0.134	-0.115	2.143***	-0.130	0.124***
	0.0640	0.0647	0.1136	0.0919	0.1173	0.1460	0.1251	0.0850	0.0289
hrrnum=328	-0.191***	-0.067	0.123	-0.418***	0.183	0.380**	0.003	0.105	0.189***
	0.0547	0.0550	0.0852	0.0753	0.1030	0.1202	0.0699	0.0715	0.0252
hrrnum=329	-0.198**	-0.167**	0.170	-0.277**	-0.036	0.073	-0.167*	0.074	0.108***
	0.0625	0.0625	0.0967	0.0844	0.1180	0.1347	0.0792	0.0806	0.0287
hrrnum=330	-0.452***	-0.384***	0.213	-0.339**	0.135	0.083	1.176***	-0.089	0.145***
	0.0809	0.0801	0.1266	0.1065	0.1337	0.1610	0.1427	0.0977	0.0352
hrrnum=331	-0.137	-0.133	0.318**	-0.059	-0.088	0.440**	0.640***	0.167	0.106**
	0.0744	0.0750	0.1233	0.1054	0.1423	0.1470	0.1075	0.0953	0.0339
hrrnum=332	-0.281	-0.423*	0.309	0.318	0.044	-0.683	0.603*	-0.366	0.243**
	0.1795	0.1749	0.3058	0.2934	0.3133	0.5050	0.2739	0.2498	0.0743
hrrnum=334	-0.254***	-0.130*	0.138	-0.440***	0.051	0.286*	0.195*	-0.178*	0.149***
	0.0583	0.0586	0.0911	0.0794	0.1112	0.1264	0.0768	0.0786	0.0272
hrrnum=335	-0.374**	-0.398***	-0.011	-0.359*	0.136	0.051	0.514**	0.107	0.158**
	0.1174	0.1151	0.1706	0.1510	0.2025	0.2409	0.1565	0.1403	0.0531
hrrnum=336	-0.386	-0.400	-0.472	-0.829	-0.819	0.136	-0.447	-0.655	-0.117
	0.5939	0.5666	0.7287	0.5895	1.0675	1.0574	0.5253	0.7911	0.2232
hrrnum=339	-0.111	0.005	0.115	-0.350***	-0.090	-0.152	0.016	0.008	0.174***
	0.0677	0.0682	0.1064	0.0902	0.1305	0.1541	0.0855	0.0890	0.0303
hrrnum=340	-0.427***	-0.199*	-0.207	-0.735***	0.222	0.105	-0.585***	0.203	0.078*
	0.0851	0.0838	0.1221	0.1039	0.1490	0.1886	0.0973	0.1079	0.0372
hrrnum=341	-0.184	-0.355	0.477	-0.586*	-0.517	-0.035	-0.336	-0.330	0.259**
	0.2286	0.2271	0.3408	0.2612	0.5176	0.4677	0.2302	0.2817	0.0818
hrrnum=342	-0.266**	-0.145	0.082	-0.472***	-0.424*	-0.110	-0.212	-0.141	0.171***
	0.1015	0.0994	0.1597	0.1330	0.2076	0.2165	0.1168	0.1325	0.0430

© 2022 Bakre S et al. *JAMA Health Forum.*

hrrnum=343	0.288	0.228	0.604	-0.089	-0.516	-0.167	-0.467*	-0.187	0.057
	0.1932	0.1993	0.3273	0.2633	0.4008	0.4198	0.1937	0.2725	0.0790
hrrnum=344	-0.152	0.167	0.057	-0.519**	-0.010	0.051	0.032	-0.079	0.269***
	0.1664	0.1637	0.2370	0.1918	0.3067	0.3751	0.1929	0.2145	0.0666
hrrnum=345	-0.338	-0.680	-0.808	-1.030	0.216	1.192	0.000	-0.783	0.378
	1.2339	1.1803	1.4801	1.3157	1.0409	1.1404	.	1.0160	0.2969
hrrnum=346	0.029	0.028	0.118	-0.060	0.102	0.166	-0.068	-0.034	-0.079**
	0.0670	0.0676	0.1076	0.0935	0.1230	0.1534	0.0846	0.0870	0.0303
hrrnum=347	-0.080	-0.085	-0.020	-0.206	0.190	0.136	0.085	-0.086	0.028
	0.1930	0.1950	0.3109	0.2608	0.3013	0.3678	0.2182	0.2380	0.0847
hrrnum=350	0.203**	0.331***	0.248*	0.033	-0.353*	-0.140	0.111	-0.119	0.016
	0.0728	0.0750	0.1222	0.1039	0.1411	0.1636	0.0924	0.0951	0.0320
hrrnum=351	0.211**	0.391***	0.056	-0.310**	-0.127	0.007	0.579***	0.008	0.140***
	0.0795	0.0820	0.1235	0.1070	0.1478	0.1776	0.1121	0.0992	0.0349
hrrnum=352	0.186**	0.177*	0.372**	0.047	0.029	0.004	0.040	-0.080	-0.149***
	0.0696	0.0709	0.1185	0.0990	0.1294	0.1623	0.0898	0.0939	0.0316
hrrnum=354	-0.004	-0.117	0.093	-0.173	-0.357	-0.586	0.541	0.603	0.095
	0.3615	0.3572	0.5189	0.4378	0.7728	1.0623	0.4092	0.4197	0.1534
hrrnum=355	0.160*	0.222**	0.587***	-0.001	-0.319*	-0.220	0.039	-0.172	-0.323***
	0.0702	0.0722	0.1282	0.1005	0.1277	0.1631	0.0869	0.0902	0.0301
hrrnum=356	0.037	0.013	0.041	0.162*	0.007	0.298*	-0.106	-0.013	0.007
	0.0547	0.0551	0.0855	0.0775	0.1046	0.1222	0.0704	0.0723	0.0255
hrrnum=357	0.124	0.205**	0.153	-0.315***	0.344**	0.256	-0.025	0.163	0.179***
	0.0681	0.0690	0.1070	0.0917	0.1215	0.1497	0.0833	0.0848	0.0304
hrrnum=358	-0.072	-0.094	0.156	0.126	-0.057	0.019	0.321**	0.032	-0.089*
	0.0828	0.0837	0.1368	0.1185	0.1494	0.1851	0.1065	0.1037	0.0366
hrrnum=359	-0.435*	-0.109	-0.320	-0.403	-0.242	-0.207	0.545*	-0.089	0.157
	0.1949	0.1899	0.2509	0.2284	0.3583	0.4398	0.2583	0.2378	0.0811
hrrnum=360	-0.044	-0.033	0.359*	-0.152	0.187	0.125	0.301*	-0.180	0.132**
	0.0953	0.0963	0.1579	0.1268	0.1726	0.2029	0.1222	0.1301	0.0426
hrrnum=362	-0.054	0.090	-0.193	-0.154	-0.453	-0.060	-0.419*	-0.046	-0.090
	0.1425	0.1477	0.2198	0.1949	0.3073	0.3144	0.1740	0.1800	0.0669
hrrnum=363	0.216**	0.253**	0.648***	0.089	0.247	-0.468*	0.347***	-0.076	-0.290***
	0.0790	0.0808	0.1434	0.1123	0.1393	0.2086	0.1021	0.1054	0.0356
hrrnum=364	0.429***	0.557***	0.192	0.026	-0.059	0.145	0.480***	-0.094	-0.079**
	0.0661	0.0683	0.1041	0.0930	0.1258	0.1432	0.0852	0.0879	0.0296
hrrnum=365	-0.090	-0.110	0.290*	0.115	-0.167	-0.246	0.182*	-0.125	-0.076*
	0.0681	0.0685	0.1128	0.1031	0.1448	0.1697	0.0877	0.0987	0.0331
hrrnum=366	-0.076	0.089	0.045	-0.278**	-0.316*	-0.154	-0.015	-0.145	-0.073*
	0.0680	0.0688	0.1067	0.0921	0.1497	0.1735	0.0878	0.0983	0.0328
hrrnum=367	-0.187	-0.216	0.281	0.067	0.117	-0.491	0.147	0.221	0.086

© 2022 Bakre S et al. *JAMA Health Forum.*

	0.1869	0.1870	0.3123	0.2894	0.3753	0.5275	0.2510	0.2428	0.0907
hrrnum=368	0.350***	0.522***	0.380***	-0.248**	-0.469***	-0.343*	0.466***	-0.345***	-0.194***
	0.0586	0.0600	0.0956	0.0811	0.1214	0.1415	0.0768	0.0841	0.0272
hrrnum=369	-0.085	-0.084	0.564*	-0.386	-0.522	-1.093	-0.009	0.210	-0.090
	0.1567	0.1544	0.2728	0.1987	0.4138	0.6022	0.1781	0.2183	0.0720
hrrnum=370	-0.327	-0.065	0.470	0.171	-0.519	0.026	-0.303	0.415	0.010
	0.3101	0.3061	0.4941	0.4815	1.0178	0.7676	0.3346	0.4106	0.1429
hrrnum=371	0.117	0.310***	0.273	-0.191	-0.154	-0.083	0.246*	0.052	-0.014
	0.0919	0.0939	0.1500	0.1246	0.1755	0.2010	0.1130	0.1138	0.0391
hrrnum=373	-0.006	-0.071	0.032	0.141	-0.285	-0.288	-0.189*	-0.339**	-0.080*
	0.0698	0.0704	0.1118	0.1005	0.1487	0.1751	0.0891	0.1036	0.0325
hrrnum=374	0.138	0.147	0.186	-0.054	0.052	0.156	0.075	-0.166	-0.123***
	0.0767	0.0775	0.1268	0.1090	0.1424	0.1618	0.0989	0.1033	0.0351
hrrnum=375	0.016	0.075	0.228*	-0.149	-0.102	0.020	0.135	0.068	-0.098**
	0.0710	0.0718	0.1147	0.1003	0.1351	0.1557	0.0891	0.0910	0.0329
hrrnum=376	-0.005	-0.024	0.513***	-0.017	-0.038	0.165	0.040	0.006	0.103***
	0.0613	0.0615	0.1007	0.0872	0.1204	0.1333	0.0790	0.0827	0.0289
hrrnum=377	0.100	0.077	0.456***	-0.012	0.055	-0.287	0.107	-0.114	0.069*
	0.0675	0.0681	0.1139	0.1003	0.1258	0.1549	0.0844	0.0908	0.0307
hrrnum=379	-0.177*	-0.185*	0.469***	0.113	-0.071	0.109	-0.026	0.065	0.020
	0.0845	0.0841	0.1402	0.1194	0.1637	0.1838	0.1060	0.1102	0.0388
hrrnum=380	-0.062	-0.041	0.266**	-0.184*	0.169	0.362**	-0.027	0.101	0.097***
	0.0561	0.0565	0.0892	0.0789	0.1071	0.1227	0.0717	0.0739	0.0262
hrrnum=382	-0.128	-0.171	0.734*	0.428	-0.077	0.163	-0.628**	-0.118	-0.004
	0.2086	0.2063	0.3473	0.3089	0.5109	0.4854	0.2311	0.3622	0.1092
hrrnum=383	0.021	-0.010	0.401**	0.412***	-0.084	-0.333	-0.285**	-0.282**	-0.246***
	0.0758	0.0766	0.1280	0.1214	0.1484	0.1842	0.0912	0.1073	0.0346
hrrnum=385	0.008	0.007	0.276**	-0.043	-0.121	0.317*	-0.163*	-0.139	0.127***
	0.0637	0.0643	0.1045	0.0903	0.1297	0.1468	0.0789	0.0885	0.0290
hrrnum=386	0.206*	0.205	0.541**	-0.026	-0.280	-0.288	0.107	-0.064	0.106*
	0.1034	0.1047	0.1823	0.1474	0.2148	0.2640	0.1332	0.1512	0.0515
hrrnum=388	-0.164	-0.198	0.112	-0.082	-0.051	0.715***	0.081	0.054	0.149**
	0.1077	0.1067	0.1617	0.1443	0.1958	0.2018	0.1371	0.1316	0.0469
hrrnum=390	-0.121	0.002	-0.226	-0.216	-0.559	0.023	-0.239	-0.287	-0.065
	0.1498	0.1500	0.2234	0.2061	0.4351	0.4097	0.1835	0.2907	0.0786
hrrnum=391	-0.022	-0.079	0.318***	0.295***	-0.310**	0.125	-0.171*	-0.164*	-0.028
	0.0549	0.0553	0.0878	0.0793	0.1096	0.1245	0.0701	0.0752	0.0256
hrrnum=393	-0.447***	-0.330***	-0.065	-0.461***	-0.296	0.043	-0.311**	-0.097	-0.115**
	0.0872	0.0853	0.1311	0.1122	0.1850	0.2032	0.1113	0.1198	0.0407
hrrnum=394	0.059	0.048	0.325**	0.121	-0.210	0.076	-0.080	-0.316***	0.093**
	0.0621	0.0628	0.1024	0.0901	0.1264	0.1427	0.0782	0.0882	0.0288

© 2022 Bakre S et al. *JAMA Health Forum.*

hrrnum=396	0.404***	0.482***	-0.250*	0.524***	-0.760***	-0.673***	0.376***	-0.460***	-0.667***
	0.0657	0.0677	0.1002	0.1038	0.1446	0.1651	0.0952	0.0988	0.0343
hrrnum=397	-0.151**	-0.219***	0.307***	0.126	-0.226*	0.088	0.205**	-0.119	-0.101***
	0.0536	0.0539	0.0844	0.0759	0.1057	0.1223	0.0699	0.0728	0.0253
hrrnum=399	0.080	0.116	0.935	0.062	-0.798	0.000	-0.275	-0.357	-0.056
	0.3182	0.3131	0.6286	0.4600	0.7452	.	0.3316	0.4309	0.1345
hrrnum=400	-0.312***	-0.285***	0.142	-0.107	-0.028	-0.063	-0.222*	-0.207*	-0.014
	0.0777	0.0776	0.1196	0.1042	0.1379	0.1619	0.0946	0.0976	0.0335
hrrnum=402	0.459***	0.423***	0.349**	0.805***	-0.543**	-0.337	0.478***	-0.223	-0.629***
	0.0812	0.0831	0.1314	0.1454	0.1696	0.1944	0.1216	0.1205	0.0456
hrrnum=406	-0.647***	-0.612***	-0.270	-0.343*	-0.278	0.230	-0.312*	-0.103	-0.035
	0.1051	0.1034	0.1439	0.1352	0.2142	0.2007	0.1393	0.1405	0.0466
hrrnum=411	0.145	0.532*	0.054	-0.621*	-0.316	0.773	-1.320***	-0.339	-0.118
	0.2571	0.2460	0.3436	0.2759	0.5249	0.4297	0.3602	0.3640	0.1188
hrrnum=412	-0.185**	-0.217***	0.026	0.028	-0.363**	-0.274	-0.303***	-0.240**	-0.169***
	0.0583	0.0585	0.0916	0.0832	0.1236	0.1468	0.0750	0.0855	0.0283
hrrnum=413	-0.203	-0.246	0.266	0.268	-0.368	0.376	-0.274	0.186	0.207**
	0.1340	0.1341	0.2171	0.1975	0.3286	0.2926	0.1677	0.1855	0.0637
hrrnum=416	0.566***	0.507***	0.536**	0.750***	-0.008	-0.744**	0.302*	-0.445**	-0.001
	0.1013	0.1041	0.1710	0.1677	0.1879	0.2847	0.1265	0.1415	0.0439
hrrnum=417	-0.457	-0.363	0.597	0.805	0.228	0.116	-0.335	0.063	0.270*
	0.2907	0.2799	0.4713	0.5244	0.5079	0.7105	0.3149	0.3572	0.1271
hrrnum=418	0.364*	0.241	0.720*	0.655*	0.257	-0.228	0.006	-0.172	0.059
	0.1423	0.1460	0.2882	0.2548	0.2688	0.3833	0.1769	0.2006	0.0624
hrrnum=420	0.313**	0.329***	0.275	-0.103	-0.584**	-0.437	-0.093	-0.284*	-0.270***
	0.0989	0.0992	0.1588	0.1398	0.1945	0.2439	0.1160	0.1446	0.0446
hrrnum=421	-0.487***	-0.194*	-0.193	-0.735***	-0.141	-0.614	-0.365***	-0.236	-0.009
	0.0932	0.0917	0.1460	0.1176	0.2090	0.3296	0.1106	0.1507	0.0436
hrrnum=422	-0.162*	0.034	0.201	-0.736***	-0.716***	-0.520*	-0.418***	-0.212*	0.029
	0.0762	0.0773	0.1278	0.1004	0.1850	0.2446	0.0915	0.1075	0.0343
hrrnum=423	-0.378***	-0.145*	0.046	-0.875***	-0.354*	-0.585**	-0.290***	-0.060	0.079*
	0.0712	0.0709	0.1131	0.0912	0.1555	0.2112	0.0842	0.0986	0.0318
hrrnum=424	0.061	0.238***	0.593***	-0.365***	0.118	0.169	0.371***	-0.053	0.199***
	0.0588	0.0596	0.0966	0.0801	0.1099	0.1271	0.0750	0.0774	0.0265
hrrnum=426	0.003	0.022	0.323***	-0.125	0.052	0.156	-0.201**	-0.046	0.115***
	0.0586	0.0592	0.0956	0.0828	0.1149	0.1391	0.0728	0.0803	0.0268
hrrnum=427	0.263***	0.446***	0.262*	-0.290***	0.072	-0.526**	0.281***	-0.272**	0.038
	0.0639	0.0655	0.1031	0.0875	0.1196	0.1600	0.0823	0.0886	0.0290
hrrnum=428	-0.019	0.030	0.039	-0.198	-0.006	-0.685	0.717***	-0.021	-0.016
	0.1329	0.1335	0.2022	0.1763	0.2939	0.4320	0.1976	0.1949	0.0629
hrrnum=429	0.192**	0.271***	0.127	0.073	-0.005	-0.187	0.168	-0.231*	-0.035

© 2022 Bakre S et al. *JAMA Health Forum.*

	0.0746	0.0770	0.1237	0.1090	0.1421	0.1802	0.1009	0.1061	0.0331
hrrnum=430	0.521***	0.613***	0.436***	0.174*	-0.142	-0.335*	0.652***	-0.281**	0.036
	0.0610	0.0628	0.1007	0.0872	0.1197	0.1467	0.0843	0.0873	0.0284
hrrnum=431	0.608***	0.672***	0.586***	0.214*	-0.017	-0.048	0.937***	-0.139	0.036
	0.0584	0.0600	0.0970	0.0831	0.1122	0.1356	0.0819	0.0789	0.0267
hrrnum=432	0.033	0.003	0.425***	0.148	-0.015	-0.617***	0.880***	0.019	-0.079**
	0.0607	0.0612	0.0989	0.0877	0.1178	0.1543	0.0841	0.0812	0.0282
hrrnum=435	-0.147*	-0.145*	0.422***	-0.021	0.150	0.251	0.052	0.057	0.164***
	0.0664	0.0665	0.1125	0.0952	0.1270	0.1433	0.0860	0.0899	0.0305
hrrnum=437	-0.355***	-0.316**	0.302	-0.537***	-0.368	-0.546	0.503***	-0.059	0.044
	0.1015	0.1020	0.1826	0.1318	0.2080	0.2800	0.1333	0.1372	0.0440
hrrnum=438	-0.440***	-0.264**	0.008	-0.517***	0.141	-0.217	0.165	-0.240	0.192***
	0.0919	0.0909	0.1430	0.1145	0.1605	0.2307	0.1091	0.1245	0.0377
hrrnum=439	-0.183**	-0.084	0.428***	-0.393***	-0.042	-0.474**	0.207**	-0.183*	0.017
	0.0626	0.0633	0.1038	0.0849	0.1165	0.1584	0.0767	0.0837	0.0273
hrrnum=440	-0.264**	-0.171*	0.068	-0.572***	-0.250	-0.563*	0.266**	-0.232*	0.158***
	0.0862	0.0863	0.1365	0.1075	0.1569	0.2210	0.1032	0.1149	0.0340
hrrnum=441	-0.332***	-0.233***	-0.123	-0.650***	-0.099	-0.182	-0.400***	-0.120	-0.082**
	0.0689	0.0691	0.1030	0.0895	0.1338	0.1643	0.0808	0.0940	0.0306
hrrnum=442	-0.507	-0.356	0.534	-0.100	-1.262	0.012	0.662	-0.681	0.258*
	0.2907	0.2739	0.5010	0.3604	1.0163	0.7360	0.3987	0.5285	0.1205
hrrnum=443	-0.216*	-0.059	-0.127	-0.451***	0.089	-0.247	0.223	-0.054	0.064
	0.0987	0.0979	0.1534	0.1271	0.1817	0.2158	0.1364	0.1260	0.0480
hrrnum=444	0.897***	1.102***	0.928***	0.156	0.107	0.068	1.064***	0.161	0.233***
	0.1040	0.1153	0.1966	0.1418	0.1815	0.1908	0.1577	0.1277	0.0472
hrrnum=445	-0.048	0.117	0.214	-0.435***	-0.100	0.390*	1.473***	-0.159	0.320***
	0.0860	0.0864	0.1364	0.1120	0.1787	0.1771	0.1464	0.1288	0.0401
hrrnum=446	0.205	0.224	-0.032	0.206	0.432	0.000	0.163	0.179	-0.009
	0.2817	0.2829	0.3976	0.4166	0.4507	.	0.4321	0.3432	0.1174
hrrnum=447	0.083	0.066	1.032*	0.182	-0.359	-1.232	1.125**	-0.296	0.196*
	0.2488	0.2511	0.4524	0.3289	0.5124	1.0162	0.3926	0.3232	0.0980
hrrnum=448	-0.084	0.136	0.263	-0.430	-0.289	0.678	0.134	0.181	0.354***
	0.2220	0.2234	0.3114	0.2712	0.3941	0.3703	0.3210	0.2485	0.0910
hrrnum=449	0.196**	0.128*	0.933***	0.196*	0.005	0.047	0.356***	-0.117	0.162***
	0.0622	0.0632	0.1129	0.0908	0.1203	0.1488	0.0776	0.0838	0.0277
hrrnum=450	0.350***	0.294***	1.040***	0.349**	-0.088	0.330*	0.551***	-0.106	0.116***
	0.0746	0.0761	0.1411	0.1111	0.1363	0.1569	0.0949	0.0939	0.0318
hrrnum=451	-0.143*	-0.159**	0.262**	-0.123	0.151	0.259*	0.341***	0.019	0.145***
	0.0589	0.0593	0.0938	0.0828	0.1103	0.1304	0.0765	0.0763	0.0269
hrrnum=452	0.560	0.425	1.152	0.597	-0.346	-0.006	0.297	-0.950	0.165
	0.3193	0.3284	0.7595	0.5277	0.6261	0.7251	0.4047	0.5291	0.1313

© 2022 Bakre S et al. *JAMA Health Forum.*

hrrnum=456	0.633***	0.489***	1.580***	0.760***	-0.207	-0.319	0.987***	0.103	0.048
	0.1034	0.1063	0.2490	0.1727	0.1847	0.2493	0.1384	0.1178	0.0408
hrrnum=457	-0.712	-0.693	-0.233	-1.830**	0.000	0.359	-0.988	-0.508	0.250
	0.7270	0.7351	0.7623	0.5933	.	0.7434	0.5340	0.6228	0.1716
hrrnum=999	0.354	0.845	-0.868	-0.304	-0.114	0.000	0.056	-0.089	-0.654**
	0.4149	0.5092	0.5393	0.5610	0.7951	.	0.5119	0.6763	0.2447
HIV/AIDS	0.013	0.046	0.081	0.217*	0.299*	-0.099	0.534***	0.059	0.457***
	0.0663	0.0654	0.1032	0.0947	0.1447	0.1673	0.1219	0.0954	0.0379
Septicemia, Sepsis, Systemic Inflammatory Response Syndrome/Shock	-0.167***	-0.110***	-0.070*	-0.211***	-0.186***	0.057*	-0.321***	0.152***	0.247***
	0.0211	0.0211	0.0300	0.0246	0.0241	0.0274	0.0357	0.0163	0.0095
Opportunistic Infections	0.034	0.138*	-0.232**	-0.136*	-0.075	0.429***	0.105	0.129**	0.186***
	0.0540	0.0547	0.0742	0.0662	0.0690	0.0522	0.0709	0.0433	0.0225
Metastatic Cancer and Acute Leukemia	-0.607***	-0.424***	-0.967***	-1.044***	0.127**	-0.038	-0.573***	0.524***	0.622***
	0.0265	0.0254	0.0310	0.0271	0.0404	0.0453	0.0326	0.0223	0.0108
Lung and Other Severe Cancers	-0.162***	-0.078***	-0.395***	-0.449***	0.093**	0.285***	0.085*	0.267***	0.307***
	0.0227	0.0229	0.0323	0.0275	0.0324	0.0280	0.0357	0.0207	0.0095
Lymphoma and Other Cancers	-0.058**	0.031	-0.314***	-0.248***	0.122***	-0.056	0.311***	0.156***	0.135***
	0.0223	0.0229	0.0339	0.0294	0.0340	0.0443	0.0321	0.0236	0.0090
Colorectal, Bladder, and Other Cancers	-0.009	0.019	-0.084**	-0.086***	-0.129***	-0.049	0.331***	0.109***	0.068***
	0.0181	0.0184	0.0296	0.0251	0.0291	0.0334	0.0334	0.0194	0.0074
Breast, Prostate, and Other Cancers and Tumors	0.141***	0.184***	0.043*	0.063***	-0.139***	-0.183***	0.718***	-0.030*	-0.019***
	0.0106	0.0110	0.0186	0.0158	0.0198	0.0250	0.0182	0.0140	0.0044
Diabetes with Acute Complications	0.796***	0.546***	1.598***	0.398***	0.500***	-0.031	-0.234***	0.333***	0.305***
	0.0301	0.0308	0.0541	0.0408	0.0593	0.0825	0.0663	0.0434	0.0216
Diabetes with Chronic Complications	0.943***	0.708***	1.664***	0.442***	0.490***	-0.039*	-0.055***	0.175***	0.182***
	0.0082	0.0082	0.0136	0.0112	0.0135	0.0180	0.0139	0.0102	0.0039
Diabetes without Complication	0.547***	0.283***	1.290***	0.416***	0.253***	-0.002	-0.076***	0.095***	0.038***
	0.0072	0.0071	0.0104	0.0098	0.0130	0.0153	0.0095	0.0094	0.0032
Protein-Calorie Malnutrition	-0.255***	-0.179***	-0.361***	-0.278***	-0.146***	0.099**	-0.339***	0.097***	0.152***
	0.0361	0.0348	0.0432	0.0388	0.0354	0.0373	0.0474	0.0230	0.0132
Morbid Obesity	-0.011	-0.001	0.075***	-0.043**	0.419***	0.155***	0.004	0.051**	0.172***

© 2022 Bakre S et al. *JAMA Health Forum.*

	0.0111	0.0114	0.0198	0.0154	0.0227	0.0269	0.0176	0.0179	0.0075
Other Significant Endocrine and Metabolic Disorders	0.097***	0.133***	0.010	0.014	0.082***	0.008	0.159***	0.105***	0.104***
	0.0135	0.0140	0.0225	0.0187	0.0210	0.0269	0.0216	0.0155	0.0064
End-Stage Liver Disease	-0.289***	-0.146***	-0.268***	-0.668***	0.217**	-0.403***	-0.273***	0.538***	0.533***
	0.0368	0.0368	0.0552	0.0424	0.0681	0.0997	0.0620	0.0404	0.0225
Cirrhosis of Liver	-0.119***	-0.038	-0.252***	-0.338***	0.314***	-0.114	-0.004	0.268***	0.285***
	0.0332	0.0337	0.0500	0.0415	0.0562	0.0737	0.0535	0.0400	0.0184
Chronic Hepatitis	0.040	0.070	-0.058	-0.020	0.130	-0.161	0.201***	0.162**	-0.049*
	0.0393	0.0399	0.0633	0.0550	0.0879	0.0958	0.0562	0.0569	0.0216
Intestinal Obstruction/Perforation	-0.066**	-0.033	-0.171***	-0.109***	-0.194***	-0.080*	0.067	0.137***	0.272***
	0.0250	0.0250	0.0354	0.0309	0.0327	0.0366	0.0365	0.0199	0.0102
Chronic Pancreatitis	-0.086	-0.047	-0.023	-0.229***	-0.187	-0.026	-0.013	0.271***	0.404***
	0.0536	0.0536	0.0878	0.0685	0.1036	0.1029	0.0935	0.0574	0.0287
Inflammatory Bowel Disease	0.025	0.099***	-0.164***	-0.081*	-0.217***	-0.078	0.236***	0.144***	0.182***
	0.0285	0.0294	0.0450	0.0397	0.0541	0.0554	0.0376	0.0303	0.0121
Bone/Joint/Muscle Infections/Necrosis	-0.124***	-0.079**	-0.151***	-0.179***	0.025	-0.104	-0.088	0.016	0.068***
	0.0271	0.0276	0.0406	0.0338	0.0406	0.0554	0.0449	0.0272	0.0138
Rheumatoid Arthritis and Inflammatory Connective Tissue Disease	0.053***	0.194***	-0.243***	-0.173***	-0.015	-0.030	0.108***	0.097***	0.184***
	0.0119	0.0124	0.0190	0.0164	0.0198	0.0220	0.0147	0.0128	0.0050
Severe Hematological Disorders	-0.088*	0.055	-0.228***	-0.252***	0.389***	-0.041	-0.000	0.293***	0.319***
	0.0414	0.0425	0.0579	0.0502	0.0422	0.0637	0.0696	0.0308	0.0160
Disorders of Immunity	-0.066**	0.008	-0.271***	-0.231***	0.074*	0.120**	0.062*	0.218***	0.234***
	0.0235	0.0237	0.0326	0.0283	0.0348	0.0390	0.0312	0.0221	0.0105
Coagulation Defects and Other Specified Hematological Disorders	0.021	0.048***	-0.069**	-0.003	0.072***	-0.100***	0.171***	0.071***	0.069***
	0.0141	0.0144	0.0222	0.0191	0.0182	0.0246	0.0227	0.0138	0.0059
Drug/Alcohol Psychosis	-0.430***	-0.294***	-0.474***	-0.318***	0.048	0.489***	-0.501***	0.215**	0.521***
	0.0775	0.0750	0.0990	0.0862	0.1233	0.0899	0.0975	0.0677	0.0345
Drug/Alcohol Dependence	-0.212***	-0.221***	-0.210**	-0.130*	0.066	0.324***	-0.251***	0.179***	0.369***
	0.0437	0.0430	0.0662	0.0556	0.0826	0.0669	0.0578	0.0465	0.0217
Schizophrenia	-0.129***	0.043	-0.164**	-0.292***	-0.354***	0.161*	-0.343***	0.280***	0.550***
	0.0362	0.0359	0.0504	0.0417	0.0903	0.0656	0.0464	0.0426	0.0200
Major Depressive, Bipolar,	-0.121***	-0.038**	-0.145***	-0.170***	-0.065**	0.083***	-0.041*	0.088***	0.334***

© 2022 Bakre S et al. *JAMA Health Forum.*

and Paranoid Disorders									
	0.0134	0.0137	0.0215	0.0176	0.0239	0.0242	0.0160	0.0151	0.0060
Quadriplegia	-0.271**	-0.118	-0.193	-0.290**	-0.164	-0.499**	-0.777***	0.091	0.439***
	0.0987	0.0944	0.1204	0.1066	0.1459	0.1892	0.1421	0.0758	0.0443
Paraplegia	-0.412***	-0.286***	-0.415***	-0.370***	-0.082	-0.103	-0.377***	0.327***	0.466***
	0.0831	0.0792	0.1012	0.0916	0.1439	0.1684	0.1130	0.0702	0.0393
Spinal Cord Disorders/Injuries	-0.026	0.052	-0.179**	-0.189***	-0.062	-0.098	-0.064	0.087**	0.246***
	0.0373	0.0382	0.0546	0.0470	0.0535	0.0649	0.0521	0.0334	0.0153
Amyotrophic Lateral Sclerosis and Other Motor Neuron Disease	-0.454**	-0.309*	-0.257	-0.449**	-0.224	0.066	-0.806***	0.051	0.574***
	0.1511	0.1461	0.2128	0.1672	0.2817	0.2433	0.1725	0.1348	0.0586
Cerebral Palsy	0.074	0.217*	-0.042	0.109	0.230	-0.077	-0.240*	0.188	0.429***
	0.1076	0.1084	0.1459	0.1335	0.1959	0.2159	0.1159	0.1040	0.0466
Myasthenia Gravis/Myoneural Disorders, Inflammatory and Toxic Neuropathy	0.005	0.091***	-0.152***	-0.110***	-0.016	0.074	0.094*	0.068**	0.152***
	0.0234	0.0243	0.0373	0.0309	0.0401	0.0449	0.0390	0.0255	0.0109
Muscular Dystrophy	-0.068	0.091	-0.242	-0.044	-0.086	-0.215	-0.165	0.280	0.349***
	0.1486	0.1513	0.2149	0.1996	0.2960	0.3180	0.1937	0.1537	0.0660
Multiple Sclerosis	-0.051	0.146**	-0.262***	-0.330***	-0.006	0.152	-0.215***	0.209***	0.381***
	0.0469	0.0483	0.0681	0.0577	0.1065	0.0971	0.0409	0.0516	0.0208
Parkinson's and Huntington's Diseases	-0.122***	0.039	-0.342***	-0.340***	-0.150***	0.001	-0.301***	0.139***	0.530***
	0.0278	0.0284	0.0402	0.0343	0.0375	0.0449	0.0444	0.0217	0.0095
Seizure Disorders and Convulsions	-0.115***	-0.006	-0.287***	-0.210***	-0.089**	-0.019	-0.174***	0.120***	0.412***
	0.0210	0.0212	0.0293	0.0256	0.0319	0.0352	0.0266	0.0193	0.0087
Coma, Brain Compression/Anoxic Damage	-0.088	-0.004	-0.102	-0.153*	-0.248*	-0.203	-0.141	0.066	0.211***
	0.0664	0.0652	0.0851	0.0757	0.0985	0.1147	0.0923	0.0580	0.0307
Respirator Dependence/Tracheostomy Status	-0.252***	-0.129*	-0.153*	-0.362***	0.191**	0.580***	-0.180	0.123*	0.056
	0.0638	0.0602	0.0756	0.0643	0.0737	0.0702	0.0967	0.0504	0.0328
Respiratory Arrest	0.201	0.055	0.147	0.316	0.433**	0.750***	-0.114	0.164	0.103
	0.1748	0.1738	0.2242	0.2215	0.1478	0.1690	0.2367	0.1346	0.0858
Cardio-Respiratory Failure	-0.187***	-0.148***	-0.259***	-0.275***	0.519***	0.947***	-0.284***	0.234***	0.239***

© 2022 Bakre S et al. *JAMA Health Forum.*

and Shock									
	0.0182	0.0181	0.0248	0.0215	0.0172	0.0182	0.0290	0.0142	0.0082
Congestive Heart Failure	-0.173***	-0.140***	-0.222***	-0.205***	1.549***	0.398***	-0.261***	0.273***	0.295***
	0.0094	0.0095	0.0148	0.0123	0.0128	0.0155	0.0164	0.0091	0.0039
Acute Myocardial Infarction	-0.139***	-0.163***	-0.206***	0.010	0.365***	-0.094*	-0.315***	0.142***	0.341***
	0.0255	0.0253	0.0366	0.0322	0.0244	0.0368	0.0471	0.0216	0.0118
Unstable Angina and Other Acute Ischemic Heart Disease	0.022	-0.005	-0.039	0.137***	0.261***	0.061*	-0.017	0.083***	0.313***
	0.0186	0.0189	0.0300	0.0263	0.0232	0.0307	0.0360	0.0191	0.0089
Angina Pectoris	0.091***	0.092***	-0.037	0.159***	0.063*	-0.041	0.107***	-0.019	0.206***
	0.0167	0.0171	0.0280	0.0251	0.0252	0.0316	0.0305	0.0197	0.0080
Specified Heart Arrhythmias	-0.053***	-0.013	-0.203***	-0.079***	0.738***	-0.025	-0.006	0.147***	0.265***
	0.0088	0.0089	0.0139	0.0119	0.0118	0.0151	0.0154	0.0086	0.0034
Cerebral Hemorrhage	-0.109*	-0.083	-0.039	-0.124*	-0.124*	-0.110	-0.219**	0.014	0.187***
	0.0452	0.0453	0.0663	0.0563	0.0574	0.0765	0.0696	0.0384	0.0175
Ischemic or Unspecified Stroke	-0.117***	-0.119***	-0.095***	-0.103***	0.034	-0.055*	-0.215***	0.074***	0.307***
	0.0156	0.0157	0.0244	0.0206	0.0203	0.0273	0.0264	0.0146	0.0065
Hemiplegia/Hemiparesis	-0.211***	-0.127***	-0.169***	-0.129***	-0.040	-0.021	-0.460***	0.018	0.248***
	0.0284	0.0283	0.0400	0.0344	0.0391	0.0516	0.0462	0.0259	0.0127
Monoplegia, Other Paralytic Syndromes	-0.213*	-0.166	-0.305**	-0.386***	0.073	0.029	-0.416***	0.145	0.177***
	0.0870	0.0876	0.1153	0.1025	0.1174	0.1454	0.1231	0.0786	0.0372
Atherosclerosis of the Extremities with Ulceration or Gangrene	-0.273***	-0.224***	-0.448***	-0.306***	0.282***	-0.114	-0.513***	0.276***	0.343***
	0.0362	0.0368	0.0523	0.0437	0.0449	0.0696	0.0807	0.0330	0.0190
Vascular Disease with Complications	-0.141***	-0.097***	-0.251***	-0.128***	0.094***	0.069*	-0.108***	0.093***	0.218***
	0.0186	0.0186	0.0277	0.0239	0.0230	0.0277	0.0304	0.0171	0.0080
Vascular Disease	0.015	0.044***	-0.142***	0.006	0.102***	0.036*	0.002	0.076***	0.145***
	0.0080	0.0082	0.0133	0.0113	0.0121	0.0145	0.0139	0.0087	0.0034
Cystic Fibrosis	-0.552**	-0.346	-0.531	-0.462*	0.513	0.943	0.484	-0.047	0.052
	0.1919	0.1893	0.2722	0.2351	0.2831	0.5065	0.2851	0.2759	0.0976
Chronic Obstructive Pulmonary Disease	-0.271***	-0.236***	-0.330***	-0.243***	0.413***	2.737***	-0.332***	0.291***	0.382***
	0.0086	0.0086	0.0132	0.0113	0.0120	0.0152	0.0124	0.0087	0.0036
Fibrosis of Lung and Other Chronic Lung Disorders	0.048	0.113***	-0.148***	-0.093**	0.238***	0.908***	0.232***	0.119***	0.196***
	0.0251	0.0261	0.0400	0.0339	0.0336	0.0560	0.0372	0.0249	0.0100

© 2022 Bakre S et al. *JAMA Health Forum.*

Aspiration and Specified Bacterial Pneumonias	-0.059	0.041	-0.149[**]	-0.148[***]	-0.104[**]	0.297[***]	-0.008	0.119[***]	0.185[***]
	0.0395	0.0387	0.0496	0.0432	0.0342	0.0333	0.0639	0.0242	0.0155
Pneumococcal Pneumonia, Empyema, Lung Abscess	-0.044	-0.058	-0.150[*]	-0.105	0.100	0.305[***]	-0.084	0.147[***]	0.125[***]
	0.0558	0.0550	0.0754	0.0671	0.0566	0.0526	0.0837	0.0427	0.0239
Proliferative Diabetic Retinopathy and Vitreous Hemorrhage	0.838[***]	1.411[***]	0.067	-0.057	0.474[***]	-0.037	-0.133[**]	0.140[***]	0.182[***]
	0.0239	0.0315	0.0417	0.0294	0.0405	0.0734	0.0454	0.0348	0.0147
Exudative Macular Degeneration	1.146[***]	2.238[***]	-0.017	0.008	0.108[***]	0.160[***]	0.001	-0.002	0.047[***]
	0.0403	0.0683	0.0576	0.0481	0.0261	0.0345	0.0592	0.0213	0.0080
Dialysis Status	-0.066	0.080	-0.114	-0.268[**]	0.543[***]	-0.126	-0.141	0.250[***]	0.243[***]
	0.0763	0.0780	0.1094	0.0879	0.0907	0.1460	0.1379	0.0724	0.0425
Acute Renal Failure	-0.206[***]	-0.162[***]	-0.241[***]	-0.273[***]	0.500[***]	-0.009	-0.365[***]	0.236[***]	0.306[***]
	0.0149	0.0150	0.0223	0.0183	0.0160	0.0230	0.0281	0.0126	0.0069
Chronic Kidney Disease, Stage 5	-0.026	0.043	-0.230[***]	-0.184[***]	0.408[***]	-0.048	-0.010	0.172[***]	0.159[***]
	0.0365	0.0380	0.0587	0.0490	0.0483	0.0755	0.0748	0.0398	0.0181
Chronic Kidney Disease, Severe (Stage 4)	-0.151[***]	-0.133[***]	-0.231[***]	-0.239[***]	0.719[***]	0.089	-0.355[***]	0.259[***]	0.268[***]
	0.0286	0.0297	0.0476	0.0371	0.0294	0.0524	0.0587	0.0265	0.0126
Pressure Ulcer of Skin with Necrosis Through to Muscle, Tendon, or Bone	-0.369[**]	-0.246[*]	-0.177	-0.345[**]	-0.414[**]	-0.680[***]	-0.923[***]	0.217[**]	0.181[***]
	0.1129	0.1041	0.1237	0.1074	0.1400	0.2014	0.2120	0.0720	0.0509
Pressure Ulcer of Skin with Full Thickness Skin Loss	-0.344[***]	-0.246[***]	-0.364[***]	-0.417[***]	-0.012	-0.348[***]	-0.608[***]	0.048	0.214[***]
	0.0677	0.0654	0.0813	0.0697	0.0664	0.1016	0.1247	0.0460	0.0292
Chronic Ulcer of Skin, Except Pressure	-0.171[***]	-0.102[***]	-0.286[***]	-0.288[***]	0.211[***]	-0.024	-0.323[***]	0.102[***]	0.242[***]
	0.0175	0.0181	0.0277	0.0223	0.0227	0.0331	0.0329	0.0174	0.0082
Severe Skin Burn or Condition	0.109	0.184	-0.040	-0.201	0.198	0.195	-0.126	-0.294	0.086
	0.2302	0.2388	0.3254	0.2956	0.3227	0.3844	0.3514	0.2456	0.1121
Severe Head Injury	-0.490	-0.384	0.501	-0.256	-0.642	-1.268	0.105	-0.030	0.134
	0.2735	0.2790	0.4309	0.2972	0.4621	0.7197	0.3459	0.2453	0.1088
Major Head Injury	-0.006	0.062	-0.230[***]	-0.099	0.005	-0.100	-0.063	0.012	0.225[***]
	0.0461	0.0473	0.0676	0.0589	0.0559	0.0749	0.0712	0.0384	0.0175
Vertebral Fractures without	-0.173[***]	-0.073[*]	-0.330[***]	-0.299[***]	0.078[**]	0.271[***]	-0.172[***]	0.149[***]	0.314[***]

© 2022 Bakre S et al. *JAMA Health Forum.*

Spinal Cord Injury									
	0.0311	0.0314	0.0434	0.0380	0.0299	0.0321	0.0402	0.0204	0.0095
Hip Fracture/Dislocation	-0.208***	-0.199***	-0.227***	-0.232***	-0.080*	-0.135**	-0.288***	-0.018	0.168***
	0.0358	0.0360	0.0505	0.0435	0.0318	0.0416	0.0465	0.0222	0.0103
Traumatic Amputations and Complications	0.050	0.060	-0.013	-0.037	-0.126	-0.080	-0.031	0.026	0.067**
	0.0474	0.0476	0.0675	0.0576	0.0651	0.0786	0.0820	0.0444	0.0230
Complications of Specified Implanted Device or Graft	-0.014	0.010	-0.176***	-0.088**	0.042	-0.168***	0.088**	0.120***	0.269***
	0.0226	0.0229	0.0333	0.0287	0.0322	0.0422	0.0331	0.0201	0.0100
Major Organ Transplant or Replacement Status	0.066	0.107*	-0.040	0.126*	0.179*	-0.440**	0.252**	0.288***	0.286***
	0.0435	0.0439	0.0637	0.0562	0.0863	0.1350	0.0824	0.0533	0.0275
Artificial Openings for Feeding or Elimination	-0.275***	-0.175***	-0.217***	-0.312***	-0.248***	-0.285***	-0.260***	0.193***	0.385***
	0.0383	0.0371	0.0480	0.0413	0.0517	0.0576	0.0534	0.0272	0.0156
Amputation Status, Lower Limb/Amputation Complications	-0.274***	-0.216***	-0.240***	-0.286***	0.231**	0.170	-0.377***	0.283***	0.299***
	0.0452	0.0458	0.0655	0.0533	0.0739	0.0974	0.1035	0.0506	0.0274
Constant	-3.312***	-3.649***	1.162***	0.848***	-9.974***	-6.786***	-2.983***	-2.988***	-4.454***
	0.0865	0.0875	0.1409	0.1220	0.1187	0.1383	0.2188	0.0818	0.0284
N	616,334	616,334	616,310	616,334	3813931	3813708	510,905	610,453	3815009
aic	811454	792106	362717	474422	384103	280144	540773	513213	4331175
bic	816088	796740	367340	479057	389391	285432	545308	517843	4336555
C	0.6496	0.6421	0.7304	0.6679	0.8687	0.8791	0.6679	0.6438	0.7043

* $p < 0.05$, ** $p < 0.01$, *** $p < 0.001$

© 2022 Bakre S et al. *JAMA Health Forum.*

eTable 4. Adjusted risk differences on sensitivity analyses†

Quality Measure	Top vs. Bottom Quartile of Overall Racial and Ethnic Minority Proportion Distribution (1,611,760 beneficiary-years)		High vs. Low Racial and Ethnic Minority ACOs with 0% to 60% Out-of-Network Primary Care (3,831,132 beneficiary-years)		High vs. Low Racial and Ethnic Minority ACOs with 0% to 90% Out-of-Network Primary Care (3,881,540 beneficiary-years)		High vs. Low Racial and Ethnic Minority ACOs, Including State Fixed Effects (3,955,951 beneficiary-years)	
	Adjusted Risk Difference	95% Confidence Interval	Adjusted Risk Difference	95% Confidence Interval	Adjusted Risk Difference	95% Confidence Interval	Adjusted Risk Difference	95% Confidence Interval
Preventive services								
All diabetes tests	-1.63*	-2.19 to -1.08	-2.18*	-2.61 to -1.75	-2.21*	-2.63 to -1.79	-2.19*	-2.61 to -1.78
Diabetic retinal examinations	-0.99*	-1.53 to -0.45	-1.74*	-2.16 to -1.32	-1.85*	-2.27 to -1.44	-1.88*	-2.28 to -1.47
Glycated hemoglobin testing	-1.07*	-1.39 to -0.75	-1.08*	-1.34 to -0.81	-1.07*	-1.33 to -0.82	-0.96*	-1.21 to -0.71
LDL cholesterol testing	-1.01*	-1.39 to -0.62	-1.03*	-1.35 to -0.72	-0.87*	-1.18 to -0.56	-0.74*	-1.04 to -0.43
Mammography	-0.52	-1.05 to 0.01	-1.33*	-1.77 to -0.89	-1.57*	-2.01 to -1.14	-1.55*	-1.98 to -1.13
Utilization								
CHF hospitalizations	0.10*	0.05 to 0.14	0.04*	0.00 to 0.08	0.04*	0.00 to 0.08	0.05*	0.01 to 0.09
COPD/asthma hospitalizations	0.01	-0.03 to 0.05	-0.01	-0.05 to 0.02	-0.01	-0.04 to 0.02	-0.01	-0.04 to 0.03
All-cause 30-day readmissions	0.53*	0.13 to 0.92	0.69*	0.34 to 1.04	0.66*	0.32 to 1.00	0.75*	0.41 to 1.08
ED Visits	0.24*	0.04 to 0.43	-0.35*	-0.52 to -0.18	-0.23%*	-0.40 to -0.07	-0.01	-0.17 to 0.16

Abbreviations: ACO, accountable care organization; CHF, congestive heart failure; COPD, chronic obstructive pulmonary disease; ED, emergency department; LDL, low-density lipoprotein.
†Adjusted risk differences and corresponding 95% confidence intervals expressed in percentage point changes.
*Indicates that a given predictive margin difference is statistically significant at $P<0.05$.

© 2022 Bakre S et al. *JAMA Health Forum.*

eFigure 1: Predicted Percent of Beneficiaries Receiving All Diabetes Tests at Each Percentile of Out-of-Network Primary Care Stratified by Racial and Ethnic Minority and White Beneficiaries in Low and High Racial and Ethnic Minority ACOs

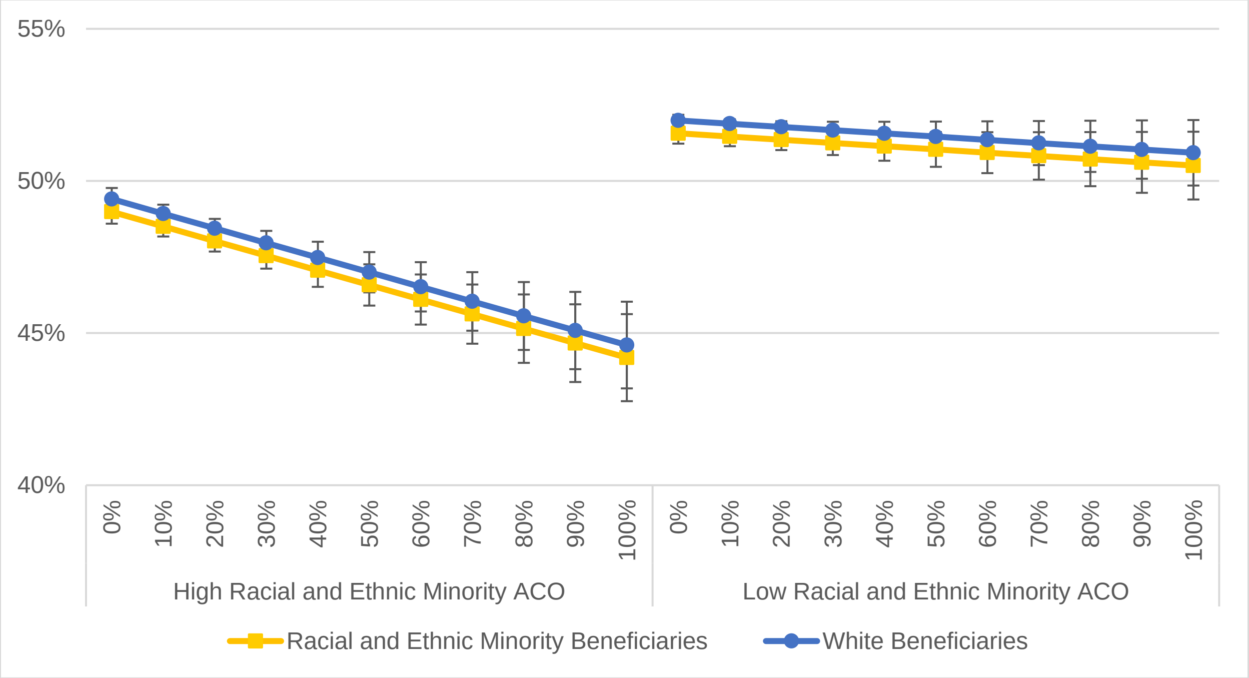

© 2022 Bakre S et al. *JAMA Health Forum.*

eFigure 2: Predicted Percent of Beneficiaries Receiving Diabetic Retinal Examinations at Each Percentile of Out-of-Network Primary Care Stratified by Racial and Ethnic Minority and White Beneficiaries in Low and High Racial and Ethnic Minority ACOs

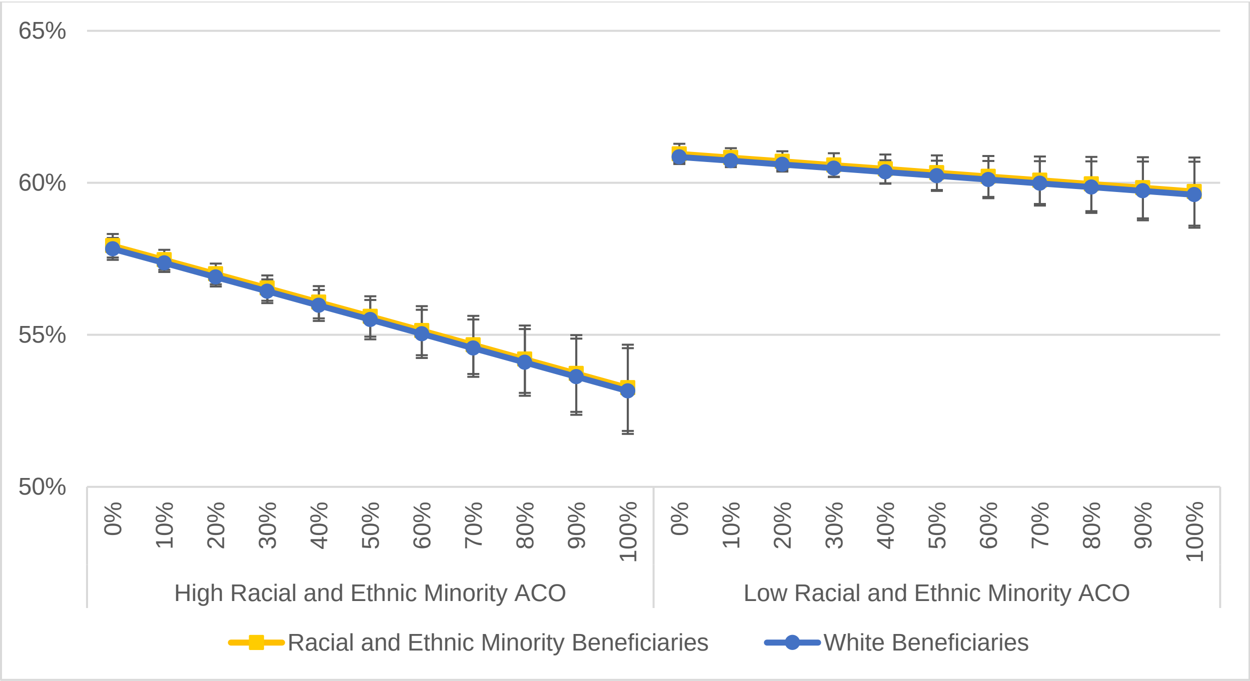

© 2022 Bakre S et al. *JAMA Health Forum.*

eFigure 3: Predicted Percent of Beneficiaries Receiving Glycated Hemoglobin Testing at Each Percentile of Out-of-Network Primary Care Stratified by Racial and Ethnic Minority and White Beneficiaries in Low and High Racial and Ethnic Minority ACOs

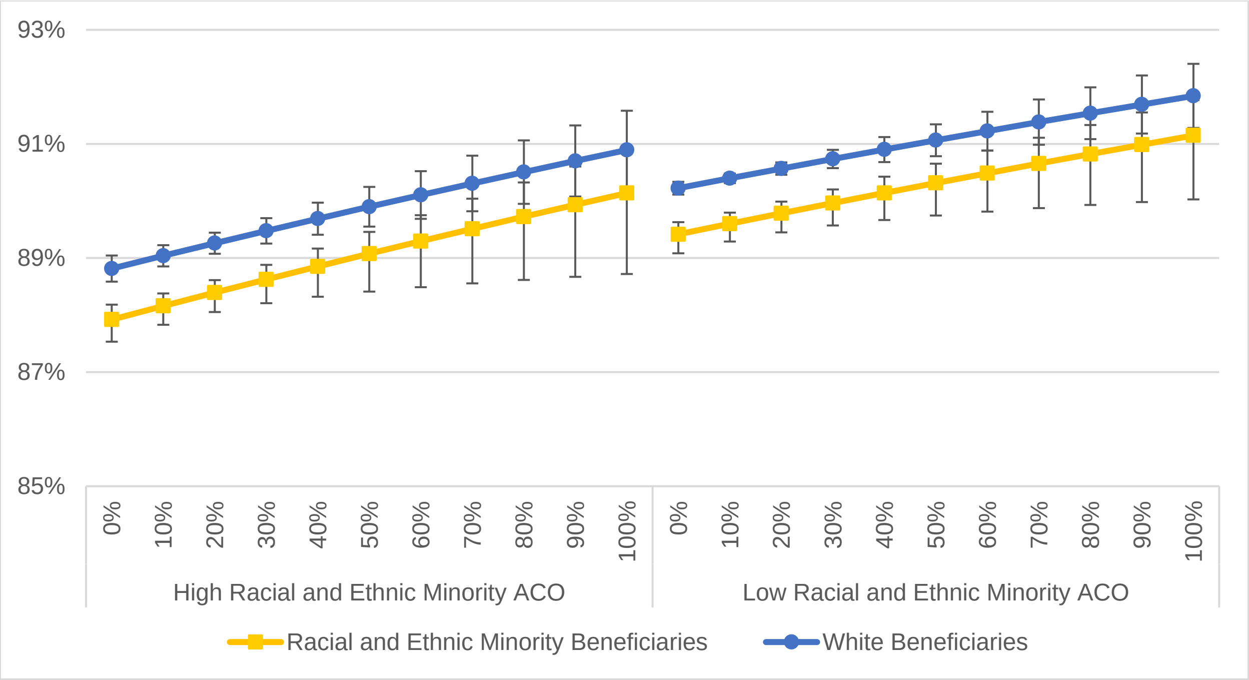

© 2022 Bakre S et al. *JAMA Health Forum.*

eFigure 4: Predicted Percent of Beneficiaries Receiving LDL Cholesterol Testing at Each Percentile of Out-of-Network Primary Care Stratified by Racial and Ethnic Minority and White Beneficiaries in Low and High Racial and Ethnic Minority ACOs

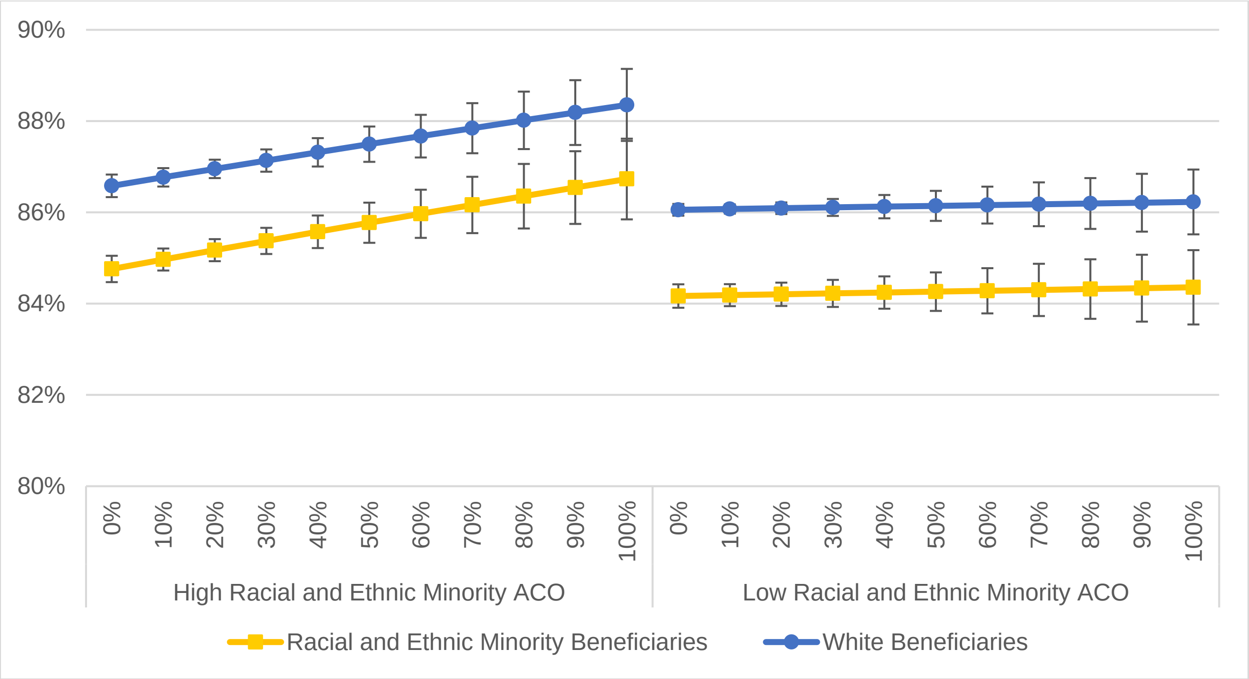

© 2022 Bakre S et al. *JAMA Health Forum.*

eFigure 5: Predicted Percent of Beneficiaries Receiving Mammography Screening at Each Percentile of Out-of-Network Primary Care Stratified by Racial and Ethnic Minority and White Beneficiaries in Low and High Racial and Ethnic Minority ACOs

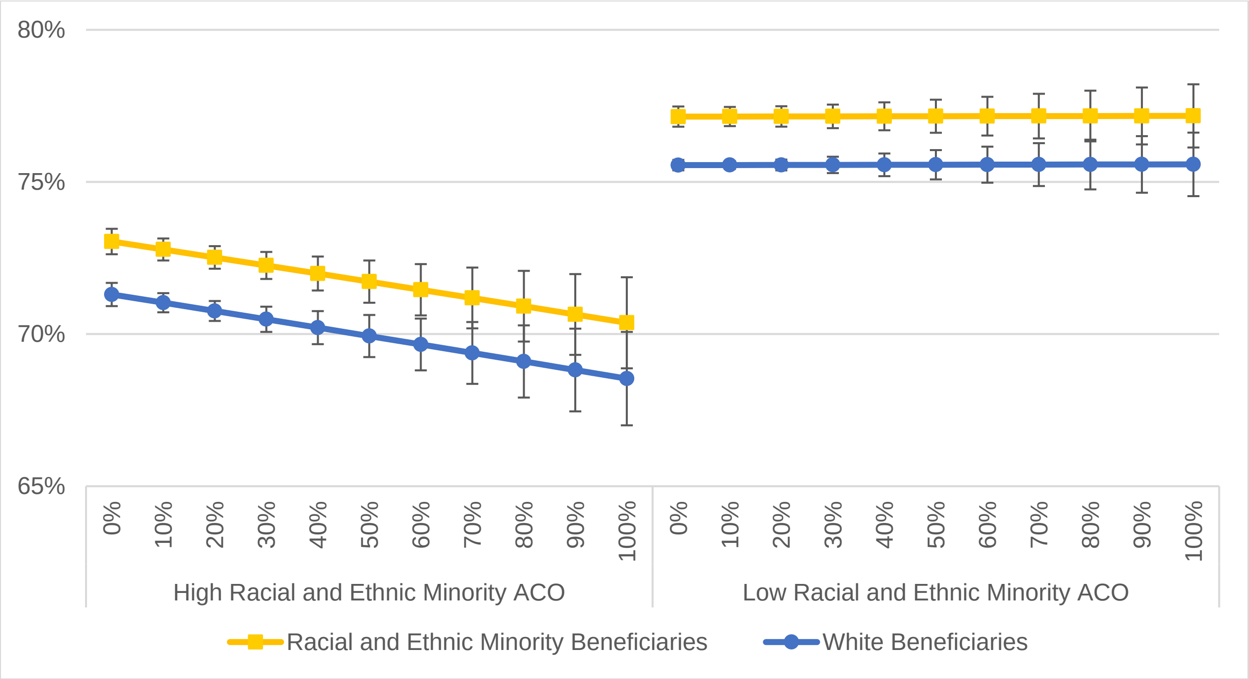

© 2022 Bakre S et al. *JAMA Health Forum.*

eFigure 6: Predicted Percent of Beneficiaries with Hospitalization for CHF at Each Percentile of Out-of-Network Primary Care Stratified by Racial and Ethnic Minority and White Beneficiaries in Low and High Racial and Ethnic Minority ACOs

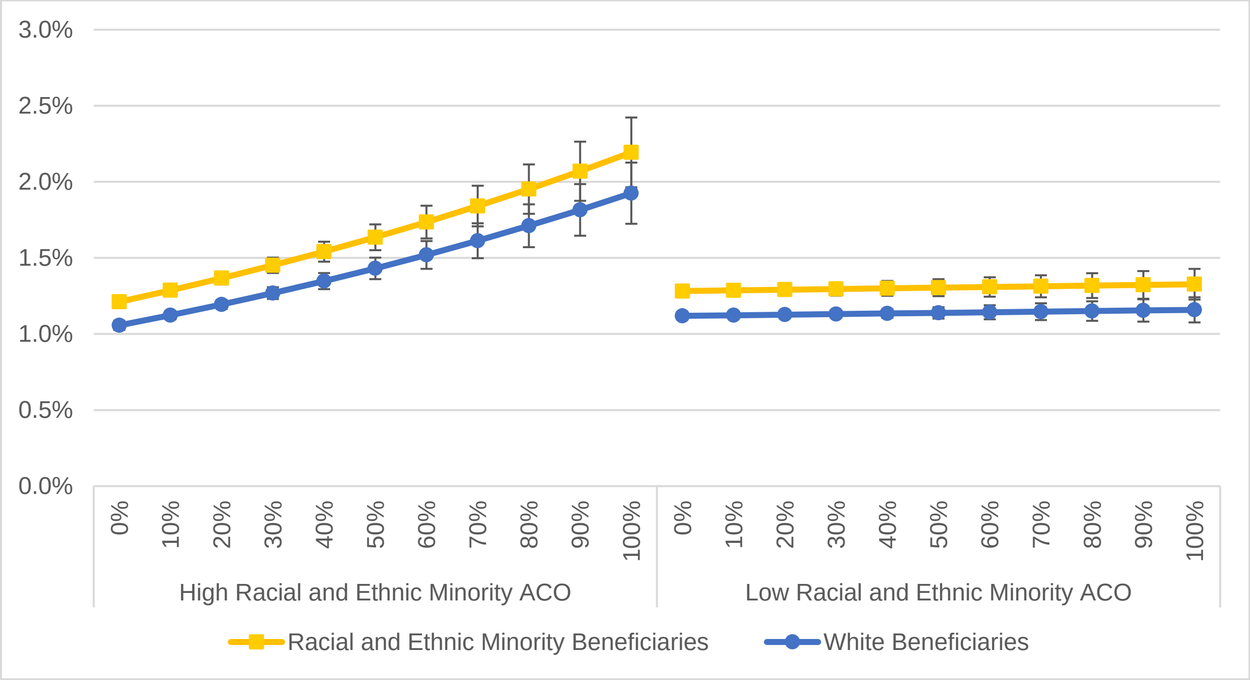

© 2022 Bakre S et al. *JAMA Health Forum.*

eFigure 7: Predicted Percent of Beneficiaries with Hospitalization for COPD at Each Percentile of Out-of-Network Primary Care Stratified by Racial and Ethnic Minority and White Beneficiaries in Low and High Racial and Ethnic Minority ACOs

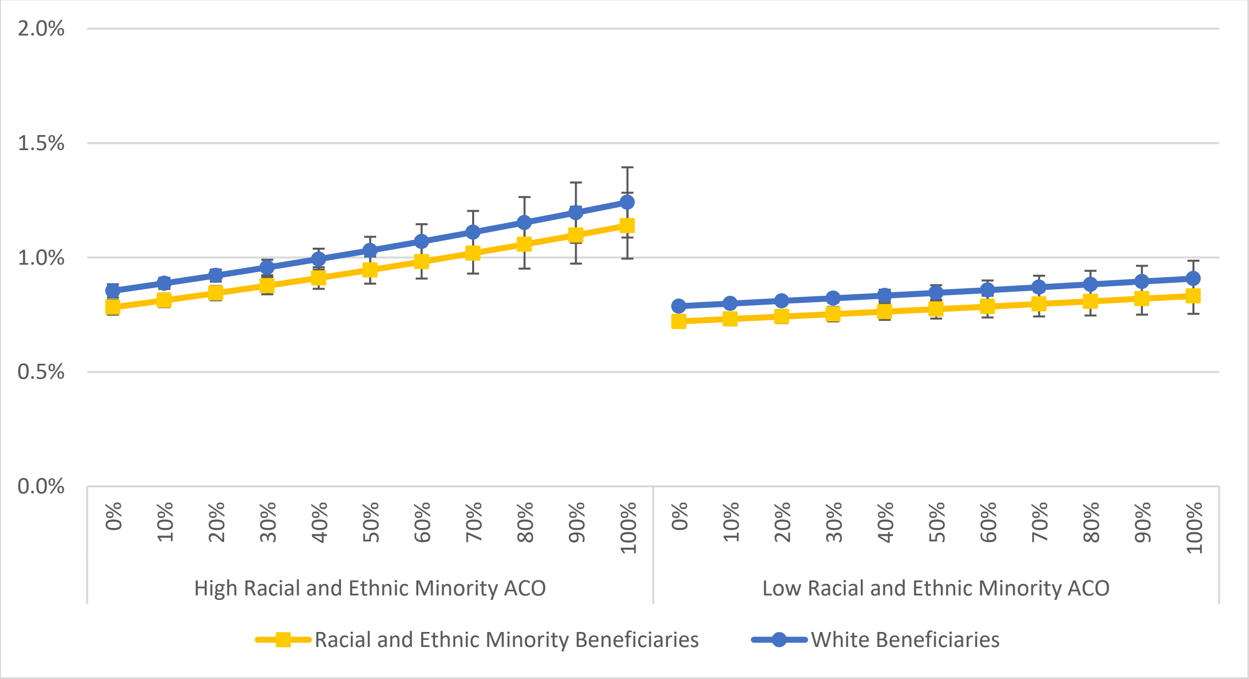

© 2022 Bakre S et al. *JAMA Health Forum.*

eFigure 8: Predicted Percent of Beneficiaries with a Readmission for Any Reason at Each Percentile of Out-of-Network Primary Care Stratified by Racial and Ethnic Minority and White Beneficiaries in Low and High Racial and Ethnic Minority ACOs

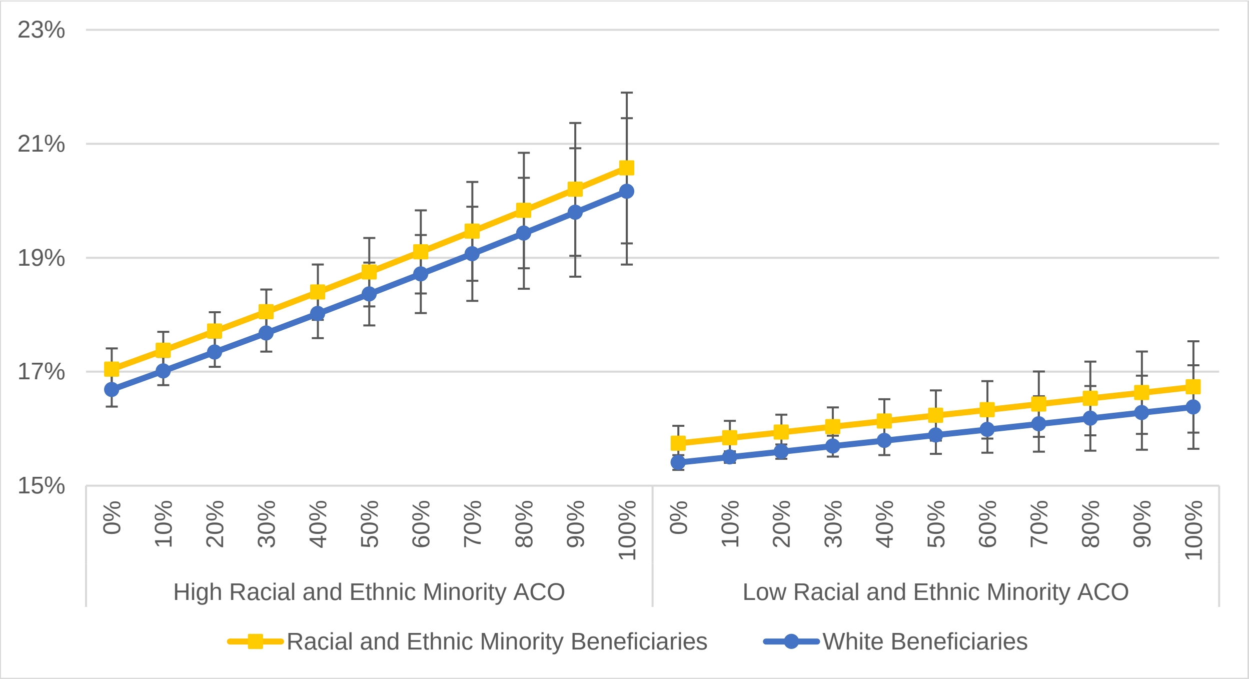

© 2022 Bakre S et al. *JAMA Health Forum.*

eFigure 9: Predicted Percent of Beneficiaries with an ED Visit for Any Reason at Each Percentile of Out-of-Network Primary Care Stratified by Racial and Ethnic Minority and White Beneficiaries in Low and High Racial and Ethnic Minority ACOs

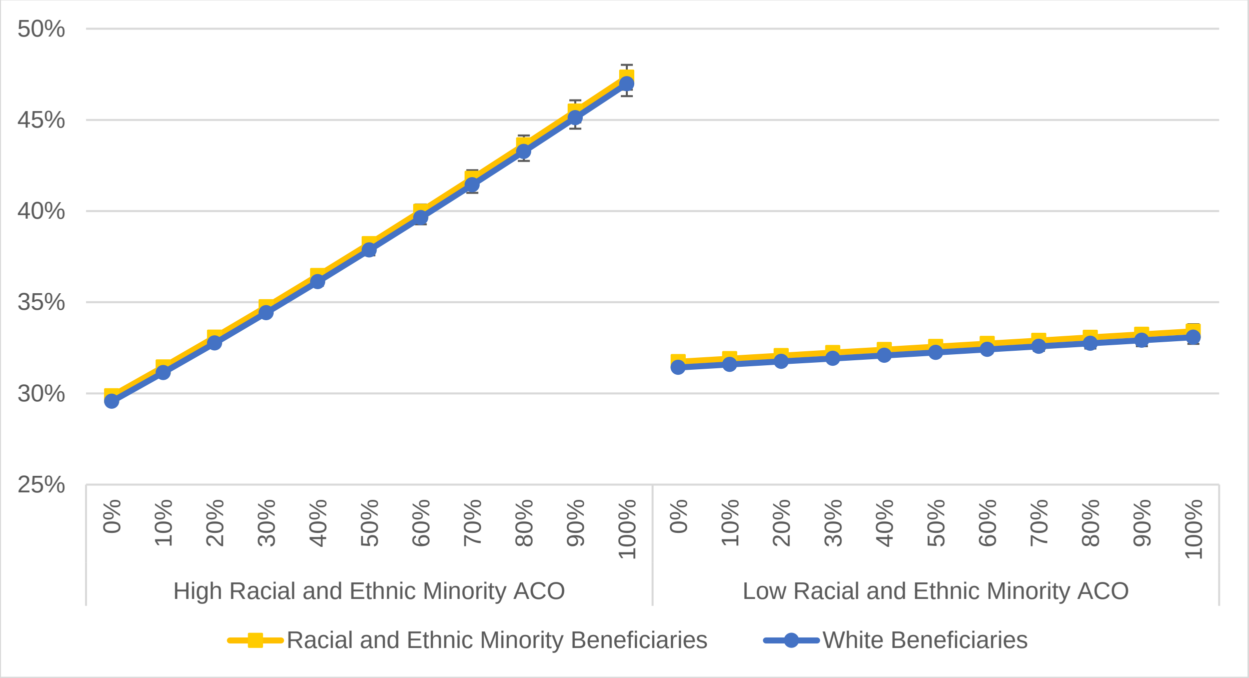

© 2022 Bakre S et al. *JAMA Health Forum.*
